# Hybrid Mathematical Model of Cardiomyocyte Turnover in the Adult Human Heart

**DOI:** 10.1371/journal.pone.0051683

**Published:** 2012-12-19

**Authors:** Jeremy A. Elser, Kenneth B. Margulies

**Affiliations:** 1 Department of Bioengineering, University of Pennsylvania, Philadelphia, Pennsylvania, United States of America; 2 Cardiovascular Institute, Department of Internal Medicine, University of Pennsylvania School of Medicine, Philadelphia, Pennsylvania, United States of America; Sapienza University of Rome, Italy

## Abstract

**Rationale:**

The capacity for cardiomyocyte regeneration in the healthy adult human heart is fundamentally relevant for both myocardial homeostasis and cardiomyopathy therapeutics. However, estimates of cardiomyocyte turnover rates conflict greatly, with a study employing C14 pulse-chase methodology concluding 1% annual turnover in youth declining to 0.5% with aging and another using cell population dynamics indicating substantial, age-increasing turnover (4% increasing to 20%).

**Objective:**

Create a hybrid mathematical model to critically examine rates of cardiomyocyte turnover derived from alternative methodologies.

**Methods and Results:**

Examined in isolation, the cell population analysis exhibited severe sensitivity to a stem cell expansion exponent (20% variation causing 2-fold turnover change) and apoptosis rate. Similarly, the pulse-chase model was acutely sensitive to assumptions of instantaneous incorporation of atmospheric C14 into the body (4-fold impact on turnover in young subjects) while numerical restrictions precluded otherwise viable solutions. Incorporating considerations of primary variable sensitivity and controversial model assumptions, an unbiased numerical solver identified a scenario of significant, age-increasing turnover (4–6% increasing to 15–22% with age) that was compatible with data from both studies, provided that successive generations of cardiomyocytes experienced higher attrition rates than predecessors.

**Conclusions:**

Assignment of histologically-observed stem/progenitor cells into discrete regenerative phenotypes in the cell population model strongly influenced turnover dynamics without being directly testable. Alternatively, C14 trafficking assumptions and restrictive models in the pulse-chase model artificially eliminated high-turnover solutions. Nevertheless, discrepancies among recent cell turnover estimates can be explained and reconciled. The hybrid mathematical model provided herein permits further examination of these and forthcoming datasets.

## Introduction

The human heart was long believed to have virtually no capacity for new cardiomyocyte (CM) formation occurring after childhood [1]. However, identification of myogenic progenitors within human hearts and Y-chromosome positive cardiac myocytes in sex-mismatched allografts from female donors raised doubts about the absence of new CM formation within adult human hearts [2]–[5]. A landmark study by Bergmann et al [6] utilized a pulse-chase approach to identify and quantify the rate of new CM formation in normal human hearts based on the average percentage of Carbon14 (C14) in the DNA of CMs in 12 human hearts (demographics reproduced in Table S1). Bergmann and colleagues compared the C14 content in cardiac myocyte DNA at the time of death to the known atmospheric concentrations of C14 at the time of the subject's birth (reproduced in Figure S1) [7], [8], with differences reflecting cell cycle activity as would occur during CM formation. Subjects born before the spike in atmospheric C14 were found to have more C14 in their CM nuclei than was present at time of birth while subjects born after the C14 spike had less C14 in their CM nuclei, suggesting new CMs had been created and incorporated new C14 into their DNA. Based on mathematical modeling that considered atmospheric C14, CM DNA C14 content, and DNA polyploidization estimates, Bergmann et al. reported a CM turnover rate of approximately 1% per annum during youth, *decreasing* to 0.5% during advancing age.

In 2010, Kajstura et al [9] performed extensive histological analyses of 74 non-diseased hearts of various ages to evaluate the presence of a proposed class of cardiomyocyte stem cells (CSCs) and the fraction of these cells undergoing mitosis at time of subject death. Supported by in vitro cycling and apoptosis kinetics parameters, mathematical modeling was used to compute the annual turnover rate as a function of subject age. Kajstura reported a turnover rate approximately 7% per annum in young adult males that *increases* more than 3-fold with advancing age, and even higher rates of CM turnover calculated for females.

**Figure 1:**
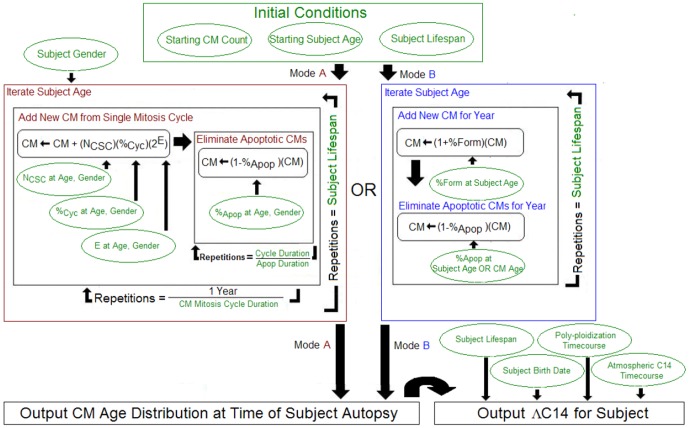
Hybrid Model Automaton Algorithm. Subject hearts are modeled by initiating a “Starting CM Count” for the subject at the “Start Age” of the simulation (typically birth) and a “Subject Lifespan” that determines the number of year-repetitions. An age distribution of CM at the time of subject autopsy is produced either via Mode A or Mode B. Mode A uses variables and formulas from the Kajstura methodology to perform iterations of CM creation and destruction throughout each year-iteration according to the duration and frequency of these cycles. In this formulation, N_CSC_ = Number of Cardiac Stem Cells, %_Cyc_ = Fraction of CSCs cycling, and E = Number of CSC divisions occurring before loss of pluripotency. Alternatively, Mode B uses input annual creation and destruction values directly and these values may be dependent on either the patient age (at time of CM formation or at current iteration of production/destruction) or on the age of the CM undergoing destruction. There are two main outputs: (1) a distribution of surviving CM by CM age and (2) an end average C14 measurement, modeling the Bergmann methodology applied to model hearts, which is produced by incorporation of the CM age distribution with atmospheric C14 timecourse data and human polyploidization magnitudes/rates.

While these two studies both indicate that new CM formation exists in the healthy, adult human heart, the discrepancy in magnitude and age progression is perplexingly large. Accordingly, we developed a hybrid mathematical model designed to evaluate the two models simultaneously. The hybrid model agent-based algorithm is described in Figure 1 and is programmed to accept turnover rate (CM formation, apoptosis rate, and polyploidization) parameters that vary with subject age and sex, such as those found in the Kajstura paper (Mode A); however, these variables may be substituted by time-varying CM formation and destruction rates as employed by Bergmann (Mode B). By applying this model to the two data sets, we have identified explanations for their discrepancies and defined whether differences can be reconciled, while providing a tool for modeling data derived from subsequent inquiries.

We first examine the Kajstura model in isolation. This section is divided into 3 main subsections: (A1) confirming the hybrid model's fidelity in re-producing identical conclusions (outputs) to the Kajstura manuscript when the Kajstura measurements (inputs) are used, (A2) evaluating the sensitivity of the Kajstura model to variations in input parameters and assumptions, and (A3) addressing important factors that were not considered in the Kajstura document. We then provide a similar three-subsection analysis of the Bergmann model in isolation with (B1) confirming the hybrid model fidelity to the Bergmann methodology, (B2) sensitivity analysis of the Bergmann model to its parameters and assumptions, and (B3) model modification based on previously unconsidered factors. Finally in subsection C, we seek turnover scenarios that are consistent with both datasets when reasonable estimates of input variable uncertainty are included and unproven assumptions are challenged and modified.

## Results

### A1: Hybrid Model Demonstrates Fidelity to Kajstura Model

The Kajstura manuscript proposes two complementary modeling methods—a hierarchically-structured cell kinetics model (controlled by CSC cycling rates and CM apoptosis rates) and an age-structured cell kinetics model controlled by CM half-life and apoptosis rates). We first verified that our hybrid model successfully reproduces the Kajstura hierarchically-structured model's age-dependent rates of CM apoptosis, formation, and turnover results when their primary input parameters (enumerated schematically in Figure 1), are used (Figure S2). We then validated the hybrid model's ability to recapitulate the age-structured cell kinetics model, which is responsible for generating CM age distributions for modeled subjects in the Kajstura manuscript. For any simulated subject, defined by (1) turnover rates as a function of time and (2) a selected subject lifespan, the model generates an age distribution of CMs present in the heart at the end of the simulation (time of subject death). These age distributions are also in excellent agreement with the Kajstura results for both genders (Figure S3). Thus, the hybrid model is a faithful means of probing the Kajstura analysis.

### A2: Parameter and Assumption Sensitivity in the Kajstura Model

Manipulating this hybrid model in hierarchical-structure mode demonstrates the sensitivity of modeling conclusions to variation in input parameters. Turnover estimates in the Kajstura model are exquisitely sensitive to estimates of the expansion exponent (the number of cell divisions a CSC is expected to undergo before losing regenerative capability), with a 20% variance in this parameter causing a 200% variance in estimated turnover (Figure S4). The Kajstura manuscript determined this expansion exponent by comparison of the relative abundances of CSCs to transit amplifying cells (classified by phenotypic markers) found in autopsy tissue. Such phenotypic categorization into discrete progenitor cell populations likely oversimplifies a more complex lineage progression. Moreover, the total CM counts over the course of a lifespan (accounting for proliferation and apoptosis) using Kajstura parameters, modeled female CM count increases with age, peaks in middle age, and then decreases but does not fall to levels of youth. Such a net increase in cardiomyocyte count is not consistent with the net decreases reported elsewhere [10]. Notably, applying a 20% decrease in the expansion exponent produces model hearts with a monotonically decreasing trajectory (Figure S5).

While the Kajstura turnover estimates are computed via the hierarchical-structured model using the expansion exponent and as a primary variable, the Kajstura CM age distributions are generated via the age-structured model using a CM half-life parameter that indirectly incorporates, though is separate from, the apoptosis duration parameter. This half-life is computed using a phenotyping technique similar to that used for the expansion exponent. The effect of variation in this parameter on the CM age distributions is shown for young, middle-aged, and older modeled hearts in Figure S6. A 20% variation in this parameter will cause as much as a 45% variation in average cardiomyocyte age at time of subject death.

Together, these analyses demonstrate that the CM turnover estimates in the Kajstura models are particularly sensitive to the magnitude of the expansion exponent (in the hierarchical model of CM turnover) and the CM half-life parameter (in the age-structed model of end-of-life CM age distributions).

### A3: New Considerations Applied to the Kajstura Model

**Figure 2:**
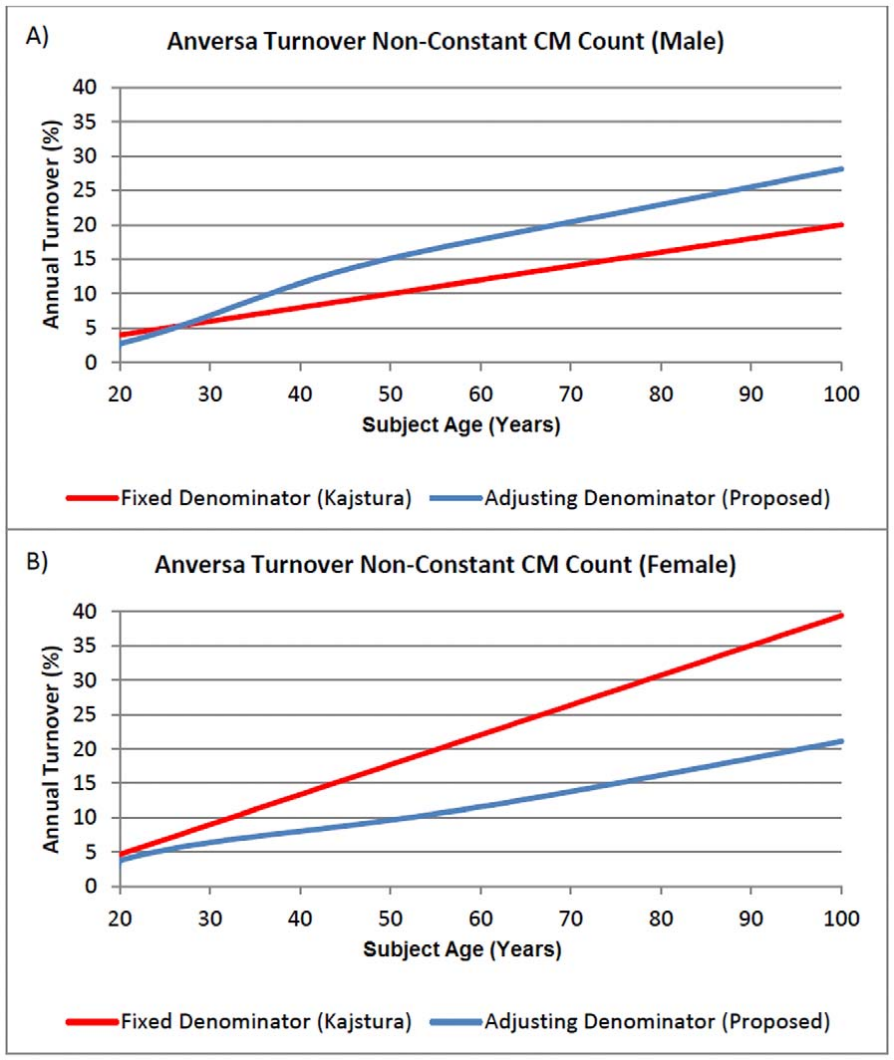
Apoptosis-Dependent Definition of CM Turnover in the Kajstura Model. The hybrid model was parameterized in accordance with the Kajstura published values for Cardiac Stem Cell number, cycling frequency, mitosis duration, and expansion exponenent. Turnover was computed as newly formed CM divided by either a constant CM density (5,000,000 CM per 10 g tissue, as in the Kajstura publication) or a variable CM density determined by iterative production and destruction of CM using the aforementioned Kajstura CM formation parameters in conjunction with the Kajstura CM apoptosis parameters. The simulation is performed for A) Males and B) Females.

Turnover estimates in the Kajstura model are also affected by assumptions about changes in total CM number as a function of age, because turnover is defined as the number of newly formed CMs divided by the total number of CMs present at time of formation. In the Kajstura plot of “annual turnover of myocytes” vs. subject age, dividing the regression line of the turnover plot by the regression line of the preceding CM formation plot indicates that a value of approximately 500 million CM per 10 g of tissue was employed by Kajstura et al as the CM number for all ages. However, the Kajstura primary input parameters do not concur with an assumption of constant CM presence and instead lead to fluctuations with age (Figure S5, blue traces). When the number of CM present is allowed to fluctuate as dictated by the Kajstura model and measured parameters, and when turnover is defined as the number of new CM formed in a given year divided by the number of CM present at that year in the subject's lifespan, turnover plots are as shown in Figure 2. Interestingly, the age-dependent decline in total CMs in male hearts amplifies the annual turnover in males while a net gain of CM suppresses the turnover rate in females.

In turn, a time-varying CM count allows examination of the impact of CM apoptosis on turnover rate. Although the Kajstura manuscript empirically measures apoptotic rate using TUNEL staining, alternative estimations of apoptosis using similar techniques have reported discordant results. One such study of human healthy human hearts by Mallet et al. found similar magnitudes of apoptosis but no age-dependency [11]. A version of the model using Kajstura parameters with the apoptosis rate substituted by Mallet is shown in Figure S7; such parameterization generates relatively low annual CM turnover for males and females (below 5%) declining with age to 2%–a finding larger but substantially more concordant with the Bergmann conclusions though primarily driven by large net gains in CM count.

The impact of technical factors on estimates of apoptosis rates also merits consideration. Peri-mortem increases in tissue TUNEL staining becomes significant around 24 hours post-mortem and can double the apparent apoptotic fractions (highly tissue dependent) [12]. Alternatively, TUNEL staining has been shown to underestimate apoptosis (particularly when high levels of necrosis occur) with sensitivities ranging from 60–90% [13]. Therefore, Figure S8 illustrates the impact of potential apoptosis variation in the Kajstura model. The absolute rate of apoptosis has a roughly linear impact on turnover estimation. Notably, at extremely small multiples of apoptosis fractions found by Kajstura (but with the same age-increasing trajectory), reported turnover in the Kajstura model becomes low and age-decreasing, albeit primarily dominated by a net gain in CM content that serves as the denominator in this formulation of the turnover definition.

### B1: Hybrid Model Demonstrates Fidelity to Bergmann Dataset

The Bergmann model relies on measured C14 content in CM DNA at time of autopsy as the primary input variable, rather than cell formation/destruction parameters utilized by Kajstura. To account for the impact of age-dependent DNA polyploidization within CM nuclei on C14 content, Bergmann integrates prior data from Adler et al [14] to conclude that during childhood the vast majority of CM nuclei become multiploid. These polyploidization events incorporate C14 in a manner that mimics true cardiomyogenesis and are thus subtracted from the Bergmann analysis model. Depending on subject birth and death years (and the intervening atmospheric C14 concentrations), 30–100% of measured ΛC14 (varying by subject) is attributed to polyploidization in the Bergmann study of 12 subjects, directly reducing the estimate of CM turnover.

The hybrid model, parameterized with CM formation/destruction rates, produces CM age distributions for simulated subjects as described above (schematized in Figure 1, Mode B). Based on the subject's birth date,the hybrid model identifies the birth years of CMs remaining at the end of the simulation with atmospheric C14 levels at various points in history (reproduced in Figure S1) to calculate an associated ΛC14 value for each CM surviving to the end of the simulation (i.e. those that contribute to simulated ΛC14 value). To complete the computation of simulated ΛC14 for a modeled pateitn, an age-dependent polyploidization compensation factor must be supplied; the magnitude and age-dependency of this polyploidization compensation is a function of both the Bergmann/Adler data for human CM poly-ploidization and also the simulated CM formation/destruction rates (unique to each simulation). Thus, the hybrid model can be used to determine ΛC14 levels that would be expected in a subject undergoing specified turnover/polyploidization dynamics.

In some instances, the hybrid model is used to determine the turnover scenario (age-dependent CM formation/destruction rates) that best explains a measured ΛC14 value (as recorded in the Bergmann 12-subject dataset). A numerical solver was constructed to produce a large number of candidate models (using wide ranges for each parameter) and identify scenarios that best fit the measured ΛC14 value.

The hybrid model fidelity to the Bergmann analytical system can be shown in a 3-part process outlined schematically in Figure S9. Briefly, the subject-specific constant annual CM turnover rate and polyploidization rate measurements obtained by Bergmann were substituted as input for the cellular formation/destruction parameters. Subject-specific CM age distributions were built from this parameterization scheme, and simulated end C14 levels were produced from these age distributions by cross-referencing historical atmospheric C14 levels. Under these conditions, the hybrid model produced faithful estimates of end C14 (within 10% of the Bergmann measured raw values from autopsy). Furthermore, the hybrid model polyploidization compensator module, derived from the Bergmann/Adler composite polyploidization measurements, performs identical compensation to that applied by Bergmann (Figure S10B). Finally, the numerical solver constructed for the hybrid model that converts subject-specific ΛC14 values (obtained by subtracting initial C14 values, equivalent to atmospheric C14 at time of birth, from the end C14 modeled values) into estimates of annual CM turnover also functioned identically to the Bergmann system with no distortions, as shown by the solver's ability to convert the simulated ΛC14 values into the original input turnovers (Figure S10C). Thus the hybrid model application to C14 data provides an excellent representation of the Bergmann analytical system without distortion.

### B2: Parameter and Assumption Sensitivity in the Bergmann Model

Because the polyploidization correction factor is used to directly minimize the concluded annual turnover level, precision in this estimate might be important. In fact, other studies provide diverse, generally reduced (compared with Bergmann/Adler) values for poly-ploidization in healthy adults [15]–[18]. In one representative study by Takamatsu et al, human age-dependent polyploidization was found to follow a sigmoidal time course with poly-ploidization essentially completed during adolescence and ultimately resulting in an average cardiomyocyte DNA content of approximately 3.0*n* (versus 3.8 for Bergmann/Adler) [19]. Substituting the a poly-ploidization correction based on the Takamatsu data (about 20% less than the Bergmann correction factor), rather than using the more severe Bergmann correction, results in a modest 25% increase in the Bergmann turnover estimates for younger subjects from 1.3%±0.2% annually under the Bergmann estimate to 1.6%±0.1% under the Takamatsu estimate, p<0.15), while estimates of turnover for the 4 oldest subjects, born such that polyploidization occurred prior to the atmospheric C14 spike, are largely unaltered (Figure S11A and S11B).

### B3: New Considerations Applied to the Bergmann Model

The Bergmann manuscript makes the simplifying assumption that C14 incorporated into DNA corresponds instantaneously to the atmospheric concentration at the time of cell formation,however, this assumption is not warranted. Relevantly, Broecker et al. analyzed C14 incorporation into the food supply and human tissue and concluded that there is a delay of up to 2 years for solid tissue [20]. Such a delay substantially affects turnover levels in subjects meeting both of two criteria: (1) true turnover is low (∼1% annually) such that hearts at time of subject death are substantially composed of original CMs and (2) subjects are born in a time of rapidly changing atmospheric C14 such that a small delay results in substantially under-estimated initial C14 values. Thus, while the older (pre-bomb subjects) are largely unaffected by delays of atmospheric C14 incorporation into CM DNA, younger subjects are highly susceptible. Among the 5 youngest subjects in the Bergmann dataset, a 2-year delay causes average turnover conclusion to increase from 1.4%±0.1% to 5.7%±0.8%, p<0.001 (Figure S12A).

Similarly, the atmospheric C14 measurements from the Levin dataset used by Bergmann^7,8^ show wide monthly variations that indicate the variability of the metric. As a further illustration of the sensitivity of C14 tracer studies to initial conditions, the hybrid model was seeded with initial C14 values for each subject taking on either the highest or lowest C14 value obtained within 12 months of subject birth and results are shown as error bars upon the concluded turnover obtained when the 1 year smoothing function is applied (Figure S12B); extreme fluctuation is observed ranging in nearly all subjects from 0% annual turnover to several multiples of the smoothed-C14 conclusions.

The 3 oldest subjects (ND60, ND67, and ND73) in the Bergman dataset are interesting for several reasons. The analysis of these subjects, born in 1933, 1939, and 1944 respectively, are independent of complications from polyploidization and initial C14 levels as the increase in atmospheric C14 takes place after their adolescence. Based on the fact that the measured C14 values for these subjects is lower than contemporary atmospheric conditions, Bergmann deduced that a large fraction of CMs produced either at birth or shortly after (prior to the atmospheric C14 spike), must persist until death. Accordingly, these subjects were computed to have undergone very low annual turnover rates of 0.10%, 0.09%, and 0%, respectively, by Bergmann (under the Bergmann “Scenario A” constant turnover scenario). For example, the meaning of this metric suggests that for the oldest subject, during each year from birth to death, 0.10% of CM were replaced at random by newly-formed CM, so that at the end of the subject's 73 year lifespan, 90% of original CMs would remain and the 10% of CM that were created post-birth raise the measured average C14 level to 21.30.

However, the younger subjects (aged 19–43 at time of death) all report average annual turnover values ranging from 1–2%. In contrast to the Scenario A assumptions, a true model of turnover ought to require that older subjects go through a phase in youth that is similar to that of the youthful subjects, i.e. that subjects ND60, ND67, and ND73 each experienced phases of their lives (at least from age 0–40) where turnover was on the order of 1–2% annually. Such turnover levels, even allowing turnover to cease entirely at age 40, would have reduced the final contribution of initial CMs in these 3 subjects to 65% of the total CM count, substantially less than the claimed approximately 90% under Scenario A. Indeed, such turnover would lead to ΛC14 values of 62, 77, and 84 per mil (final average C14 less initial at birth), far greater than the measured 21.3, 18.84, and 3.65. Allowing turnover to continue beyond year 40 actually serves to partially reconcile the simulated ΛC14 values. Indeed, allowing for elimination of the high-C14 CMs, generated during the atmospheric C14 spike, via increased turnover provides better agreement with measured subject C14 levels (although, as contemporary C14 levels never fall below the observed ΛC14 results, no amount of turnover can fully resolve this paradox).

Bergmann also highlights a modified version of Scenario A in which total cell count is again constant (full CM replacement) but turnover can change with subject age. The global solution (derived from C14 measurements in all 12 subjects) was:
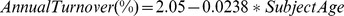



However, for all 3 of the eldest study subjects, ΛC14 measurements should have exceeded 100 per mil (101, 110, 116 for ND60, ND67, and ND73, respectively), several multiples above the measured. This model also demonstrates the inherent incompatibility of C14 measurements in the oldest subjects with C14 measurements in the younger subjects.

Alternatively, the scenario ultimately favored by Bergmann, denoted “Scenario E2,” allowed CM destruction rates to be inversely proportional to CM age while time-varying CM formation rates balanced destruction to keep a constant total CM count. Formulaically, the model is represented below (where “t” represents the subject age and “a” represents the age of a given CM:

Time Dependent CM Formation Rate:
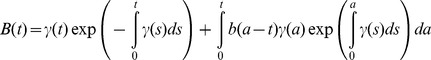



Age Dependent CM Destruction Rate:
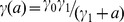



In this model, younger CMs (when the CM's “a” is low regardless of the subject year “t” in which it was formed) are destroyed more frequently than CM that have persisted to an older age “a.”

This model, when fitted with γ_0_ = 0.123 and γ_1_ = 1.42 as dictated by Bergmann, yields turnover values with magnitude and age-dependency similar to Scenario A. The expected end C14 values for ND60, ND67, and ND73 under this scenario are 58, 68, and 82 per mil, respectively, which mitigates the inconsistency but still drastically exceeds the measured values.

**Figure 3:**
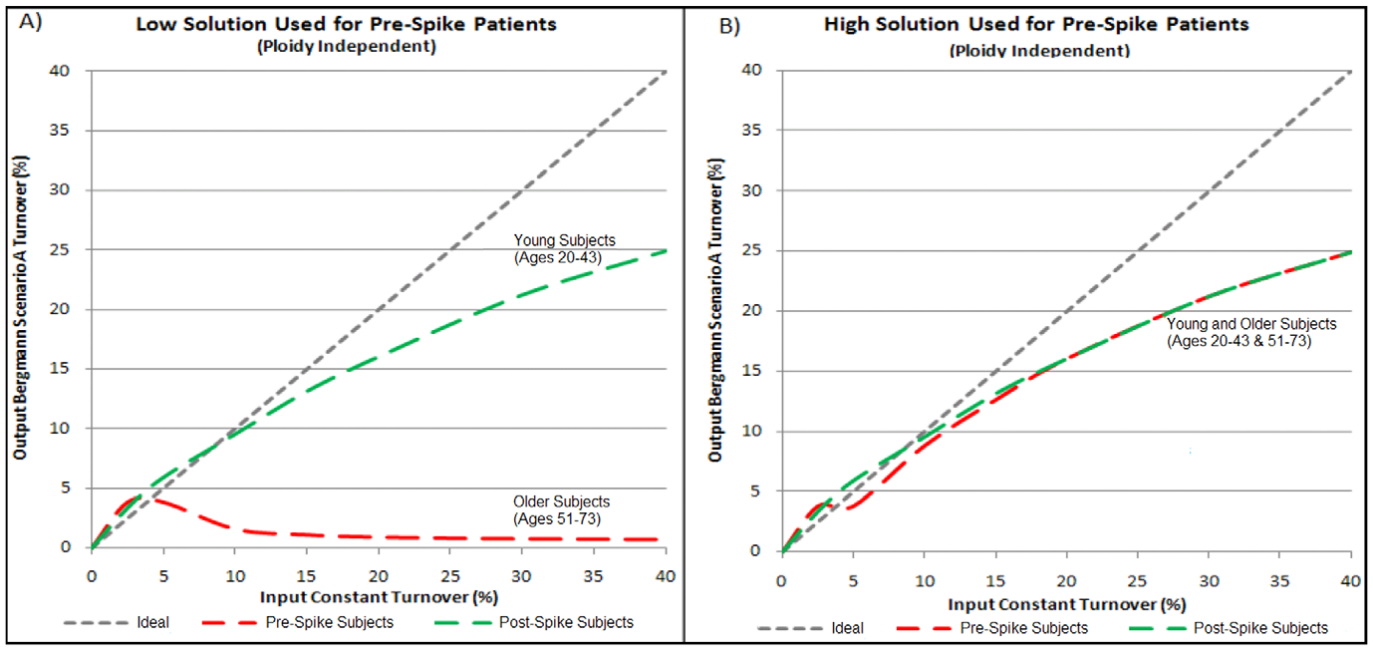
Simulations Derived from Applying Pre-specified Annual CM Turnover Rates to Bergmann Model Analysis Method. A model was produced for each of the 12 Bergmann subjects (defined by their lifespans and birth years). Instead of cycling dynamics parameters from the Kajstura manuscript, true constant annual turnover was specified as input. The category “Older Subjects” includes ND60, ND67, ND73, ND61, and ND51 from the Bergmann study, while the category “Young Subjects” includes ND56, ND68, ND50, ND69, ND71, ND54, and ND74. When true turnover input is high (beginning at 4%/year and fully apparent by 10%/year), the resulting ΛC14 has two numerical solutions—a low solution (plotted in A) and a high solution (plotted in B). The high solution tracks the true (ideal) input whereas the low solution asymptotes at 0.75%/year. Although the ΛC14 levels obtained by Bergmann for older subjects (∼20 per mil) are too low for the high solution to be enabled (∼60 per mil, not shown), this graph illustrates that, for measured ΛC14 exceeding a certain discrete threshold, a bifurcation point exists such that adoption of the low solution leads to insensitivity.

In addition, for subjects born before the atmospheric C14 spike, at a certain subject-specific value of measured ΛC14, the numerical solution bifurcates creating low and high solutions. Beyond the bifurcation point, the low solution reports a turnover estimate of 0.75%/year even as higher turnover rates are used as the sole input parameter, whereas the high solution remains approximately truthful to the ideal at all turnover levels (Figure 3). The hybrid model then computes estimated ΛC14 for each subject under each turnover input scenario and then solves for a best-fit constant turnover solution using either using either the low or high solution. While the measured ΛC14 for subjects born before the C14 spike is below the threshold needed to make a high solution viable, the high solution becomes viable at surprisingly low ΛC14 measurements (approximately 60 per mil), lower than the ΛC14 measurement that could have been expected in these subjects from the global Scenario A best-fit data (by which Bergmann concluded 1% annual turnover).

Combining (1) the fact that the low measured C14 values observed in the 3 oldest subjects (used by Bergmann to eliminate the high solution) are incompatible with measured C14 values from the younger subjects with (2) the observation that a high turnover solution can yield relatively low C14 measurements, a high-turnover scenario for elderly subjects becomes reasonable.

### C: Turnover Models Simultaneously Compatible with Bergmann and Kajstura Datasets

The prior results demonstrate that both models have reasonable uncertainty in concluded turnover estimates. In the Kajstura manuscript, these uncertainties stem primarily from strong sensitivity to primary input variables (particularly ones that can only be measured indirectly). In the Bergmann manuscript, uncertainty arises both from a conspicuous inconsistency in data from old versus young subjects and also from assumptions regarding the incorporation of atmospheric C14 into CM DNA. In section C, we attempt to vary key input parameters and modify tenuous model assumptions to find scenarios that are simultaneously compatible with both datasets.

To test new models beyond the original 8 evaluated by Bergmann, we constructed a global numerical solver that allows all linear turnover solutions to be evaluated. The 4-parameter generic model, denoted as the Time-Varying Birth Rate/Time Varying-Death Rate (TVB-TVDR) model, assumed a linear dependence of CM formation rate and CM annual destruction rate on subject age. In this scenario, death rate controls not the fractional destruction of CM at Subject Age, but the rate at which CM produced at that Subject Age will be destructed annually such that CM formed at different times may be destroyed at different rates), thus generating the parameters B_s_, B_i_, D_s_, and D_i_ as shown:
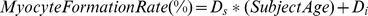


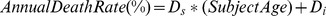



In this construction, the annual CM formation rate at birth is given by B_i_, and is increased or decreased to a new value in each subsequent year as dictated by the value of B_s_. Thus, in a linear model, B_i_ is the “Intercept” and B_s_ is the “Slope with respect to subject age.” Each CM, once formed, is assigned an annual death rate dependent on the year of the subject's life in which it was formed such that a cohort of CM formed during a certain year of a subject's life will be fractionally depleted (as determined by that cohort's computed Annual Death Rate, which has a slope D_s_ and intercept D_i_) in each subsequent year of subject life. Notably, this model differs conceptually from the Bergmann's E2 model, which allowed each CM (regardless of when it was formed in the subject's life) to experience a death rate dependent on the age of the CM. To clarify, the Bergmann E2 model assumes that every CM experiences an identical “death likelihood trajectory” regardless of how old the subject was at the time of formation whereas the TVB-TVDR model assumes that CM formed at different times in the subject's life are fundamentally different (experience different annual death rates).

To verify the fidelity of the numerical solver and thus justify its ability to compare the best fit of models from the Bergmann manuscript to new, less-restrictive TVB-TVDR models, we recapitulate various Bergmann models and demonstrate that our solver produces identical best-fit results (Methods S1).

Of the 8 scenarios tested by Bergmann, no model allowed CM formation rate to change with subject age while simultaneously allowing new CMs to experience destruction rates dependent on subject age at time of formation. Because the Kajstura data suggests that CM formation increases with age, but that CMs formed in advanced age are shorter-lived, we evaluated such a model using the global solver by allowing all 4 parameters of the TVB-TVDR to vary independently. To compare the fit quality of models with different numbers of parameters, we compute the Akaike Information Criteria (AIC) value for the Sum of Squared Error (SSE) associated with each model using the formula employed by Bergmann; this formula essentially penalizes models with many parameters to give an adjusted fit values (with low AIC scores suggesting better fit).

The global best fit for the TVB-TVDR model was B_s_ = 0.10%, B_i_ = 2.5%, D_s_ = 0.5%, D_i_ = 1.0% and the SSE = 5589 (AIC = 3.16)—a scenario of age-increasing turnover but where newer CM are destroyed more quickly than older CM. This and other scenarios report superior AIC scores than the constant turnover scenario (Scenario A) of (B_s_ = D_s_ = 0, B_i_ = D_i_ = 0.99%), which yielded AIC = 3.18 in the Hybrid Model. Similarly, the TVB-TVDR model achieves better fit than Bergmann's preferred model of Cumulative Survival with Cell Replacement Inversely Proportional to Cell Age (denoted as “E2” in the Bergmann supplementary information), which yielded a SSE of 29,578 (AIC = 3.56). The end C14 values produced by the best-fit TVB-TVDR model are greatly superior for each subject (including the critically important most elderly 3 subjects) than either the Bergmann or Kajstura models (Figure S13).

**Figure 4:**
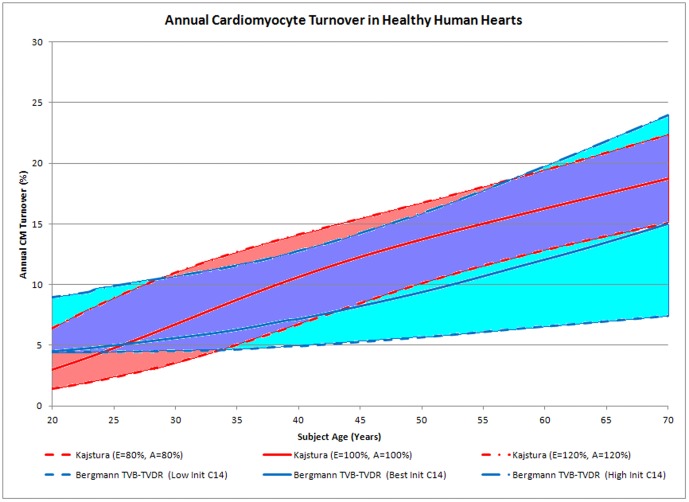
Impact of Assumptions and Parameter Measurement Uncertainty on Two Independent Models of CM Turnover. Turnover rates derived from Kajstura data plotted (red solid) with a range of reasonable uncertainty created by varying the primary model variables (CM Apoptosis Frequency “A” and Expansion Exponent, “E”) simultaneously by ±20% (red dashed) under the adjusting CM count scenario (wherein the number of CM cells varies in time according to the computed CM formation/destruction rates) with gender weighting as equivalent to the gender-weighting of the Bergmann dataset (75% male, 25% female). Turnover rates derived from Bergmann data, when conclusions from the TVB-TVDR model, parameterized by optimum fit to the 12 Bergmann subjects ΛC14 data, are shown (blue solid) with a range of reasonable uncertainty created by performing the TVB-TVDR global fit function with initial C14 levels either set to the lowest or highest within 1 year of each subject's birth (blue dashed). A region of overlap, indicating turnover levels consistent with both the Bergmann and Kajstura datasets, is indicated in purple.

Figure 4 describes the relationship between CM turnover and subject age. The TVB-TVDR best fit model is parameterized as described and polyploidization correction is performed as according to the Bergmann/Adler estimates. Notably, no Kajstura turnover dynamics are incorporated in the creation of the TVB-TVDR dataset. The best fit model (with age-increasing turnover) is displayed as a solid blue line, though other models with similar fit have been discussed. The zone of models compatible with Bergmann datasets are determined by substituting the initial C14 values for each subject with either the higher or lower of initial C14 values found within 1 year of the subject's birth to roughly illustrate precision. In both cases, turnover is defined as the number of new CM formed in a given year divided by the number present in the heart at that year.

For comparison, the Kajstura turnover estimation using the adjusting turnover definition is also shown in Figure 4 with variations to its key parameters. Variation in apoptosis frequency rate affects turnover conclusions primarily in aged subjects with little influence in youth (Figure S14). Alternatively, variation in expansion exponent affects turnover conclusions primarily in youthful subjects and does not influence turnover conclusions at old age (Figure S15). While the expansion exponent drastically affects CM formation at all ages, particularly in old age (Figure S4), this effect is roughly offset by cumulative CM count changes (Figure S5) yielding little impact on turnover. While Figures S14 and S15 explore the variable sensitivity in a gender-specific manner, Figure 4 displays a gender-composite computation for comparison to the Bergmann simulations. As the original Bergmann manuscript amalgamated data from 9 males and 3 females, the Kajstura best estimate trajectory shown in Figure 4 is also computed as the weighted composite of the male (75% weight) and female (25% weight) turnover trajectories (red solid line) with variations in parameters also plotted.

When both models incorporate reasonable estimates for variations in their primary parameters (CM Apoptosis Rate and Expansion Exponent for Kajstura, Initial C14 parameter for Bergmann), a region of agreement emerges. Figure 4 suggests that initial turnover levels of 4–6%, increasing with age to 15–22%, are compatible with both datasets.

## Discussion

In this analysis, we have rigorously investigated the intricacies and impact of assumptions in two primary estimations of CM turnover in healthy adult human hearts. While additional complications regarding biological methodologies have been proposed both by authors affiliated with the Bergmann and Kajstura manuscripts [21], [22] and also by independent investigators [23], [24], we find that mathematical considerations are powerful components of the conclusions that can be drawn from these studies.

Kajstura et al concluded that younger subjects experience annual turnover of approximately 5–8% (with no statement about turnover during childhood up to 20 years of age). Bergmann et al concluded an annual turnover rate of 1–2% for these younger subjects, a result not far removed from Kajstura when consideration of axioms, methods, and parameter sensitivity are included (Figures 2, S5, S6, S7, S8, S11, S12, S14, S15).

Discrepancy arises primarily in the older subjects, where Kajstura concludes age-increasing turnover (approximately 20% in aged males, higher in females), Bergmann concludes age-decreasing turnover (approximately 0.5%). The CM formation rates in the Kajstura hierarchically-structured model is highly sensitive to a stem/progenitor cell expansion exponent calculated indirectly from phenotypical lineages. Furthermore, the definition of the turnover formula itself is open to interpretation. In the Kajstura paper, a constant baseline value of CM content (which becomes the denominator in the formula “Turnover = CM Formation/CM Content)” is used for subjects of all ages thus allowing turnover plots to be wholly dictated by CM formation rates. However this constant value is inconsistent with the measured CM formation and destruction parameters obtained by the Kajstura experiments. Similarly, peri-mortem increases in apoptosis and imperfect sensitivity of the TUNEL staining assay may further have confounded estimations of apoptosis; extreme apoptosis over-estimation by Kajstura could potentially have masked lower levels of turnover particularly in older subjects. While the question of proper apoptosis valuation persists, this examination demonstrates the strong impact that subtle and often over-looked differences in definitions and parameter selection have on iterative mathematical models.

Similarly in the Bergmann model, the assumption of instantaneous incorporation of atmospheric C14 into human tissue, which is not supported by prior studies,^20^ may have caused an underestimation of turnover in young subjects. Regarding aged subjects, Bergmann makes the logical deduction that end C14 measurements for the 3 oldest subjects, which are below atmospheric C14 levels at all points after the rise in atmospheric C14, implies that a large number of initial CM (created peri-natal) must persist to the end of subject life. Bergmann goes on to deduce that a large number of initial CM surviving to death implies low turnover rates throughout life; however a linear model that allows high and age-increasing turnover can also preserve peri-natal CMs by allowing newly-formed CMs to experience a higher rate of attrition than older CMs. Indeed, such a model provides superior fit both globally and for each individual subject in the Bergmann dataset than constant turnover scenarios with reasonable associated trajectories for total CM count.

An additional insensitivity effect may have contributed to the conclusion of low-turnover in advanced age. The ability of high-turnover scenarios to fit low ΛC14 observations for subjects born before the spike in atmospheric C14 may appear paradoxical, but high turnover solutions in general can decrease expected ΛC14 measurements by eliminating CMs containing copious amounts of C14 absorbed during the atmospheric C14 peak. Such an effect is manifested in Figure 3, which shows that annual turnover beginning at 4% would generate ΛC14 values that would support two solutions—a low solution and a high solution. Thus, at surprisingly low ΛC14 measurements, the appropriate level of turnover to conclude from such measurements exhibits a bifurcation—a radically higher solution can be appropriate for a modestly higher ΛC14 measurement. If the Bergmann measurements for the 3 eldest subjects are accurate representations of true CM DNA content at time of death, then this high solution is irrelevant as C14 levels never decline below the measured ΛC14 values during the lifetimes of these subjects. However, the credibility of these low measurements is made suspect by the fact that ΛC14 measurements from the younger subjects imply turnover levels that ought to (under a low, constant turnover scenario), have produced drastically higher ΛC14 values than were observed in these older subjects.

Several scenarios achieve similar fit to the Bergmann C14 measurements; however the TVB-TVDR model emerges as the best-fitting scenario with global best fit parameters fitting the end C14 values of each subject better than either the Bergmann or Kajstura models without invoking unreasonable total CM count trajectories (Figure S13). Notably, the TVB-TVDR model, in many best-fit and near-best-fit parameter solution sets, also concurs with the conclusion reported by Kajstura that CMs formed later in life experience diminished longevity compared to predecessor CMs, despite the fact that no Kajstura data was used to obtain these solutions.

When attempts are made to incorporate reasonable measurement and assumption uncertainty into the Bergmann and Kajstura models, a range of turnover parameters emerges that are compatible with both datasets—specifically, that turnover in the healthy human heart is approximately 4–6% in youth increasing to 15–22% with advancing age.

Within our own analysis, we recognize several limitations. Our analysis is performed using the two most prominent manuscripts investigating turnover in healthy human hearts. While these papers are particularly targeted to the scientific question, other studies involving animals or other patient populations (particularly those with relevant morbidities) exist but were excluded from the current analysis due to possible confounding variables. While many of our findings regarding techniques to ensure rigor in biological models apply generally, the specific turnover conclusions are beholden to the quality and completeness of the input data. Furthermore, our analysis discovered turnover ranges compatible with both datasets but does not formally exclude all other possible turnover scenarios, for which future data will be required.

In conclusion, both the Kajstura and Bergmann studies rely heavily on mathematical models with inherent assumptions in both the model structure and the parameter precision. Our analysis highlights the need to explore these assumptions under various axiomatic regimes to evaluate their impact on the final results. When such considerations are applied to these two estimates of adult human cardiomyogenesis, we find a reasonable mechanism (“subject-age”-dependent CM death rates) capable of reconciling the apparent discrepancies of the turnover estimates. Recognizing the ongoing need to investigate assumptions in mathematical models of turnover, especially as we examine datasets derived from diseased-hearts, we have provided our full Java program (Filename: Code S1) and illustrative spreadsheets for the TVB-TVDR model in (Spreadsheet S1) to facilitate such exploration, with coding options present to test various inherent premises.

## Methods

### Modeling Approach

A JAVA-based mathematical model heart was developed in which a modeled subject's end-of-life CM age distributions, defined by the modeled subject's lifespan and CM turnover dynamics could be computed. These age distributions were produced by initializing the model with a starting number of CMs and then iteratively (with one iteration per year in the subject lifespan) forming new CM and destroying existing CM in accordance with formation/destruction parameters either specified with direct numeric rates or computed from component parameters such as those enumerated in Figure 1. In some model simulations, apoptotic elimination was formulated to selectively target CMs based on subject age or CM age (iteration of CM formation within the progression of iterations) whereas other model regimes assumed random CM elimination.

When a timeline of atmospheric C14 was included as a reference input and the model heart was assigned a finite birth (start) year on that timeline, each CM formed in the simulation was assigned a DNA C14 value roughly equal to the appropriate atmospheric C14 level at the time of CM formation but adjusted for polyploidization. Polyploidization adjustment was computed by, for each CM age group in the simulation, beginning with the atmospheric C14 level at time of CM age group formation and iteratively (for each year between from formation until subject death) replacing that C14 number with a weighted average of the number (weighted by 100%) and the atmospheric C14 level contemporary to the next iteration's polyploidization event (weighted by the percentage of CM undergoing polyploidization at that stage in the subject's life, as specified by the Adler/Bergmann sigmoidal polyploidization curve).

Under these conditions, each CM surviving to the end of the simulation contributed DNA C14 to an average CM DNA C14 value for the modeled subject. Subtracting the initial atmospheric C14 level (at the start of simulation) from this final C14 weighted average yielded a ΛC14 value associated with the modeled subject.

In some analyses, the inverse operation was performed—converting an experimentally measured ΛC14 value (or a simulated ΛC14 value) attributed to a subject, into a best fit turnover solution—the turnover dynamics that best produce the observed or target ΛC14 value. For this task, a numerical solver evaluated a wide range of turnover solutions (constant or time-varying depending on specified restrictions) by generating a time-of-death CM age distribution resulting from each potential turnover solution in the testable range. Then, for each CM age group in each hypothetical distribution, the C14 contributed by a CM in the age group was computed (with polyploidization correction as aforementioned. Multiplying the fractional representation of each CM age group by the polyploidization-adjusted C14 contribution of that age group yields a net ΛC14 value for the potential turnover solution. The turnover dynamics yielding the closest fit to the measured or target ΛC14 is selected as the best fit solution.

### Sources of Input Parameters

Age-dependent, gender-dependent parameter values for the number of CSCs, fraction of such CSCs cycling at any given moment, number of CSC divisions prior to senescence, and fraction of CM undergoing apoptosis at any given moment were extracted from the Kajstura manuscript. Thus for any modeled subject of any age or gender, a single value for each of these parameters (derived from regression models of 74 subjects computed in the Kajstura document) could be referenced and incorporated into a dynamic model heart. The formula for single-iteration CM count change (Figure 1) was also obtained from the Kajstura manuscript.

Similarly, raw data regarding subject-specific year of birth, year of death, and CM DNA C14 levels for each of 12 subjects studied in the Bergmann manuscript were extracted. These CM DNA C14 levels were reported by Bergmann as differential C14 measurements (measured C14 relative to 1955, prior to the rise in atmospheric C14. These differential measurements were converted to ΛC14 values by subtraction from the initial C14 levels (also differentials from 1955 values) for use in the hybrid model. The polyploidization age progression formula was also obtained from the Bergmann manuscript.

### Examination of Kajstura Manuscript in Isolation Using Hybrid Model

The Kajstura manuscript proposes two complementary modeling methods—a hierarchically-structured cell kinetics model (controlled by CSC cycling rates and CM apoptosis rates) and an age-structured cell kinetics model controlled by CM half-life and apoptosis rates). In the hierchically-structured model, an initial cell population of 500 Million CM/10 g tissue was used to start the simulation. Then, for each year in the simulation, CM were iteratively created or destroyed based on the assumed modeling parameters (enumerated in Figure 1). For the age-structured model, CM age distributions at time of subject death were produced for simulated subjects (defined by age at time of death and gender) according to the formula utilized by Kajstura et al, a modified version of the McKendrick von Foerster population dynamics formula:
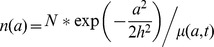
where n(a) is the number of CM of a given age present at time of autopsy (for 10 g tissue), N is the initial number of CM present in 10 g tissue, a is the age of the CM, h is the halflife of a CM, and µ(a,t) is a mortality function dependent on a and the subject age t. In the hybrid model, µ(a,t) is realized by iteratively computing for all t (0 to lifespan) the number of CM destroyed (given as “per million” values by Kajstura measurements) and deducting the destroyed CM from the then-current total.

### Examination of the Bergmann Manuscript in Isolation Using Hybrid Model

The ability of the hybrid model to faithfully emulate the Bergmann methodology for computation of turnover levels from ΛC14 data was measured by substituting the hybrid model inputs with the single turnover value concluded by Bergmann in the Bergmann document for each of the 12 Bergmann subjects (modeled hearts with birth date and lifespan equivalent to that of a Bergmann subject). After integrating atmospheric C14, polyploidization data, and turnover derivation methodology from the Bergmann document, closeness of hybrid model output turnover to input turnover was observed, allowing quantification of distortion caused by the hybrid modeling procedure.

To determine inherent sensitivity of the Bergmann algorithm, a wide range of input turnovers was supplied to the hybrid model (as substitution for CM formation/destruction parameters) and output turnover for each input was reported. The impact of polyploidization correction on turnover estimations for the 12 Bergmann subjects was performed by conducting simulations with scaled fractions of subject-specific polyploidization correction factors measured in the Bergmann document and reporting the outputted turnover estimation. The impact of a delay in incorporation of atmospheric C14 into human tissue was performed by running the hybrid model with the bomb curve moved forward in time by the proposed delay.

Effect of initial C14 values on the hybrid model was performed by running the hybrid model with initial C14 levels (generally obtained from 1-year smoothing of atmospheric C14 readings obtained by Levin^7,8^) with the minimal or maximal readings obtained during that 1-year period.

### Finding Turnover Models Simultaneously Compatible with Bergmann and Kajstura Datasets

A global fit solver was produced to identify parameter combinations most consistent with given ΛC14 result for a given modeled subject heart. To test the global fit solver's equivalence to the Bergmann global fit solver, model regimes utilized by Bergmann were subjected to the hybrid model global fit solver and optimum parameter selection and mean squared error were compared to those reported by Bergmann.

In one of the Bergmann models (denoted E2 by Bergmann) the optimum parameters γ_0_ and γ_1_ were disregarded by Bergmann and alternative values were chosen to maximize differentiation from Scenario A. Although the parameter boundaries used by Bergmann are not stated, we find the best fit parameters for this model to be γ_0_ = 0.45, γ_1_ = 0.4, with a SSE of 29,793 (AIC = 3.57) when ranges are γ_0_:{0, 0.5} at increments of 0.01 and γ_1_:{0,2} at increments of 0.05; these values correspond to a youthful turnover rate of approximately 5.5% declining to 4% in old age. However, in the analysis of the E2 model, the parameters selected by Bergmann are used.

Alternative model schema (including the Time-Varying Birth Rate, Time Varying-Death Rate or “TVB-TVDR” model), inspired by the Kajstura conclusion of subject age (time)-increasing turnover with subject age (time)-increasing vulnerability of newly formed CMs to apoptosis, were evaluated for quality of fit with the Bergmann ΛC14 measurements in the 12 subject dataset from the Bergmann document (note: the parameter sweep included negative, constant, and positive temporal dependence). Though the concept of subject age-dependent inferiority of newly-created CMs is conceptually derived from the Kajstura findings, single age-dependent CM formation/death rates were used in this model and thus no formulas, parameters, values, or assumptions from the Kajstura manuscript were used in this exercise.

Ranges of values for the TVB-TVDR (with fully independent variables) were as follows: B_s_:{−1.0%/year^2^, +1.0%/year^2^}, B_i_:{0%/year, 10%/year}, D_s_:{−1.0%/year^2^, +1.0%/year^2^}, D_i_:{0%/year, 10%/year} with slopes (B_s_, D_s_) tested at increments of 0.05%/year^2^ and intercepts (B_i_, D_i_) tested at increments of 0.1%/year).

### Hybrid Model Integration of Kajstura and Bergmann Models

The 12 Bergmann subjects were modeled (i.e. models were created using birth date and lifespans equivalent to the Bergmann subject set) with input turnover dynamics as prescribed by Kajstura or with turnover dynamics with best global fits to the Bergmann data (as identified by the global solver) to produce CM age histograms at time of modeled subject death. Expected ΛC14 levels corresponding to each of these CM age histograms were then produced for comparison to the ΛC14 observations made by Bergmann for these subjects.

Expanded methods including JAVA code routine titles are found in the methods (Methods S1)

## Supporting Information

Figure S1 **Input Atmospheric C14 Levels from 1930–2007.** In accordance with the Bergmann approach, C14 measurements were extracted from the Levin datasets (Europe from 1959–2003, and 2003–2007) and scaled to the Bergmann unit system. Years prior to 1955 were estimated as null (as no human nuclear activity occurred during this time). For modeling purposes, a 1 year smoothing function was applied, consistent with the Bergmann approach.
(TIF)

Figure S2 **The Hybrid Model Successfully Reproduces Kajstura Cell Dynamics** **Results** **from Kajstura Input Parameters.** A) If cardiomyogenesis is removed from the model, the Kajstura CM count trajectories for male and female subjects decay identically with the trajectories reported in the Kajstura manuscript. B) Hybrid model myocyte formation for both male and females is identical to the temporal trend reported in the Kajstura manuscript. C) Hybrid model CM turnover for both male and females is identical to the temporal trend reported in the Kajstura manuscript. Collectively, these results indicate that the hybrid model captures the Kajstura model dynamics and is functioning as intended.
(TIF)

Figure S3 **The Hybrid Model Successfully Reproduces Kajstura CM Age Distribution.** Male and female distributions for young, middle-aged, and old subjects were extracted from the Kajstura manuscript via a pixel-counting method. The hybrid model produced age distributions for these age groups for both genders. The hybrid models are highly overlapping with the reported Kajstura results and are nearly identical in average CM age for the various age groups for both genders. The trend towards sharper, younger distributions with advancing age is captured by the hybrid model for both genders. These results indicate that the hybrid model is a reliable mechanism for modeling subjects using the Kajstura parameters.
(TIF)

Figure S4 **Sensitivity of the Kajstura Analysis Estimate of CM Turnover to the Expansion Exponent Variable.** The Expansion Exponent is the number of divisions that a Cardiac Stem Cell is expected to undergo before becoming senescent (non-replicative). The hybrid model was tested with the value of the exponent (which changes with subject age and is gender-specific) decreased by 20% (E = 80%) or increased by 20% (E = 120%). Acute sensitivity to the exponent is shown as a 20% variation in E results in a 2-fold change in turnover for A) Male and B) Female.
(TIF)

Figure S5 **Kajstura CM Count Trajectories and Sensitivity to Input Variables.** The number of CM in the heart is assumed to be 500 Million/10 gram tissue at age 20 (reported in the Kajstura manuscript). The hybrid model is applied for male and female hearts while one variable is either at 80%, 100%, or 120% of the value reported in the Kajstura manuscript. A) The duration of apoptosis is varied (100% = 4 hours, based on literature maximum values for other cell types). B) The duration of mitosis is varied (100% = 26 hours, in vitro cycling of CSCs observed by Kajstura). C) The expansion exponent—number of CSC divisions prior to loss of replicative ability—is varied. CM count change with age (a function of CM formation and apoptotic destruction) is most sensitive to the Expansion Exponent. Under parameters reported by Kajstura (E = 100%), modeled female hearts contain more CM per 10 grams in middle-age than at youth. Reduction of the Expansion Exponent by 20% produces a monotonically decreasing CM count trajectory for females.
(TIF)

Figure S6 **Sensitivity of CM Age Histograms for Young, Middle-Aged, and Old Subjects to Input Variables.** The hybrid model was run with either A) apoptosis cycle duration at 80%, 100% (4 hours), or 120% of the value used in the Kajstura manuscript or B) CM half life at 80%, 100%, or 120% (function of subject age) of the value used in the Kajstura manuscript. 80% values are indicated in red. 100% values are indicated in black. 120% values are indicated in green. For both male and female modeled hearts, and for all age groups, decreasing apoptosis duration resulted in slightly younger CM age distributions, while increasing apoptosis resulted in slightly older CM age distributions. Age distributions were more sensitive to half life (which indirectly incorporates apoptosis) and, for all age groups and both genders, decreasing half life resulted in younger distributions while increasing half life resulted in older distributions. The effect of half life was most noticeable on younger model hearts.
(TIF)

Figure S7 **Kajstura Turnover with Alternative Apoptosis Parameters.** The hybrid model was parameterized in accordance with the Kajstura published values for Cardiac Stem Cell number, cycling frequency, mitosis duration, and expansion exponenent; as well as either the Kajstura apoptosis parameters (Red) or from an alternative estimation by Mallet (blue). To include apoptosis (and thus, non-constant CM heart content) in the computation of CM turnover, turnover for a given year was computed as “newly formed CM in that year divided the CM density at that year as determined by preceding iterative production and destruction of CM,” rather than as “newly formed CM in year divided by a constant 5,000,000 CM/10 g tissue” as was done in the Kajstura manuscript. The simulation is performed for A) Males and B) Females
(TIF)

Figure S8 **Kajstura Model Turnover Conclusions under Various Apoptotic CM Fractions.** The hybrid model was parameterized in accordance with the Kajstura published values for Cardiac Stem Cell number, cycling frequency, mitosis duration, and expansion exponent; as well as either the Kajstura apoptotic CM fraction as a function of time (A = 1X) or some multiple of that parameter (A = 0X, 0.25X, 0.5X, 1.5X, 2X). To include apoptosis (and thus, non-constant CM heart content) in the computation of CM turnover, turnover for a given year was computed as “newly formed CM in that year divided the CM density at that year as determined by preceding iterative production and destruction of CM.” The simulation is performed for A) Males and B) Females
(TIF)

Figure S9 **Hybrid Model Validation Strategy.** To demonstrate hybrid model fidelity to the Bergmann system, the hybrid model CM formation/destruction inputs were substituted with the constant turnover levels concluded by Bergmann. When the hybrid model's polyploidization function is disabled, the hybrid model computed ΛC14 levels expected to be produced by such stipulated turnover (these values may be compared to the non-polyploidization-corrected values, i.e. raw measured values obtained by Bergmann [Bergmann Fig. 3A/3B] for closeness). Similarly, when the hybrid model's polyploidization function was enabled, the hybrid model computed ΛC14 levels expected to be produced by such stipulated turnover (these may be compared for closeness to Bergmann's post-ploidy corrected ΛC14 values [Bergmann Fig. 3D] for closeness). If both tests report similar C14 values to those reported by Bergmann, than the model introduces no unwanted distortions in the development of CM age histograms or in computing C14 values from them. Also, if both tests conform to Bergmann, than the hybrid model compensates for polyploidization identically to Bergmann. Furthermore, non-polyploidization-corrected C14 values can be fed into the hybrid model's numerical solver (which has a polyploidization correction module equivalent to that used to test the aforementioned points), and the resulting turnover conclusions can be compared with the Bergmann concluded turnover results to validate that no distortions are caused by the numerical solver.
(TIF)

Figure S10 **Hybrid Model Validation** **Results** **.** The validation strategy described in Figure S9 is applied. When the Bergmann final concluded turnover levels were used as the hybrid model input, the hybrid model created simulated hearts for each subject and estimated final average C14 content based on the age histograms produced for each subject under these turnover conditions. A) The raw C14 measurement produced matched those in the Bergmann manuscript. B) The hybrid model then computed C14 attributable to poly-ploidization, which again matched the Bergmann conclusions. C) After compensating for poly-ploidization, the Hybrid Model's numerical solver converted C14 values into annual turnovers which matched the initial parameterization values.
(TIF)

Figure S11 **Cardiomyocyte Turnover Estimates using Bergmann Approach and Dataset with Bergmann Various Poly-ploidization Correction Factors.** A) Takamatsu et al concluded a poly-ploidization level approximately equal to 78% of the level concluded by Bergmann with nearly identical age progression. Substituting a poly-ploidization correction factor based on the Takamatsu conclusion to the Bergmann ΛC14 dataset yields modestly higher estimates of turnover that concluded by Bergmann for subjects born after the rise in atmospheric C14 (1.3%±0.2% by Takamatsu versus 1.6%±0.1% by Bergmann, p<0.15). The substitution of Takamatsu correction factor has no impact on the 4 modeled subjects born prior to 1950 such that poly-ploidization largely completes prior to C14 atmospheric rise (0.18%±0.08% by Takamatsu versus 0.15%±0.09%). B) A wide range of poly-ploidization magnitudes (with unchanged age relationship) are applied to the hybrid modeled subjects. A scaling of 100% is equivalent to the Bergmann values used by Bergmann, whereas 200% is equivalent to twice the level of ploidization and 0% indicates an assumption that poly-ploidization does not exist. In general, there is a negative linear relationship between concluded turnover and poly-ploidization correction. Oldest 3 subjects are insensitive to variance in polyploidy and are not shown.
(TIF)

Figure S12 **Hybrid Model Simulations for the Bergmann Subject Dataset under Various Initial C14 Incorporation Assumptions.** A) The hybrid model was parameterized with turnover levels as concluded by Bergmann to generate an assumed CM age histogram at time of autopsy. The hybrid model then determined an associated ΛC14 for each surviving CM in each modeled subject under the assumption that atmospheric C14 is either instantly incorporated into newly formed CM DNA or that there is a 2-year delay (such that CM DNA C14 concentrations are equal to the atmospheric concentration 2 years prior to CM formation). Subject ΛC14 levels were then computed and the numerical solver was used to derive corresponding turnover levels (assuming constant turnover). B) The hybrid model was parameterized with turnover levels as concluded by Bergmann to generate an assumed CM age histogram at time of autopsy. The hybrid model then determined an associated ΛC14 for each surviving CM in each modeled subject under the assumption that atmospheric C14 is instantly incorporated into newly formed CM DNA. However, initial C14 levels (which determine the C14 content of initial CMs, comprising the bulk of CMs present at time of autopsy in low turnover models), were assigned C14 levels equal to either (1) the 1-year smoothed atmospheric C14 level at time of subject birth, or (2) the lowest C14 level measured by Levin within 1 year of subject birth, or (3) the highest C14 level measured by Levin within 1 year of subject birth. Subject ΛC14 levels were then computed and the numerical solver was used to derive corresponding turnover levels (assuming constant turnover). The turnover levels derived from smoothed C14 measurements are shown as blue bars, with the turnover levels produced by using minimal and maximal atmospheric C14 levels as initial C14 levels are shown as error bars.
(TIF)

Figure S13 **Performance of Various Model Scenarios.** A) Representative CM age histogram for oldest Bergmann subject (ND70) under Kajstura, Bergmann Scenario E2, Bergmann Scenario A, and TVB-TVDR best-fit models. Age distribution is bimodal in the Best Global Fit TVB-TVDR model with a cohort of original CM persisting until death, bolstered by low death rate and high initial representation, and young CM produced due to high rates and having undergone few annual death cycles. B) Representative CM age histogram for youngest subject (ND74). C) Expected end C14 (time of autopsy) measurements for the various scenarios (Bergmann measured values supplied for reference). The TVB-TVDR model, by selective depletion of intermediate CMs, fits both older and younger subjects well despite having a comparatively high (with regards to the Bergmann conclusions) turnover of 4.5% increasing to 15% by age 70. The Bergmann models (E2 and A) capture the general temporal pattern but shows substantial deviations numerically; particularly for the oldest 3 subjects which are the lynchpin of the low turnover hypothesis. The Kajstura turnover actually fits older subjects better than the constant turnover scenario due to the elimination of CM produced during the highest atmospheric C14 concentrations, but fails to match younger subjects as the high turnover drives all Kajstura predicted C14 levels to peri-mortem levels. Error bars represent the simulated end C14 concentrations for each subject when either the lowest or highest initial atmospheric C14 (within 1 year of time of subject birth) levels are used (except for the Kajstura model, which has no sensitivity to initial C14 concentration due to obliteration of nearly all initial CMs; for this model set, the primary variable “CM Half Life” is varied to 80% or 120% of values concluded by Kajstura). *Notably, the best fit model (age-increasing CM formation) would increase total CM in adolescence to a peak of 130% of initial count (birth) by age 20, which would then decrease to 87% of count at birth by year 70 (not shown).
(TIF)

Figure S14 **Sensitivity of the Kajstura Analysis Estimate of CM Turnover to the Apoptosis Fraction Variable.** The Apoptosis Fraction is the measured percent of CM observed to be undergoing apoptosis at any given point in time. The hybrid model, under the adjusting turnover definition, was tested with the value of the fraction (which changes with subject age and is gender-specific) decreased by 20% (A = 80%) or increased by 20% (A = 120%). Sensitivity to the parameter A seems to be greatest when considering subjects of advanced age. Simulations were run for A) Male and B) Female.
(TIF)

Figure S15 **Sensitivity of the Kajstura Analysis Estimate of CM Turnover to the Expansion Exponent Variable.** The Expansion Exponent is the number of divisions that a Cardiac Stem Cell is expected to undergo before becoming senescent (non-replicative). The hybrid model, under the adjusting turnover definition, was tested with the value of the fraction (which changes with subject age and is gender-specific) decreased by 20% (E = 80%) or increased by 20% (E = 120%). Sensitivity to the parameter E seems to be greatest when considering subjects of youthful age whereas in advanced age, changes in CM formation are roughly compensate for by changes in cumulative cell count. Simulations were run for A) Male and B) Female.
(TIF)

Table S1 **Modeled Subject Genders, Birth Years, and Lifespans.** Modeled subject input parameters were extracted from the Bergmann study.
(TIF)

Spreadsheet S1 (XLSX)

Code S1 (DOCX)

Methods S1 (DOCX)
