# Supplementary figures and images for: Hybrid Mathematical Model of Cardiomyocyte Turnover in the Adult Human Heart
Source: PLoS One. 2012 Dec 19;7(12):e51683. doi: 10.1371/journal.pone.0051683 (PMC3526650; doi:10.1371/journal.pone.0051683)

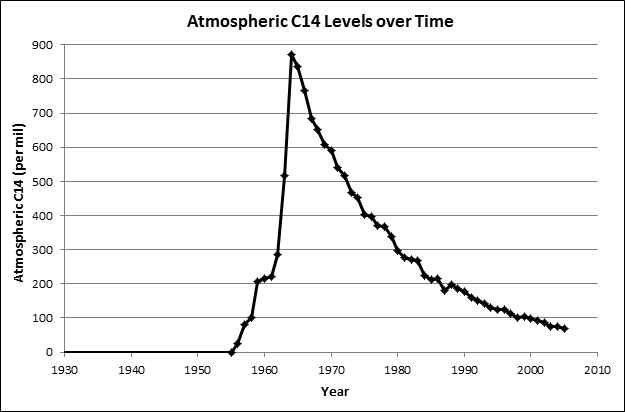

Supplement: Figure S1 — Input Atmospheric C14 Levels from 1930–2007. In accordance with the Bergmann approach, C14 measurements were extracted from the Levin datasets (Europe from 1959–2003, and 2003–2007) and scaled to the Bergmann unit system. Years prior to 1955 were estimated as null (as no human nuclear activity occurred during this time). For modeling purposes, a 1 year smoothing function was applied, consistent with the Bergmann approach. (TIF) [file pone.0051683.s001.tif]

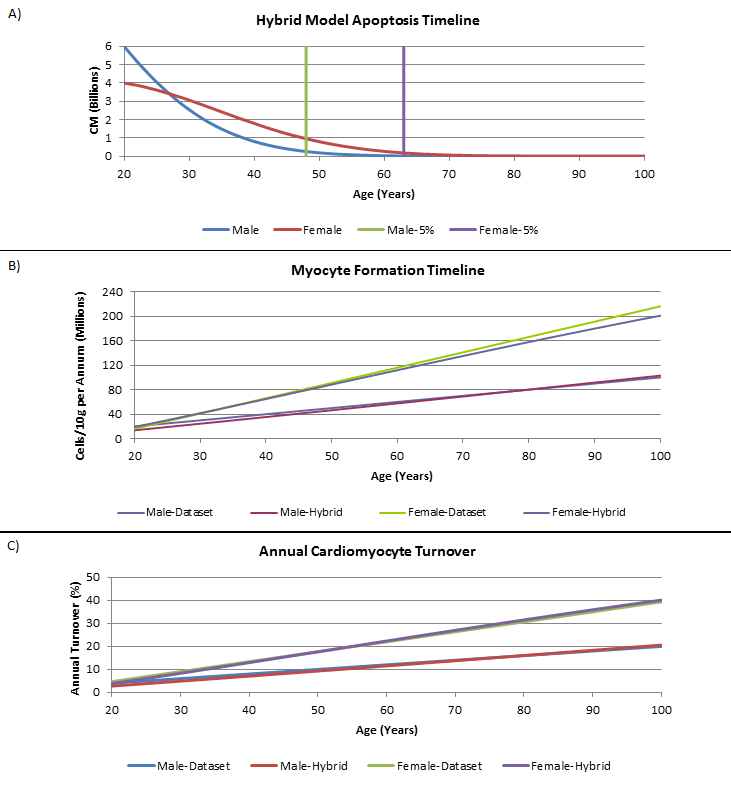

Supplement: Figure S2 — The Hybrid Model Successfully Reproduces Kajstura Cell Dynamics Results from Kajstura Input Parameters. A) If cardiomyogenesis is removed from the model, the Kajstura CM count trajectories for male and female subjects decay identically with the trajectories reported in the Kajstura manuscript. B) Hybrid model myocyte formation for both male and females is identical to the temporal trend reported in the Kajstura manuscript. C) Hybrid model CM turnover for both male and females is identical to the temporal trend reported in the Kajstura manuscript. Collectively, these results indicate that the hybrid model captures the Kajstura model dynamics and is functioning as intended. (TIF) [file pone.0051683.s002.tif]

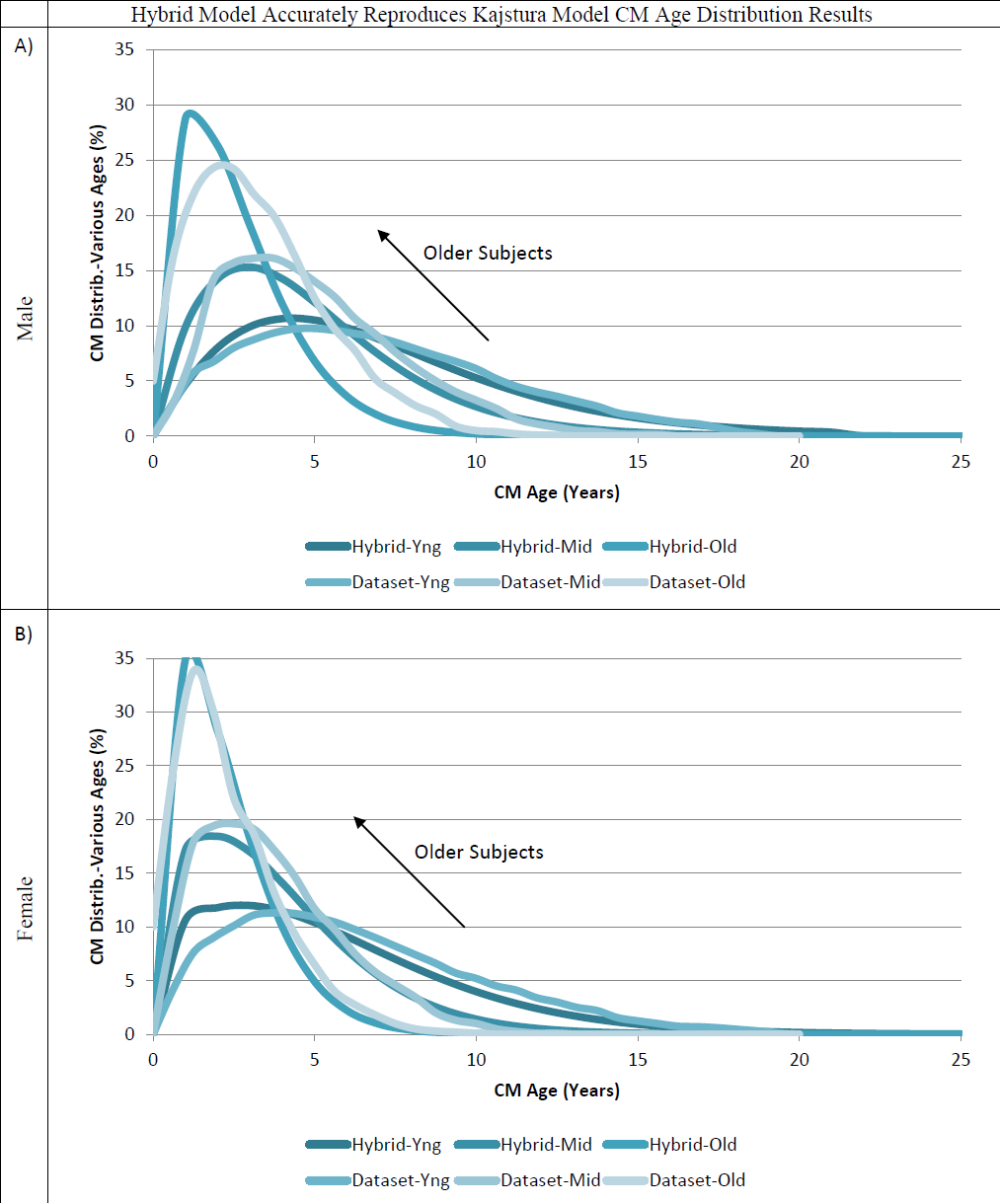

Supplement: Figure S3 — The Hybrid Model Successfully Reproduces Kajstura CM Age Distribution. Male and female distributions for young, middle-aged, and old subjects were extracted from the Kajstura manuscript via a pixel-counting method. The hybrid model produced age distributions for these age groups for both genders. The hybrid models are highly overlapping with the reported Kajstura results and are nearly identical in average CM age for the various age groups for both genders. The trend towards sharper, younger distributions with advancing age is captured by the hybrid model for both genders. These results indicate that the hybrid model is a reliable mechanism for modeling subjects using the Kajstura parameters. (TIF) [file pone.0051683.s003.tif]

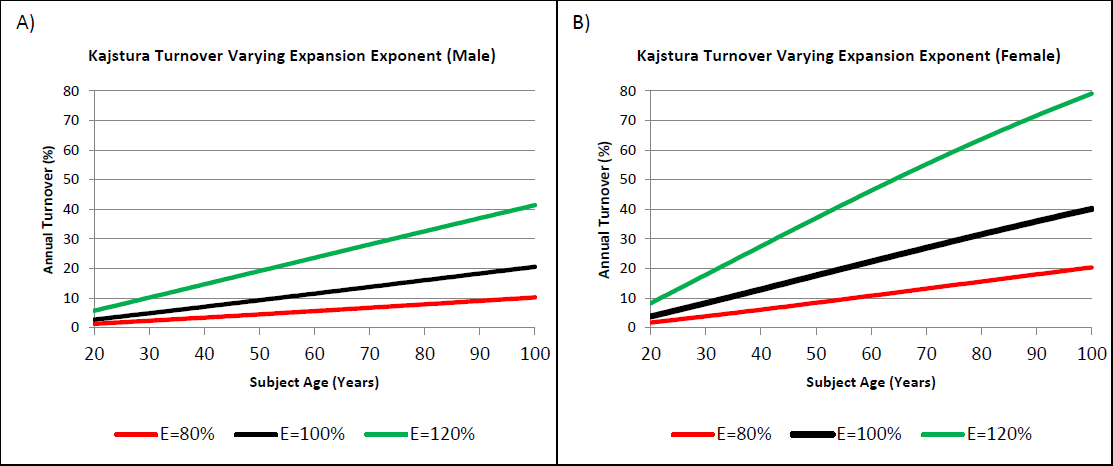

Supplement: Figure S4 — Sensitivity of the Kajstura Analysis Estimate of CM Turnover to the Expansion Exponent Variable. The Expansion Exponent is the number of divisions that a Cardiac Stem Cell is expected to undergo before becoming senescent (non-replicative). The hybrid model was tested with the value of the exponent (which changes with subject age and is gender-specific) decreased by 20% (E = 80%) or increased by 20% (E = 120%). Acute sensitivity to the exponent is shown as a 20% variation in E results in a 2-fold change in turnover for A) Male and B) Female. (TIF) [file pone.0051683.s004.tif]

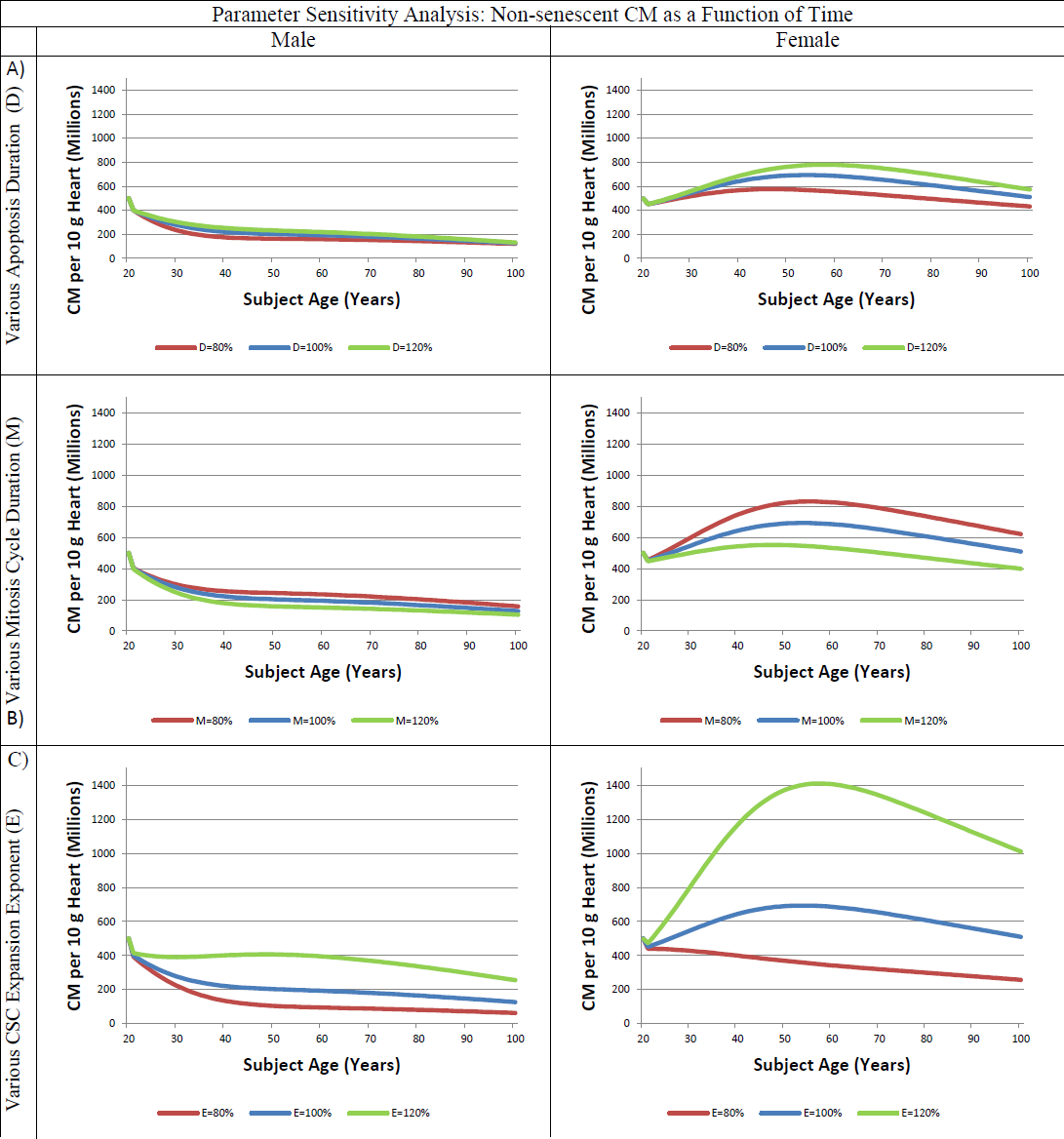

Supplement: Figure S5 — Kajstura CM Count Trajectories and Sensitivity to Input Variables. The number of CM in the heart is assumed to be 500 Million/10 gram tissue at age 20 (reported in the Kajstura manuscript). The hybrid model is applied for male and female hearts while one variable is either at 80%, 100%, or 120% of the value reported in the Kajstura manuscript. A) The duration of apoptosis is varied (100% = 4 hours, based on literature maximum values for other cell types). B) The duration of mitosis is varied (100% = 26 hours, in vitro cycling of CSCs observed by Kajstura). C) The expansion exponent—number of CSC divisions prior to loss of replicative ability—is varied. CM count change with age (a function of CM formation and apoptotic destruction) is most sensitive to the Expansion Exponent. Under parameters reported by Kajstura (E = 100%), modeled female hearts contain more CM per 10 grams in middle-age than at youth. Reduction of the Expansion Exponent by 20% produces a monotonically decreasing CM count trajectory for females. (TIF) [file pone.0051683.s005.tif]

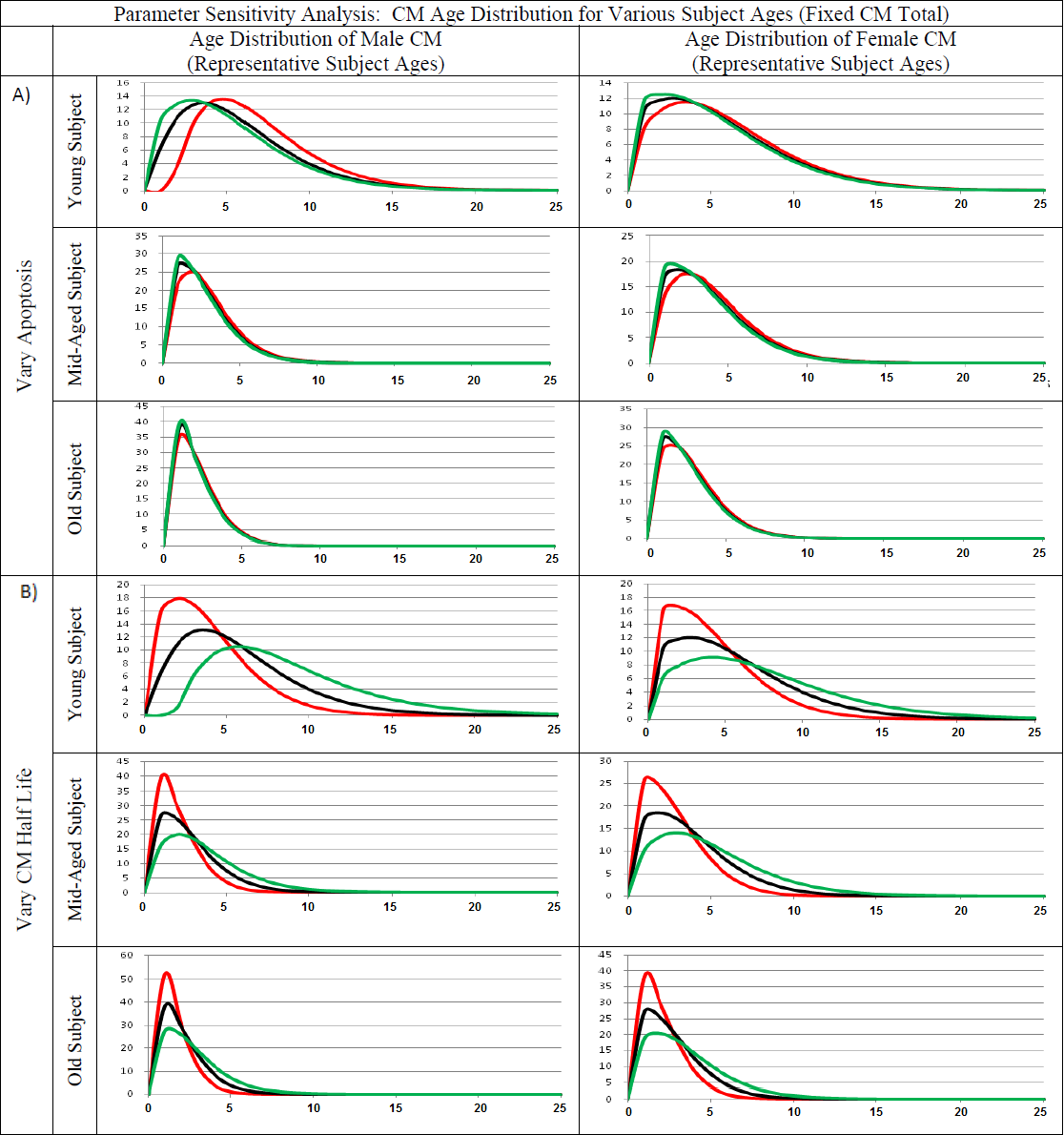

Supplement: Figure S6 — Sensitivity of CM Age Histograms for Young, Middle-Aged, and Old Subjects to Input Variables. The hybrid model was run with either A) apoptosis cycle duration at 80%, 100% (4 hours), or 120% of the value used in the Kajstura manuscript or B) CM half life at 80%, 100%, or 120% (function of subject age) of the value used in the Kajstura manuscript. 80% values are indicated in red. 100% values are indicated in black. 120% values are indicated in green. For both male and female modeled hearts, and for all age groups, decreasing apoptosis duration resulted in slightly younger CM age distributions, while increasing apoptosis resulted in slightly older CM age distributions. Age distributions were more sensitive to half life (which indirectly incorporates apoptosis) and, for all age groups and both genders, decreasing half life resulted in younger distributions while increasing half life resulted in older distributions. The effect of half life was most noticeable on younger model hearts. (TIF) [file pone.0051683.s006.tif]

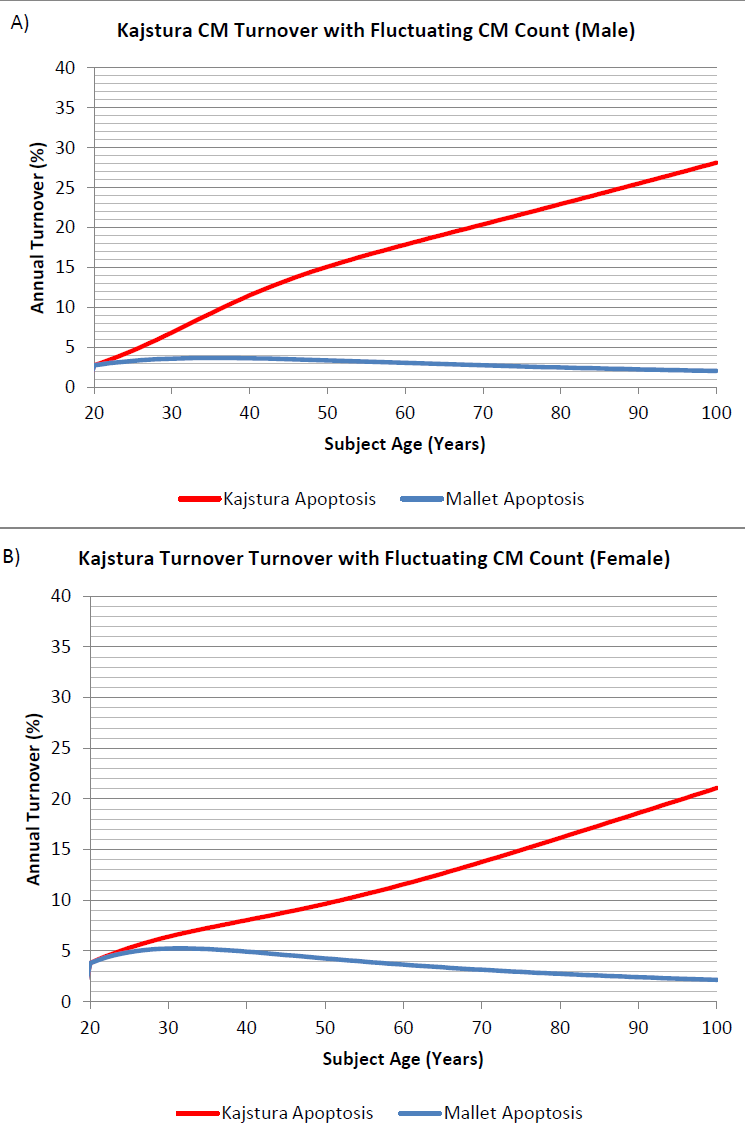

Supplement: Figure S7 — Kajstura Turnover with Alternative Apoptosis Parameters. The hybrid model was parameterized in accordance with the Kajstura published values for Cardiac Stem Cell number, cycling frequency, mitosis duration, and expansion exponenent; as well as either the Kajstura apoptosis parameters (Red) or from an alternative estimation by Mallet (blue). To include apoptosis (and thus, non-constant CM heart content) in the computation of CM turnover, turnover for a given year was computed as “newly formed CM in that year divided the CM density at that year as determined by preceding iterative production and destruction of CM,” rather than as “newly formed CM in year divided by a constant 5,000,000 CM/10 g tissue” as was done in the Kajstura manuscript. The simulation is performed for A) Males and B) Females (TIF) [file pone.0051683.s007.tif]

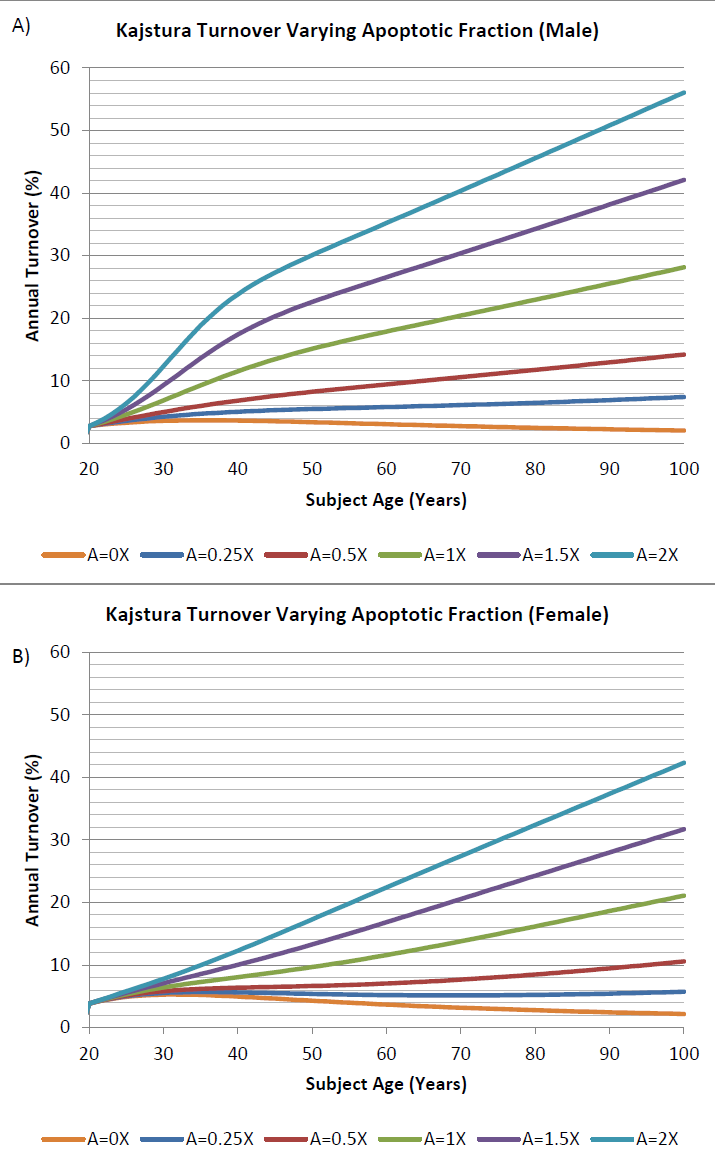

Supplement: Figure S8 — Kajstura Model Turnover Conclusions under Various Apoptotic CM Fractions. The hybrid model was parameterized in accordance with the Kajstura published values for Cardiac Stem Cell number, cycling frequency, mitosis duration, and expansion exponent; as well as either the Kajstura apoptotic CM fraction as a function of time (A = 1X) or some multiple of that parameter (A = 0X, 0.25X, 0.5X, 1.5X, 2X). To include apoptosis (and thus, non-constant CM heart content) in the computation of CM turnover, turnover for a given year was computed as “newly formed CM in that year divided the CM density at that year as determined by preceding iterative production and destruction of CM.” The simulation is performed for A) Males and B) Females (TIF) [file pone.0051683.s008.tif]

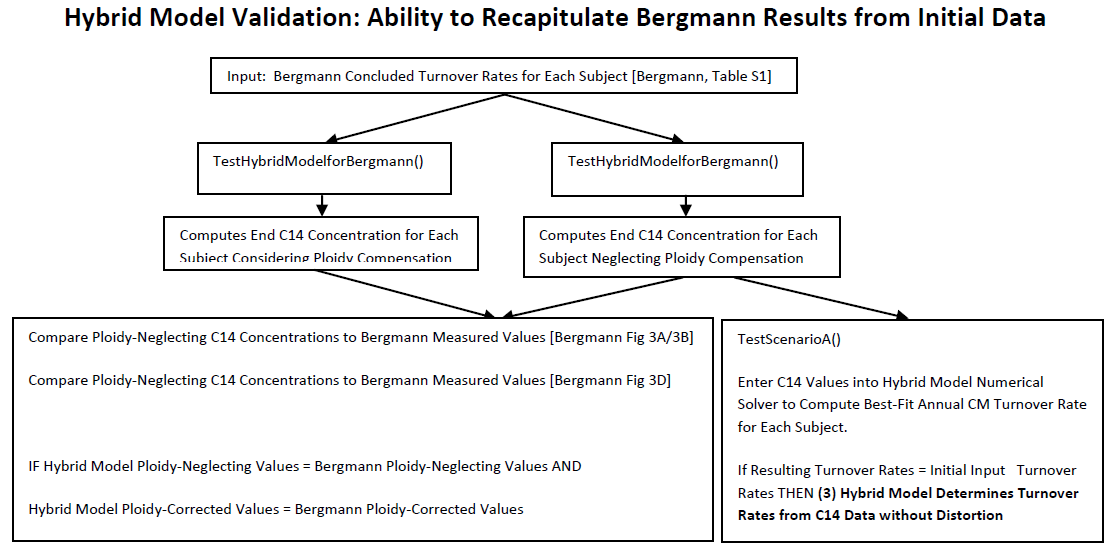

Supplement: Figure S9 — Hybrid Model Validation Strategy. To demonstrate hybrid model fidelity to the Bergmann system, the hybrid model CM formation/destruction inputs were substituted with the constant turnover levels concluded by Bergmann. When the hybrid model's polyploidization function is disabled, the hybrid model computed ΛC14 levels expected to be produced by such stipulated turnover (these values may be compared to the non-polyploidization-corrected values, i.e. raw measured values obtained by Bergmann [Bergmann Fig. 3A/3B] for closeness). Similarly, when the hybrid model's polyploidization function was enabled, the hybrid model computed ΛC14 levels expected to be produced by such stipulated turnover (these may be compared for closeness to Bergmann's post-ploidy corrected ΛC14 values [Bergmann Fig. 3D] for closeness). If both tests report similar C14 values to those reported by Bergmann, than the model introduces no unwanted distortions in the development of CM age histograms or in computing C14 values from them. Also, if both tests conform to Bergmann, than the hybrid model compensates for polyploidization identically to Bergmann. Furthermore, non-polyploidization-corrected C14 values can be fed into the hybrid model's numerical solver (which has a polyploidization correction module equivalent to that used to test the aforementioned points), and the resulting turnover conclusions can be compared with the Bergmann concluded turnover results to validate that no distortions are caused by the numerical solver. (TIF) [file pone.0051683.s009.tif]

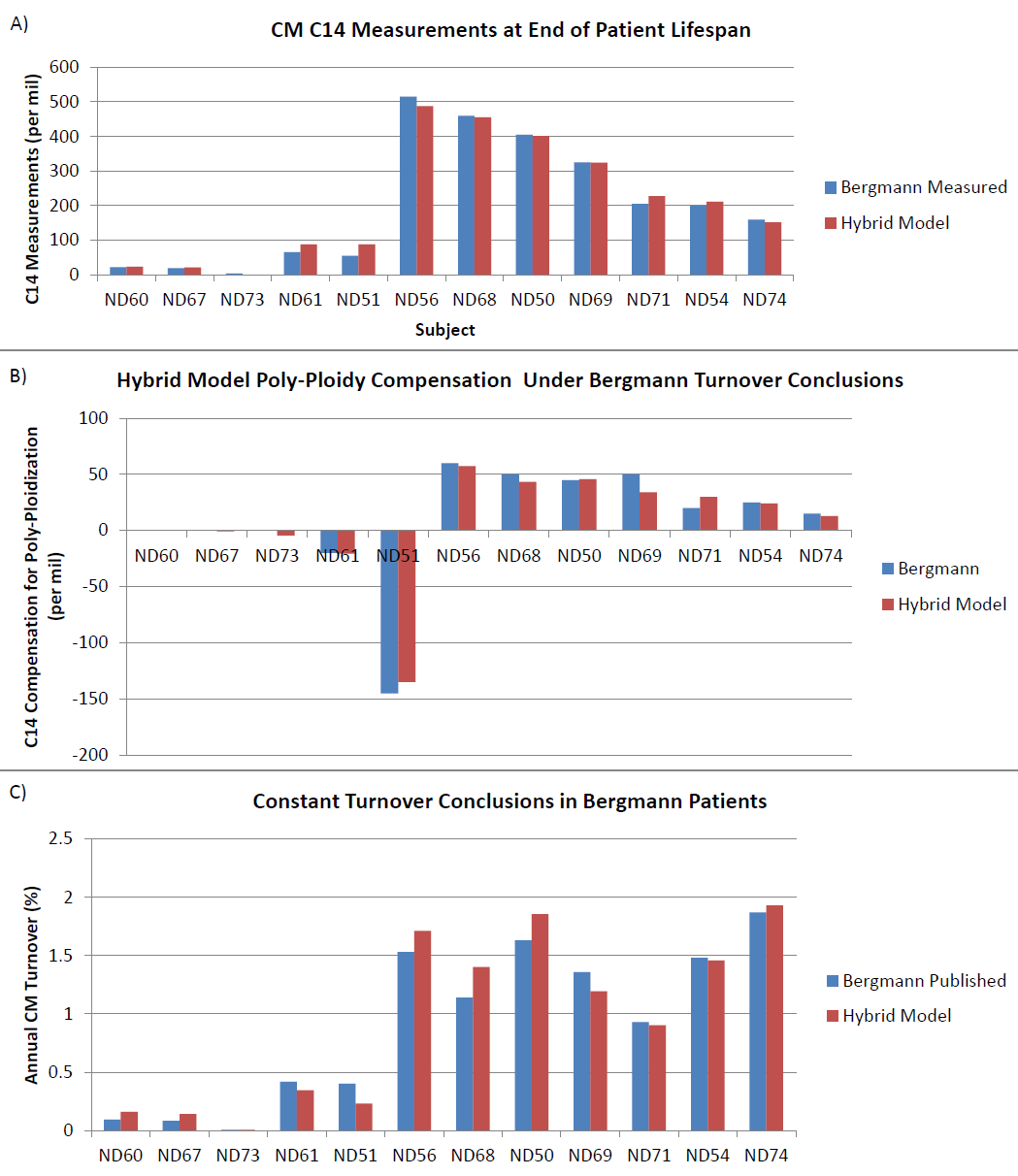

Supplement: Figure S10 — Hybrid Model Validation Results . The validation strategy described in Figure S9 is applied. When the Bergmann final concluded turnover levels were used as the hybrid model input, the hybrid model created simulated hearts for each subject and estimated final average C14 content based on the age histograms produced for each subject under these turnover conditions. A) The raw C14 measurement produced matched those in the Bergmann manuscript. B) The hybrid model then computed C14 attributable to poly-ploidization, which again matched the Bergmann conclusions. C) After compensating for poly-ploidization, the Hybrid Model's numerical solver converted C14 values into annual turnovers which matched the initial parameterization values. (TIF) [file pone.0051683.s010.tif]

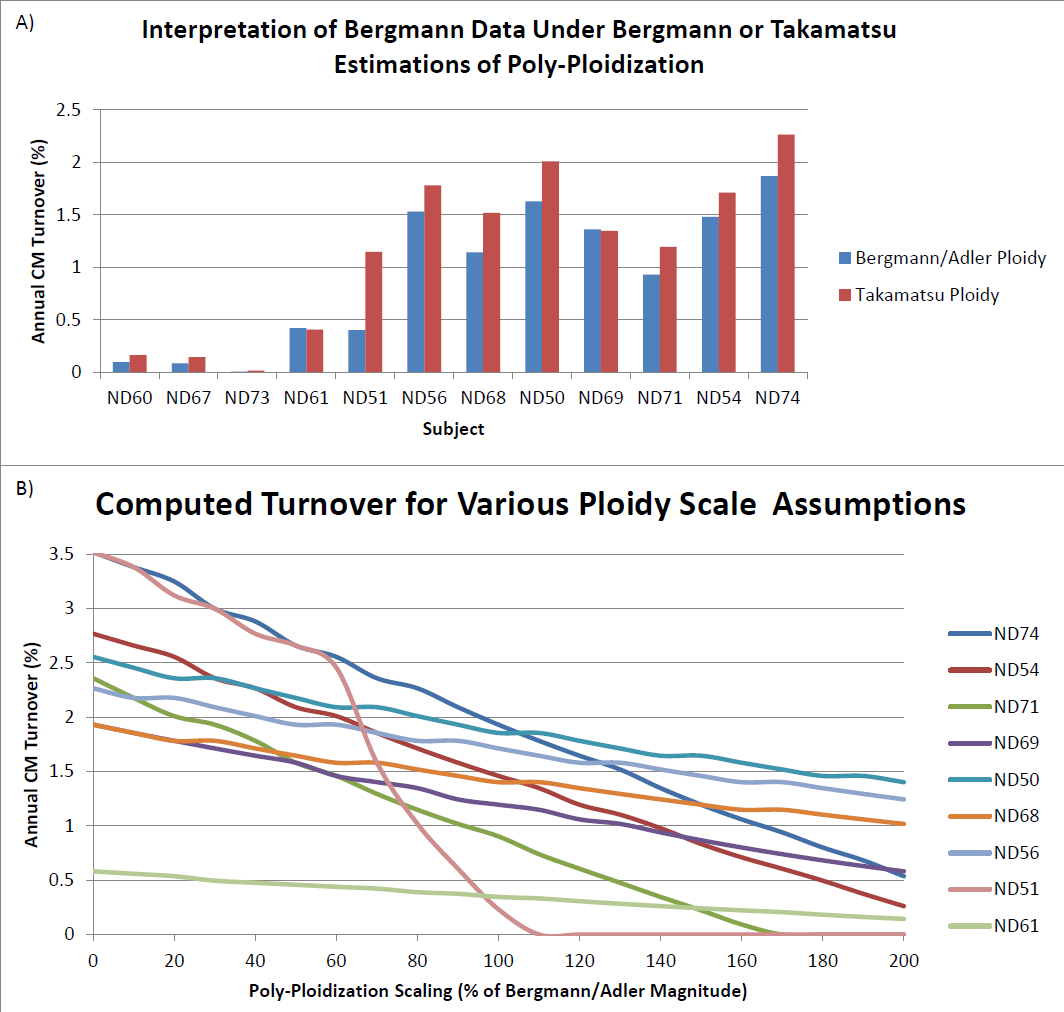

Supplement: Figure S11 — Cardiomyocyte Turnover Estimates using Bergmann Approach and Dataset with Bergmann Various Poly-ploidization Correction Factors. A) Takamatsu et al concluded a poly-ploidization level approximately equal to 78% of the level concluded by Bergmann with nearly identical age progression. Substituting a poly-ploidization correction factor based on the Takamatsu conclusion to the Bergmann ΛC14 dataset yields modestly higher estimates of turnover that concluded by Bergmann for subjects born after the rise in atmospheric C14 (1.3%±0.2% by Takamatsu versus 1.6%±0.1% by Bergmann, p<0.15). The substitution of Takamatsu correction factor has no impact on the 4 modeled subjects born prior to 1950 such that poly-ploidization largely completes prior to C14 atmospheric rise (0.18%±0.08% by Takamatsu versus 0.15%±0.09%). B) A wide range of poly-ploidization magnitudes (with unchanged age relationship) are applied to the hybrid modeled subjects. A scaling of 100% is equivalent to the Bergmann values used by Bergmann, whereas 200% is equivalent to twice the level of ploidization and 0% indicates an assumption that poly-ploidization does not exist. In general, there is a negative linear relationship between concluded turnover and poly-ploidization correction. Oldest 3 subjects are insensitive to variance in polyploidy and are not shown. (TIF) [file pone.0051683.s011.tif]

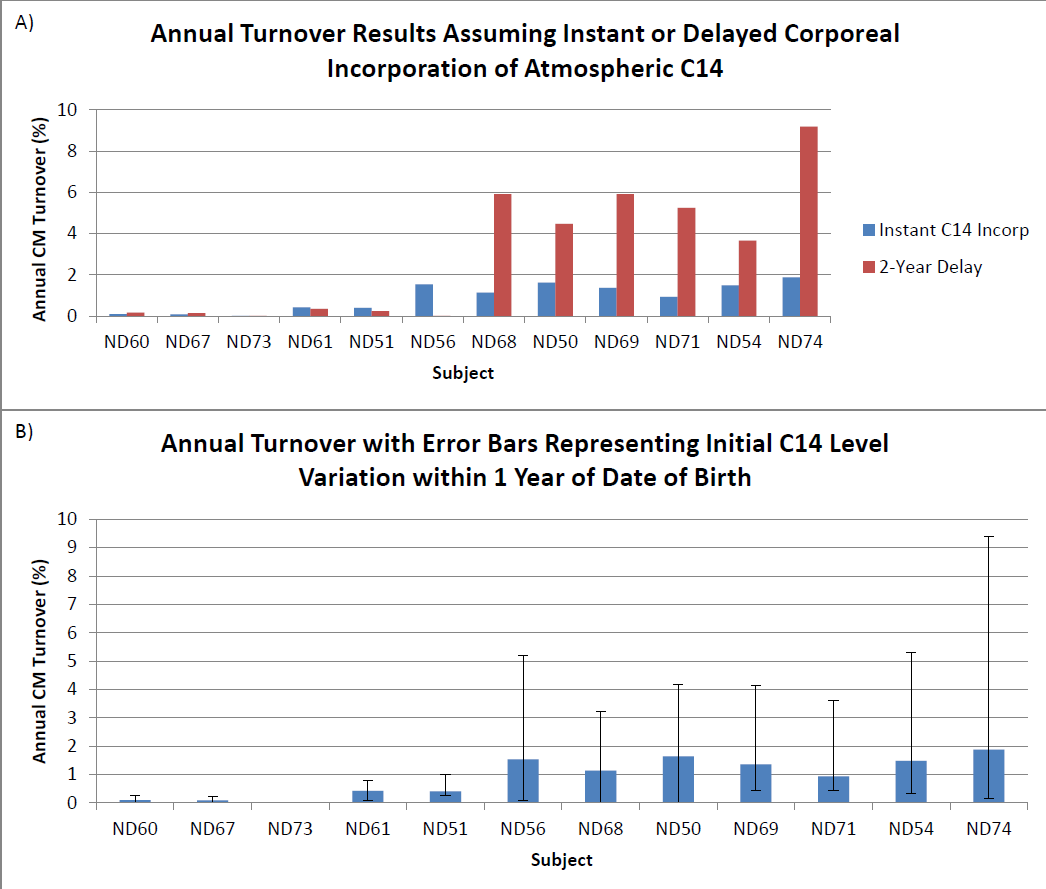

Supplement: Figure S12 — Hybrid Model Simulations for the Bergmann Subject Dataset under Various Initial C14 Incorporation Assumptions. A) The hybrid model was parameterized with turnover levels as concluded by Bergmann to generate an assumed CM age histogram at time of autopsy. The hybrid model then determined an associated ΛC14 for each surviving CM in each modeled subject under the assumption that atmospheric C14 is either instantly incorporated into newly formed CM DNA or that there is a 2-year delay (such that CM DNA C14 concentrations are equal to the atmospheric concentration 2 years prior to CM formation). Subject ΛC14 levels were then computed and the numerical solver was used to derive corresponding turnover levels (assuming constant turnover). B) The hybrid model was parameterized with turnover levels as concluded by Bergmann to generate an assumed CM age histogram at time of autopsy. The hybrid model then determined an associated ΛC14 for each surviving CM in each modeled subject under the assumption that atmospheric C14 is instantly incorporated into newly formed CM DNA. However, initial C14 levels (which determine the C14 content of initial CMs, comprising the bulk of CMs present at time of autopsy in low turnover models), were assigned C14 levels equal to either (1) the 1-year smoothed atmospheric C14 level at time of subject birth, or (2) the lowest C14 level measured by Levin within 1 year of subject birth, or (3) the highest C14 level measured by Levin within 1 year of subject birth. Subject ΛC14 levels were then computed and the numerical solver was used to derive corresponding turnover levels (assuming constant turnover). The turnover levels derived from smoothed C14 measurements are shown as blue bars, with the turnover levels produced by using minimal and maximal atmospheric C14 levels as initial C14 levels are shown as error bars. (TIF) [file pone.0051683.s012.tif]

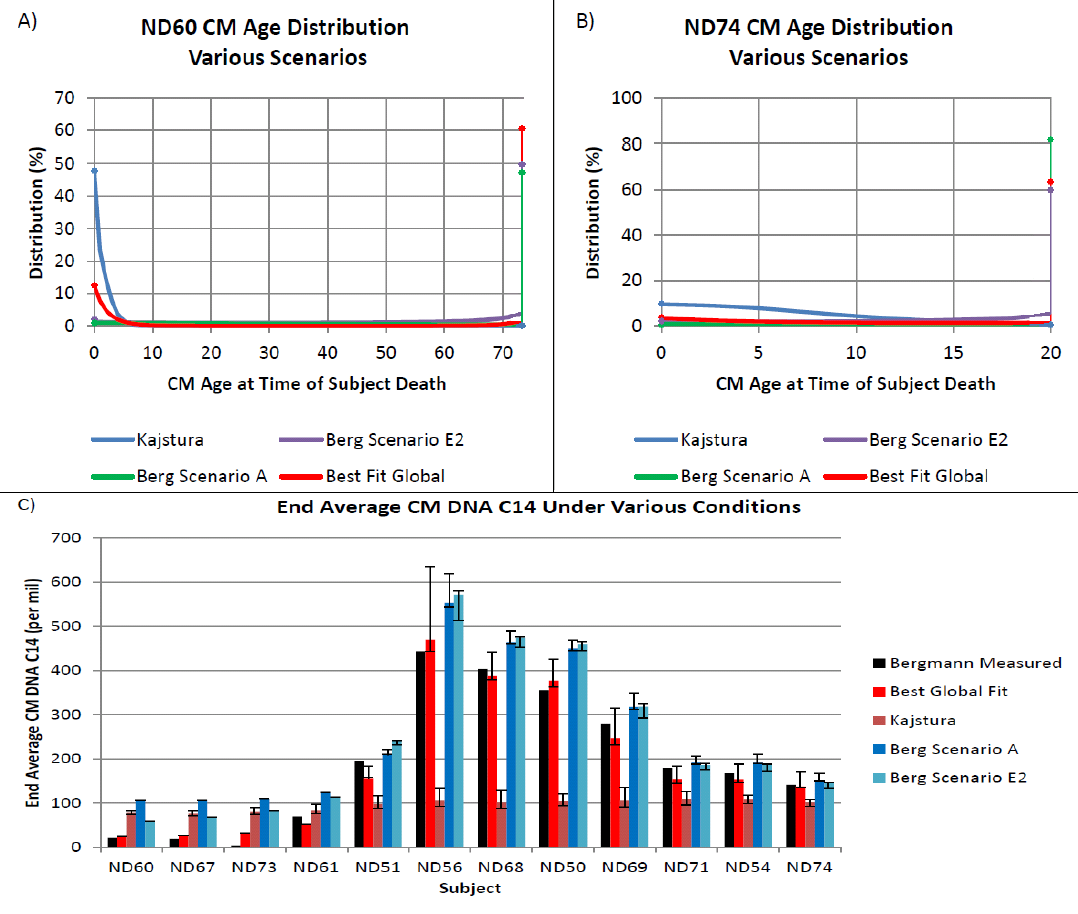

Supplement: Figure S13 — Performance of Various Model Scenarios. A) Representative CM age histogram for oldest Bergmann subject (ND70) under Kajstura, Bergmann Scenario E2, Bergmann Scenario A, and TVB-TVDR best-fit models. Age distribution is bimodal in the Best Global Fit TVB-TVDR model with a cohort of original CM persisting until death, bolstered by low death rate and high initial representation, and young CM produced due to high rates and having undergone few annual death cycles. B) Representative CM age histogram for youngest subject (ND74). C) Expected end C14 (time of autopsy) measurements for the various scenarios (Bergmann measured values supplied for reference). The TVB-TVDR model, by selective depletion of intermediate CMs, fits both older and younger subjects well despite having a comparatively high (with regards to the Bergmann conclusions) turnover of 4.5% increasing to 15% by age 70. The Bergmann models (E2 and A) capture the general temporal pattern but shows substantial deviations numerically; particularly for the oldest 3 subjects which are the lynchpin of the low turnover hypothesis. The Kajstura turnover actually fits older subjects better than the constant turnover scenario due to the elimination of CM produced during the highest atmospheric C14 concentrations, but fails to match younger subjects as the high turnover drives all Kajstura predicted C14 levels to peri-mortem levels. Error bars represent the simulated end C14 concentrations for each subject when either the lowest or highest initial atmospheric C14 (within 1 year of time of subject birth) levels are used (except for the Kajstura model, which has no sensitivity to initial C14 concentration due to obliteration of nearly all initial CMs; for this model set, the primary variable “CM Half Life” is varied to 80% or 120% of values concluded by Kajstura). *Notably, the best fit model (age-increasing CM formation) would increase total CM in adolescence to a peak of 130% of initial count (birth) by age 20, which woul [file pone.0051683.s013.tif]

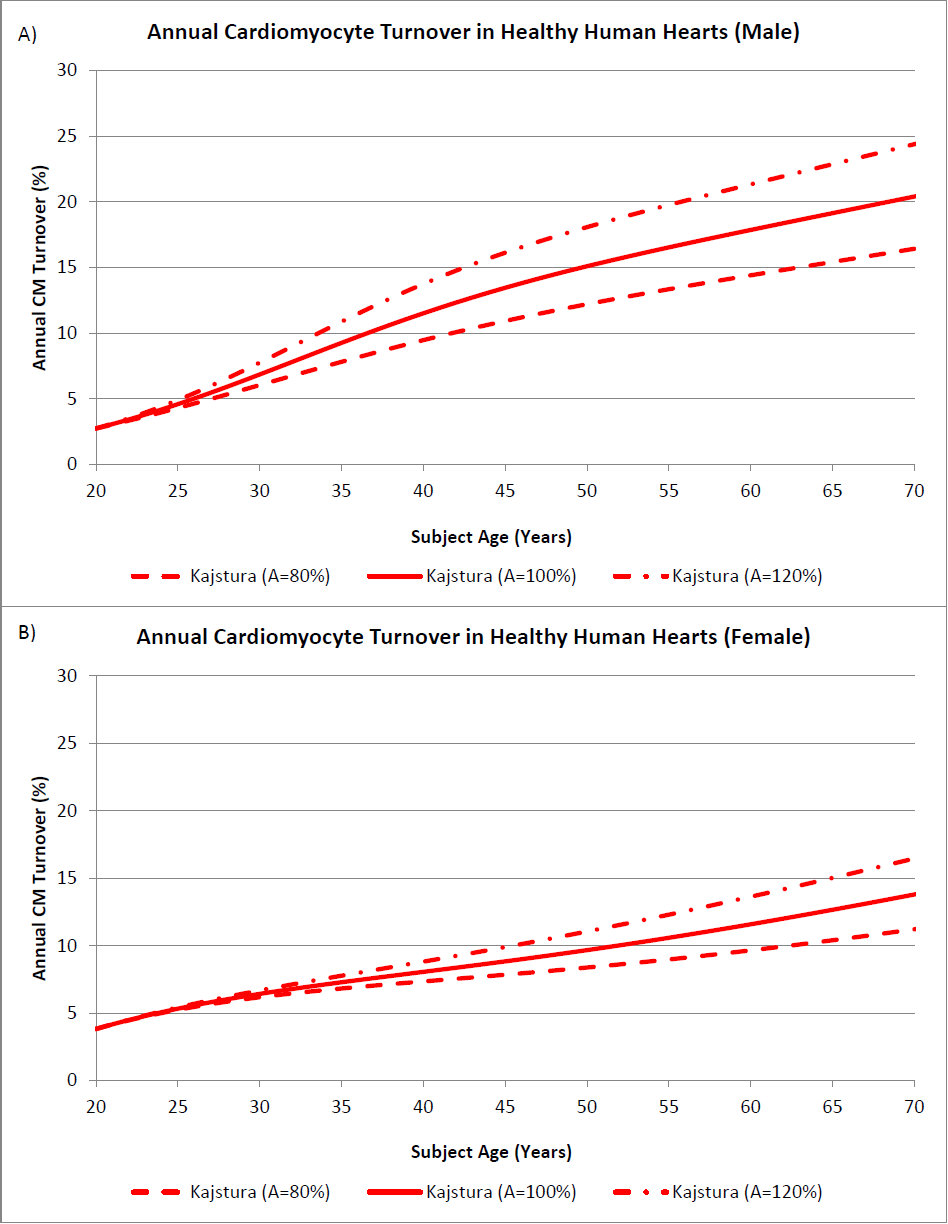

Supplement: Figure S14 — Sensitivity of the Kajstura Analysis Estimate of CM Turnover to the Apoptosis Fraction Variable. The Apoptosis Fraction is the measured percent of CM observed to be undergoing apoptosis at any given point in time. The hybrid model, under the adjusting turnover definition, was tested with the value of the fraction (which changes with subject age and is gender-specific) decreased by 20% (A = 80%) or increased by 20% (A = 120%). Sensitivity to the parameter A seems to be greatest when considering subjects of advanced age. Simulations were run for A) Male and B) Female. (TIF) [file pone.0051683.s014.tif]

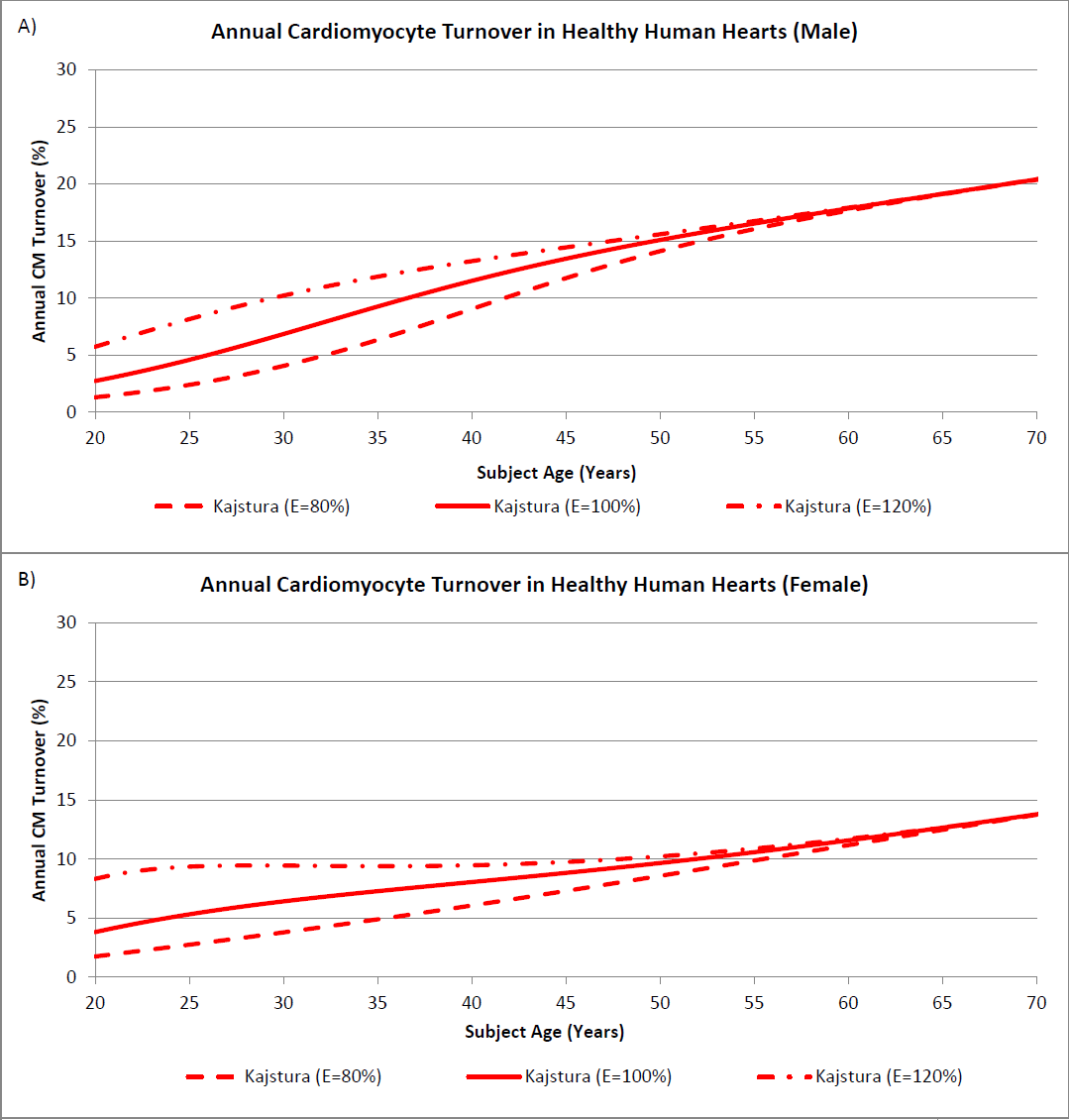

Supplement: Figure S15 — Sensitivity of the Kajstura Analysis Estimate of CM Turnover to the Expansion Exponent Variable. The Expansion Exponent is the number of divisions that a Cardiac Stem Cell is expected to undergo before becoming senescent (non-replicative). The hybrid model, under the adjusting turnover definition, was tested with the value of the fraction (which changes with subject age and is gender-specific) decreased by 20% (E = 80%) or increased by 20% (E = 120%). Sensitivity to the parameter E seems to be greatest when considering subjects of youthful age whereas in advanced age, changes in CM formation are roughly compensate for by changes in cumulative cell count. Simulations were run for A) Male and B) Female. (TIF) [file pone.0051683.s015.tif]

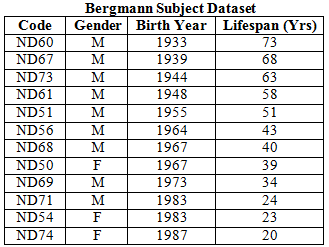

Supplement: Table S1 — Modeled Subject Genders, Birth Years, and Lifespans. Modeled subject input parameters were extracted from the Bergmann study. (TIF) [file pone.0051683.s016.tif]
