# Supplementary material for: Hybrid Mathematical Model of Cardiomyocyte Turnover in the Adult Human Heart
Source: PLoS One. 2012 Dec 19;7(12):e51683. doi: 10.1371/journal.pone.0051683 (PMC3526650; doi:10.1371/journal.pone.0051683)
Supplement: Code S1 — (DOCX) [file pone.0051683.s018.docx]

package turnovermodel;

import java.io.BufferedReader;

import java.io.BufferedWriter;

import java.io.FileNotFoundException;

import java.io.FileReader;

import java.io.FileWriter;

import java.io.IOException;

import java.util.StringTokenizer;

import java.util.logging.Level;

import java.util.logging.Logger;

import org.jdesktop.application.Application;

import org.jdesktop.application.SingleFrameApplication;

public class TurnoverModelApp extends SingleFrameApplication {

/*Read Me

Thank You for Studying Our Paper!

The complete code to run our model heart and regenerate our results is divided into two Java classes (TurnoverModelView and TurnoverModel)

TurnoverModel contains methods for basic tasks such as loading datapoints from databases, also contains global variables

TurnoverModelView contains a list of high-level methods (top of the class) that produce excel files (.csv) for output data/figures

TurnoverModelView contains lower level methods (middle of class) that perform basic model tasks that feed into the upper level methods

TurnoverModelView contains data retrieval methods (end of class) that return age and gender dependent parameters from published data

This program has no graphical user interface and is intended to be run from within an IDE such as Netbeans

This program requires 2 folders to exist in the working directory, so please create them manually when you download the program: IndividC14, RawDatabase

The RawDatabase folder requires the 9 .txt data files found in the supplement

If this setup has not been performed, the program will not run and you will not receive an error message

A method will fail to run if the .csv file it is intended to write to is open by the user. You will not receive an error message. Close the .csv file and re-run.

When the program has completed all scheduled runtime tasks, a generic window will popup. If this window does not pop up, the program is either still running or an error has occurred

Be aware of the output format of the .csv files. Data matrix structure is annotated in the individual methods that create the .csv of interest. Charts are not automatically generated from these .csv files.

We would like to acknowledge 3 primary sources of data for this program.

Kajstura J, Gurusamy N, Ogorek B, Goichberg P, Clavo-Rondon C, Hosoda T, D'Amario D, Bardelli S, Beltrami AP, Cesselli D, Bussani R, del Monte F, Quaini F, Rota M, Beltrami CA, Buchholz BA, Leri A, Anvera P, "Myocyte Turnover in the Aging Human Heart," Circ Res 2010, 107:1374-1386

Bergmann O, Bhardwaj RD, Bernard S, Zdunek S, Barnabe-Heider F, Walsh S, Zupicich J, Alkass K, Buchholz BA, Druid H, Jovinge S, Frisen J, "Evidence for Cardiomyocyte Renewal in Humans, Science 2009, 324,5923:98-102

Atmospheric Carbon14 over Time: CALIbomb web site (http://calib.qub.ac.uk/CALIBomb/frameset.html)

*/

@Override protected void startup() {try {

show(new TurnoverModelView(this));

} catch (IOException ex) {

Logger.getLogger(TurnoverModelApp.class.getName()).log(Level.SEVERE, null, ex);

}

}

@Override protected void configureWindow(java.awt.Window root) { }

public static TurnoverModelApp getApplication() { return Application.getInstance(TurnoverModelApp.class); }

public static void main(String[] args) {

launch(TurnoverModelApp.class, args);

}

public static String workingfold = "C:/Documents and Settings/jelser/Desktop/";

public static double cyclelengthhours = 26; //26 in paper

public static double cyclelengthhoursStdErr = 4;

public static double num20yroldMaleCM = 6*Math.pow(10,9);

public static double num20yroldFemaleCM=4*Math.pow(10,9);

public static double num20yroldMaleCM10g = 500*Math.pow(10,6);

public static double num20yroldFemaleCM10g = 500*Math.pow(10,6);

public static int numC14points=0;

public static double[][] C14points;

public static double scaleinterceptC14=1.0;

public static double scalemultiplierC14=1000;

public static double maxC14raw=1.92035;

public static int numBergmann=12;

public static double[][] Bergmann;

public static double delay=0;

public static int startage=19;

public static int Agerepetitions = 82;

public static double apopdurationhours = 4;//4 in paper

public static double CMvolume = 40000; //cubic microns, Not found in Kajstura. 20x20x100

public static double gramsperheart = 300;

public static double male5percAge = 0;

public static double female5percAge = 0;

public static double[][] holddata = new double[1][1];

public static int holdnumrows = 0;

public static int holdnumcol = 0;

public static double[][] holddata2=new double[1][1];

public static int holdnumrows2=0;

public static int holdnumcol2=0;

public static double[][] holddata3 = new double[1][1];

public static int holdnumrows3=0;

public static int holdnumcol3=0;

public static double[][] KajsturaFig7Histos;

public static int numKajsturaFig7;

public static int[] optimumMaleHistogramAges = {40,55,70};

public static int[] optimumFemaleHistogramAges = {49,69,85};

public static double initguess=0.001;//0.001 finds lowest for pre-bomb, 0.6 finds highest (For Scenario A, around 150%)

public static int counter =0; public static boolean applysubtract = true; public static double partialploidy=1.0;public static double addtoC14=0;

public static double modelerror=0;public static double[] modelparams;

public static double holddouble=0;public static double holddouble2=-1;public static double holddouble3=-1;

public static String curmethod = "None";

public static double[][] convertHistotoRiemannSum(double[][] data, int numyears){

//Assumes #CM of Age 0 = EndCount

double endcount = 0;

//Assumes Increment of 1 Year Between Rows

//Need numyears rows of data[i][0]=ageofCM and data[i][3] = %

double[][] riemannsum = new double[numyears+1][2]; //CM age and Riemann Sum %

for (int i=0;i<numyears-1;i++){

riemannsum[i][0]=data[i][0];

riemannsum[i][1]=(data[i][3]+data[i+1][3])/(2*1);

}

riemannsum[numyears-1][0]=1;

riemannsum[numyears-1][1]=(data[numyears-1][3]+endcount)/(2*1);

double cellcount=0;

for (int j=0;j<=numyears;j++){cellcount=cellcount+riemannsum[j][1];}

for (int j=0;j<=numyears;j++){riemannsum[j][1]=100*riemannsum[j][1]/cellcount;}

return riemannsum;

}

public static void fileWriter(double[][] data,int rows,int col, String dataname) throws IOException{

BufferedWriter OUT = new BufferedWriter(new FileWriter(TurnoverModelApp.workingfold+"/ApopModel/"+dataname+".csv"));

for (int i=0;i<rows;i++){

for (int j=0;j<col;j++){

OUT.write(data[i][j]+",");

}

OUT.write('\r');

}

OUT.close();

}

public static FileReader getfile(String unique) throws FileNotFoundException{

String filetype = ".txt";String preface = workingfold+"/ApopModel/";

FileReader r =new FileReader(preface+unique+filetype);

return r;

}

public static void BuildC14bybirthyear() throws IOException{

TurnoverModelApp.numC14points=914;

TurnoverModelApp.C14points=new double[TurnoverModelApp.numC14points][2];

BufferedReader compIN = null;

try{ compIN = new BufferedReader(TurnoverModelApp.getfile("/RawDatabase/RawBombYears"));}

catch(FileNotFoundException a){System.out.println("RawBombYears Not Found");}

String bombline = compIN.readLine();

for (int q=0;q<TurnoverModelApp.numC14points;q++){

StringTokenizer bombtok = new StringTokenizer(bombline," ");

TurnoverModelApp.C14points[q][0]=Double.parseDouble(bombtok.nextToken());

bombline = compIN.readLine();

}

compIN.close();

try{ compIN = new BufferedReader(TurnoverModelApp.getfile("/RawDatabase/RawBombC14"));}

catch(FileNotFoundException a){}

bombline = compIN.readLine();

for (int q=0;q<TurnoverModelApp.numC14points;q++){

StringTokenizer bombtok = new StringTokenizer(bombline," ");

TurnoverModelApp.C14points[q][1]=Double.parseDouble(bombtok.nextToken());

bombline = compIN.readLine();

}

//scale to Bergmann

for (int i=0;i<TurnoverModelApp.numC14points;i++){

TurnoverModelApp.C14points[i][1]=TurnoverModelApp.C14points[i][1]-TurnoverModelApp.scaleinterceptC14;

TurnoverModelApp.C14points[i][1]=TurnoverModelApp.C14points[i][1]*TurnoverModelApp.scalemultiplierC14/(TurnoverModelApp.maxC14raw-TurnoverModelApp.scaleinterceptC14);

}

compIN.close();

//Add In Pre-1959 data

double[][] holdC14points = new double[TurnoverModelApp.numC14points+(1959-1930)][2];

for (int i=0;i<1959-1930;i++){

holdC14points[i][0]=1930+i;

if (1930+i<1955){holdC14points[i][1]=-2;}

if(1930+i==1955){holdC14points[i][1]=0;}if(1930+i==1956){holdC14points[i][1]=25;}if(1930+i==1957){holdC14points[i][1]=80;}

if(1930+i==1958){holdC14points[i][1]=100;}

}

for (int i=0;i<TurnoverModelApp.numC14points;i++){

holdC14points[i+1959-1930][0]=TurnoverModelApp.C14points[i][0];

holdC14points[i+1959-1930][1]=TurnoverModelApp.C14points[i][1];

}

TurnoverModelApp.C14points=holdC14points;TurnoverModelApp.numC14points=TurnoverModelApp.numC14points+1959-1930;

}

public static double getinitialbergmannpatientC14level(int id){

double init=0;

if(TurnoverModelApp.delay==0){

//Standard Bomb Curve Response

//if (id==0){init=-2;}else if (id==1){init=-2;}else if (id==2){init=-2;}else if (id==3){init=-2;}else if (id==4){init=25;}else if (id==5){init=847;}

//else if (id==6){init=689;}else if (id==7){init=667;}else if (id==8){init=426;}else if (id==9){init=248;}else if (id==10){init=239;}else if (id==11){init=184;}

//Adjusted

if (id==0){init=-2;}else if (id==1){init=-2;}else if (id==2){init=-2;}else if (id==3){init=-2;}else if (id==4){init=-2;}else if (id==5){init=835;}

else if (id==6){init=647;}else if (id==7){init=653;}else if (id==8){init=417;}else if (id==9){init=238;}else if (id==10){init=236;}else if (id==11){init=178;}

}

/* else if (TurnoverModelApp.delay==2){

if (id==0){init=-2;}else if (id==1){init=-2;}else if (id==2){init=-2;}else if (id==3){init=-2;}else if (id==4){init=-2;}else if (id==5){init=313;}

else if (id==6){init=884;}else if (id==7){init=792;}else if (id==8){init=570;}else if (id==9){init=300;}else if (id==10){init=270;}else if (id==11){init=217;}

}*/

//To use lowest C14 values in same year

// if (id==0){init=-2;}else if (id==1){init=-2;}else if (id==2){init=-2;}else if (id==3){init=-2;}else if (id==4){init=0;}else if (id==5){init=809;}

// else if (id==6){init=639;}else if (id==7){init=639;}else if (id==8){init=402;}else if (id==9){init=230;}else if (id==10){init=230;}else if (id==11){init=211;}

//To use highest C14 values in same year

// if (id==0){init=-2;}else if (id==1){init=-2;}else if (id==2){init=-2;}else if (id==3){init=-2;}else if (id==4){init=25;}else if (id==5){init=1000;}

//else if (id==6){init=701;}else if (id==7){init=701;}else if (id==8){init=485;}else if (id==9){init=268;}else if (id==10){init=272;}else if (id==11){init=176;}

return init;

}

public static void ProduceBergmannData(){

//Use Figure 3d in Bergmann. Best Fit for Scenario A. Continuous Ploidy for Both Delta C14 (Fig 3d) and Turnover (Supp Tab 1)

double[][] bergmann = new double[12][10];

//0=ID, 1=gender(1=male,0=female), 2=birthyear, 3=ageatdeath, 4=Post-ploidcorrect dC14 (final, measured) from Bergmann Fig 3d,

//5=AveTurnover%PerYear pre-ploidy correction, AveTurnover(ContinuousPloidization)%PerYear, [7]=Pre-PloidyCorrected dC14 (Fig 3d, Table S1),

//8=k(DNA synthesis), 9=Pre-ploidcorrect dC14 (final, measured) from Bergmann Fig 3a/3b

bergmann[0][0]=1;bergmann[0][1]=1;bergmann[0][2]=1933.7;bergmann[0][3]=73.25;bergmann[0][4]=21.3;bergmann[0][5]=0.095;bergmann[0][6]=0.095;bergmann[0][7]=21.3-getinitialbergmannpatientC14level(0); bergmann[0][8]=79.5;bergmann[0][9]=21.3;

bergmann[1][0]=2;bergmann[1][1]=1;bergmann[1][2]=1939.6;bergmann[1][3]=67.58;bergmann[1][4]=18.84;bergmann[1][5]=0.085;bergmann[1][6]=0.085;bergmann[1][7]=18.84-getinitialbergmannpatientC14level(1); bergmann[1][8]=58.7;bergmann[1][9]=18.84;

bergmann[2][0]=3;bergmann[2][1]=1;bergmann[2][2]=1944.6;bergmann[2][3]=62.67;bergmann[2][4]=3.65;bergmann[2][5]=0.0049;bergmann[2][6]=0.0049;bergmann[2][7]=3.65-getinitialbergmannpatientC14level(2); bergmann[2][8]=72.2;bergmann[2][9]=3.65;

bergmann[3][0]=4;bergmann[3][1]=1;bergmann[3][2]=1948.6;bergmann[3][3]=58.42;bergmann[3][4]=60;bergmann[3][5]=0.54;bergmann[3][6]=0.42;bergmann[3][7]=69.45-getinitialbergmannpatientC14level(3); bergmann[3][8]=101.5;bergmann[3][9]=80;

bergmann[4][0]=5;bergmann[4][1]=1;bergmann[4][2]=1955.9;bergmann[4][3]=50.83;bergmann[4][4]=40;bergmann[4][5]=0.79;bergmann[4][6]=0.40;bergmann[4][7]=194.6-getinitialbergmannpatientC14level(4); bergmann[4][8]=57.4;bergmann[4][9]=185;

bergmann[5][0]=6;bergmann[5][1]=1;bergmann[5][2]=1964.1;bergmann[5][3]=42.75;bergmann[5][4]=515;bergmann[5][5]=1.58;bergmann[5][6]=1.53;bergmann[5][7]=443.24-getinitialbergmannpatientC14level(5); bergmann[5][8]=74.0;bergmann[5][9]=455;

bergmann[6][0]=7;bergmann[6][1]=1;bergmann[6][2]=1967.4;bergmann[6][3]=39.83;bergmann[6][4]=455;bergmann[6][5]=1.36;bergmann[6][6]=1.14;bergmann[6][7]=403.08-getinitialbergmannpatientC14level(6); bergmann[6][8]=52.7;bergmann[6][9]=405;

bergmann[7][0]=8;bergmann[7][1]=0;bergmann[7][2]=1967.7;bergmann[7][3]=39.00;bergmann[7][4]=405;bergmann[7][5]=1.65;bergmann[7][6]=1.63;bergmann[7][7]=355.7-getinitialbergmannpatientC14level(7); bergmann[7][8]=83.1;bergmann[7][9]=360;

bergmann[8][0]=9;bergmann[8][1]=1;bergmann[8][2]=1973.5;bergmann[8][3]=33.75;bergmann[8][4]=330;bergmann[8][5]=1.48;bergmann[8][6]=1.36;bergmann[8][7]=279.94-getinitialbergmannpatientC14level(8); bergmann[8][8]=64.5;bergmann[8][9]=280;

bergmann[9][0]=10;bergmann[9][1]=1;bergmann[9][2]=1983.5;bergmann[9][3]=23.75;bergmann[9][4]=200;bergmann[9][5]=0.90;bergmann[9][6]=0.93;bergmann[9][7]=178.4-getinitialbergmannpatientC14level(9); bergmann[9][8]=74.5;bergmann[9][9]=180;

bergmann[10][0]=11;bergmann[10][1]=0;bergmann[10][2]=1983.8;bergmann[10][3]=23.00;bergmann[10][4]=195;bergmann[10][5]=1.51;bergmann[10][6]=1.48;bergmann[10][7]=167.38-getinitialbergmannpatientC14level(10);bergmann[10][8]=68.5;bergmann[10][9]=170;

bergmann[11][0]=12;bergmann[11][1]=0;bergmann[11][2]=1987.2;bergmann[11][3]=20;bergmann[11][4]=155;bergmann[11][5]=1.86;bergmann[11][6]=1.87;bergmann[11][7]=141.2-getinitialbergmannpatientC14level(11);bergmann[11][8]=67.8;bergmann[11][9]=140;

TurnoverModelApp.Bergmann=bergmann; TurnoverModelApp.numBergmann=12;

}

public static void LoadFig7Histograms() throws IOException{

int maxAnversaAge = 20;

double[][] Histos = new double[maxAnversaAge][7];//0=AgeofCM, 1=M-Yng(%),2=M-Mid(%),3=M-Old(%),4=F-Yng(%),5=F-Mid(%),6=F-Old(%)

BufferedReader compIN = null;

try{ compIN = new BufferedReader(TurnoverModelApp.getfile("/RawDatabase/RawFig7Years"));}catch(FileNotFoundException a){}

for (int q=0;q<maxAnversaAge;q++){Histos[q][0]=Double.parseDouble(compIN.readLine());}

try{ compIN = new BufferedReader(TurnoverModelApp.getfile("/RawDatabase/RawMYoungFig7"));}catch(FileNotFoundException a){}

for (int q=0;q<maxAnversaAge;q++){Histos[q][1]=Double.parseDouble(compIN.readLine());}

try{ compIN = new BufferedReader(TurnoverModelApp.getfile("/RawDatabase/RawMMiddleFig7"));}catch(FileNotFoundException a){}

for (int q=0;q<maxAnversaAge;q++){Histos[q][2]=Double.parseDouble(compIN.readLine());}

try{ compIN = new BufferedReader(TurnoverModelApp.getfile("/RawDatabase/RawMOldFig7"));}catch(FileNotFoundException a){}

for (int q=0;q<maxAnversaAge;q++){Histos[q][3]=Double.parseDouble(compIN.readLine());}

try{ compIN = new BufferedReader(TurnoverModelApp.getfile("/RawDatabase/RawFYoungFig7"));}catch(FileNotFoundException a){}

for (int q=0;q<maxAnversaAge;q++){Histos[q][4]=Double.parseDouble(compIN.readLine());}

try{ compIN = new BufferedReader(TurnoverModelApp.getfile("/RawDatabase/RawFMiddleFig7"));}catch(FileNotFoundException a){}

for (int q=0;q<maxAnversaAge;q++){Histos[q][5]=Double.parseDouble(compIN.readLine());}

try{ compIN = new BufferedReader(TurnoverModelApp.getfile("/RawDatabase/RawFOldFig7"));}catch(FileNotFoundException a){}

for (int q=0;q<maxAnversaAge;q++){Histos[q][6]=Double.parseDouble(compIN.readLine());}

compIN.close();

TurnoverModelApp.KajsturaFig7Histos=Histos;

TurnoverModelApp.numKajsturaFig7=maxAnversaAge;

}

public static void printpercDC14attributedtoploidybybergmann(){

for (int i=0;i<TurnoverModelApp.numBergmann;i++){

double p = 1-TurnoverModelApp.Bergmann[i][4]/TurnoverModelApp.Bergmann[i][7];

System.out.println(p);

}

}

public static int findpatientindex(int lifespan){

int index=-1;

for (int i=0;i<TurnoverModelApp.numBergmann;i++){

if (lifespan==(int)TurnoverModelApp.Bergmann[i][3]){

index=i;

}

}

return index;

}

public static double getTakamatsuPloidy(int age){

double fa =0;

if (age<22){ //all patient deaths are over age 9

fa=113.6+((135.4-113.6)/(22-9))*(age-9);

}

else if (age>22){

fa=135.4+((148.8-135.4)/(75-22))*(age-22);

}

return fa;

}

}

package turnovermodel;

import java.io.IOException;

import org.jdesktop.application.Action;

import org.jdesktop.application.ResourceMap;

import org.jdesktop.application.SingleFrameApplication;

import org.jdesktop.application.FrameView;

import org.jdesktop.application.TaskMonitor;

import java.awt.event.ActionEvent;

import java.awt.event.ActionListener;

import java.io.BufferedReader;

import java.io.FileNotFoundException;

import java.util.NoSuchElementException;

import java.util.StringTokenizer;

import javax.swing.Timer;

import javax.swing.Icon;

import javax.swing.JDialog;

import javax.swing.JFrame;

public class TurnoverModelView extends FrameView {

public TurnoverModelView(SingleFrameApplication app) throws IOException {

super(app);

//Do Not Comment-Out the Following Block of Programs, They are Required to Run the Program

initComponents();

TurnoverModelApp.ProduceBergmannData();

TurnoverModelApp.BuildC14bybirthyear();

TurnoverModelApp.LoadFig7Histograms();

//You May Comment-Out These Programs or Erase Comments to Activate the Script for Runtime

//Reproduction of Kajstura Results Using Automaton Model

//ProduceFig1Ecmdepletion(); //Demonstrates Model Apoptosis Module Ability to Reproduce Kajstura Publication Figure1E (Also Prints Year of 95% Reduction of CM for Comparison to Kajstura)

//HierarchModel10gCompareOnlyApop(); //Shows Apoptosis Module Agreement with Kajstura Publication for Both Male and Female

//HierarchModel10gCompareOnlyBirthRateFig6b(); //Shows Myocyte Formation with Age for Both Genders (Compare to Kajstura Figure6b)

//ProduceAnnTurnoverFig6c(); //Shows Turnover (Derived from Myocyte Formation) with Age for Both Genders (Compare to Kajstura Figure6c)

//GetKajsturaTurnoverDenominatorinHeart();

//ProduceKajsturaModelCMoverTime();

//Vary Kajstura Parameters and View Effect on Kajstura Outputs

//VaryBirthCycleDuration(); //Creates Myocyte Formation Over Patient Age for Both Genders for Various Stem Cell Progenitor Turnover Levels

// KajsturaModelTurnover(); //Creates Turnover Rates Over Patient Age for Both Genders with Total CM Count Allowed to Vary (Compares to Reported (Fixed CM) Kajstura Results)

//VaryApopRateforHistograms(); //Creates Myocyte-Age Histograms at Time of Death for Young, Middle-Aged, and Old Patients (Both Genders) for Various Apoptosis Rates

//VaryHalfLifeforHistograms(); //Creates Myocyte-Age Histograms at Time of Death for Young, Middle-Aged, and Old Patients (Both Genders) for Various Half-Life Parameters

//VaryExpansionExponentforTurnover(); //Creates Myocyte Formation Over Patient Age for Both Genders for Various Stem Cell Progenitor Turnover Levels Varying Gt Exponent

//Reproduce Bergmann Results

//PrintBombFunction(); //Displays Atmospheric C14 Data, CALIbomb website accessed 3/28/2011 (http://calib.qub.ac.uk/CALIBomb/frameset.html). 1-Year Smoothing of Levin Dataset.

//TestHybridModelforBergmann();

//TestScenarioA(); //Converts Published DeltaC14 Values for 12 Patients to Published Scenario A Turnover Rates

//IdentifyBifurcation();

//Evaluate Bergmann

//BergmannSensitivityToTrueTurnover();

//TestPloidy(); //Tests effect of Ploidization Variation

GlobalFit();

//GetC14forScenarioE2();

//GetCMAgeHistogramwithGlobal(); //Manually set turnover parameters of interest and measure age histograms for patients

//Combine Bergmann and Kajstura Model Results

//AnversaAgeHistogramforBergmannPatients(); //Generates an Age Histogram for Each of the 12 Bergmann Patients Based on Their Lifespans

//CompareAversaBergmannPercent(); //Generates a Comparison of Bergmann Ploidy-Corrected Turnover Rates for the 12 Bergmann Patients and Compares to Modeled (Kajstura Parameterized) Hearts of Same Simulated Birthdates and Lifespans

//VaryHalfLifeforC14andPerc(); //Computes Annual Turnover Rates for the 12 Bergmann Patients, Modeled by Automaton with Kajstura Parameters for Various Halflife Parameter Values

// status bar initialization - message timeout, idle icon and busy animation, etc

ResourceMap resourceMap = getResourceMap();

int messageTimeout = resourceMap.getInteger("StatusBar.messageTimeout");

messageTimer = new Timer(messageTimeout, new ActionListener() {

public void actionPerformed(ActionEvent e) {

statusMessageLabel.setText("");

}

});

messageTimer.setRepeats(false);

int busyAnimationRate = resourceMap.getInteger("StatusBar.busyAnimationRate");

for (int i = 0; i < busyIcons.length; i++) {

busyIcons[i] = resourceMap.getIcon("StatusBar.busyIcons[" + i + "]");

}

busyIconTimer = new Timer(busyAnimationRate, new ActionListener() {

public void actionPerformed(ActionEvent e) {

busyIconIndex = (busyIconIndex + 1) % busyIcons.length;

statusAnimationLabel.setIcon(busyIcons[busyIconIndex]);

}

});

idleIcon = resourceMap.getIcon("StatusBar.idleIcon");

statusAnimationLabel.setIcon(idleIcon);

progressBar.setVisible(false);

// connecting action tasks to status bar via TaskMonitor

TaskMonitor taskMonitor = new TaskMonitor(getApplication().getContext());

taskMonitor.addPropertyChangeListener(new java.beans.PropertyChangeListener() {

public void propertyChange(java.beans.PropertyChangeEvent evt) {

String propertyName = evt.getPropertyName();

if ("started".equals(propertyName)) {

if (!busyIconTimer.isRunning()) {

statusAnimationLabel.setIcon(busyIcons[0]);

busyIconIndex = 0;

busyIconTimer.start();

}

//progressBar.setVisible(true);

progressBar.setIndeterminate(true);

} else if ("done".equals(propertyName)) {

busyIconTimer.stop();

statusAnimationLabel.setIcon(idleIcon);

progressBar.setVisible(false);

progressBar.setValue(0);

} else if ("message".equals(propertyName)) {

String text = (String)(evt.getNewValue());

statusMessageLabel.setText((text == null) ? "" : text);

messageTimer.restart();

} else if ("progress".equals(propertyName)) {

int value = (Integer)(evt.getNewValue());

// progressBar.setVisible(true);

progressBar.setIndeterminate(false);

progressBar.setValue(value);

}

}

});

}

public void ProduceFig1Ecmdepletion() throws IOException{

int agerep = TurnoverModelApp.Agerepetitions;

int startage = TurnoverModelApp.startage+1;

boolean hasbeenfoundmale=false;boolean hasbeenfoundfemale=false;

double[][] maleCMaliveMillion = new double[agerep][2];

maleCMaliveMillion[0][0]=startage;

maleCMaliveMillion[0][1]=TurnoverModelApp.num20yroldMaleCM;

int age=startage;int repetitions = agerep;

for (int i=1;i<repetitions;i++){

maleCMaliveMillion[i][0]=age+1;

double startyearCM = maleCMaliveMillion[i-1][1];

int numAnnualCycles = (int) Math.rint(365*24/TurnoverModelApp.apopdurationhours);

for (int j=0;j<numAnnualCycles;j++){

double apopLost = ApopCMperMillionCM("Male",age)*(startyearCM/Math.pow(10,6));

startyearCM=startyearCM-apopLost;

}

maleCMaliveMillion[i][1]=startyearCM;

if (hasbeenfoundmale==false&&maleCMaliveMillion[i][1]<TurnoverModelApp.num20yroldMaleCM*0.05){

hasbeenfoundmale=true;TurnoverModelApp.male5percAge=maleCMaliveMillion[i][0];

}

age++;

}

double[][] femaleCMaliveMillion = new double[agerep][2];

femaleCMaliveMillion[0][0]=startage;

femaleCMaliveMillion[0][1]=TurnoverModelApp.num20yroldFemaleCM;

age=startage; repetitions = agerep;

for (int i=1;i<repetitions;i++){

femaleCMaliveMillion[i][0]=age+1;

double startyearCM = femaleCMaliveMillion[i-1][1];

int numAnnualCycles = (int) Math.rint(365*24/TurnoverModelApp.apopdurationhours);

for (int j=0;j<numAnnualCycles;j++){

double apopLost = ApopCMperMillionCM("Female",age)*(startyearCM/Math.pow(10,6));

startyearCM=startyearCM-apopLost;

}

femaleCMaliveMillion[i][1]=startyearCM;

if (hasbeenfoundfemale==false&&femaleCMaliveMillion[i][1]<TurnoverModelApp.num20yroldFemaleCM*0.05){

hasbeenfoundfemale=true;TurnoverModelApp.female5percAge=femaleCMaliveMillion[i][0];

}

age++;

}

System.out.println("Age of 95% Reduction in CM: "+TurnoverModelApp.male5percAge+"-Male, "+TurnoverModelApp.female5percAge+"-Female");

double[][] combined = new double[repetitions][3];

for (int k=0;k<repetitions;k++){

combined[k][0]=maleCMaliveMillion[k][0];combined[k][1]=maleCMaliveMillion[k][1];combined[k][2]=femaleCMaliveMillion[k][1];

}

TurnoverModelApp.holddata=combined;

TurnoverModelApp.fileWriter(combined,repetitions , 3, "ApoptosisFig1E");

}

public void AnversaAgeHistogramforBergmannPatients() throws IOException{

double gtmult=1;

int numtoshow = 25;

double[][] data = new double[numtoshow][TurnoverModelApp.numBergmann+1]; //columns are age histograms (to 25 years) for each patient

for (int i=0;i<numtoshow;i++){data[i][0]=numtoshow-i;}

for (int g=0;g<TurnoverModelApp.numBergmann;g++){

int[] ages = {(int)TurnoverModelApp.Bergmann[g][3],(int)TurnoverModelApp.Bergmann[g][3],(int)TurnoverModelApp.Bergmann[g][3]}; //Dummy ages[] to satisfy CompareHistograms

String gender = "Male"; if (TurnoverModelApp.Bergmann[g][1]==0){gender = "Female";}

CompareHistogramsforVariousPatientAges(gender,ages,1.0,gtmult);

for (int i=0;i<numtoshow;i++){

data[i][g+1]= TurnoverModelApp.holddata2[i+TurnoverModelApp.Agerepetitions+TurnoverModelApp.startage-numtoshow][1];

}

}

TurnoverModelApp.fileWriter(data,numtoshow,TurnoverModelApp.numBergmann+1,"BergmannAgeHistosbyAnversa");

}

public double ComputeDeltaC14forConstantTurnover(int patient, double turn, boolean ploidyindependent) throws IOException{

turn = turn + 0.0; //must be a decimal (int value will cause error)

int birthyear = (int)TurnoverModelApp.Bergmann[patient][2]; int deathyear = (int)(TurnoverModelApp.Bergmann[patient][2]+(int)TurnoverModelApp.Bergmann[patient][3]);

int lifespan= (int)TurnoverModelApp.Bergmann[patient][3];

double[][] agedist = new double[lifespan][4]; //0=CMage, 3=CMcount at Age

for (int j=0;j<lifespan;j++){

agedist[j][0]=lifespan-j; //CM Age

if (j>0){agedist[j][3]=turn*Math.pow((1-turn),lifespan-j);}

else if (j==0){agedist[j][3]=Math.pow((1-turn),lifespan);}

}

double[][] riemanndata = TurnoverModelApp.convertHistotoRiemannSum(agedist, deathyear-birthyear);

//riemanndata = agedist;

double[][] data = new double[deathyear-birthyear][3]; //0=AgeofCM, 1=#CMProducedofAge, 2=C14atDateofProduction

for (int i=0;i<deathyear-birthyear;i++){

data[i][0]=riemanndata[i][0];

data[i][1]=riemanndata[i][1];

data[i][2]=GetC14atyear(birthyear+i);

}

double[] aveC14postploidy=new double[lifespan];for (int w=0;w<lifespan;w++){aveC14postploidy[w]=data[w][2];}

if (ploidyindependent==false){ //remove ploidy

for (int p=0;p<lifespan;p++){ //each cell-age cohort contributes a certain amount of C14. p = year of pt life

double aveC14foragegroup = data[p][2];

for (int q=p;q<lifespan;q++){ //q = years after cell born in patient life p

double percadded = computepersonalizedpolyploidyc14rate(patient, q,1);

aveC14foragegroup = (aveC14foragegroup + percadded*data[q][2])/(1+percadded);

}

data[p][2]=aveC14foragegroup;

}

}

double CMcount=0; double C14count=0;

for (int i=0;i<deathyear-birthyear;i++){

CMcount=CMcount+data[i][1];C14count=C14count+data[i][1]*data[i][2];

}

double aveC14 = C14count/CMcount;

//Normalize for PrintOut

double c1 = 0;double[][] normdata = data;

for (int i=0;i<deathyear-birthyear;i++){c1=c1+normdata[i][1];}

for (int i=0;i<deathyear-birthyear;i++){normdata[i][1]=100*normdata[i][1]/c1;}

TurnoverModelApp.fileWriter(normdata,deathyear-birthyear,3,"/IndividC14/C14"+birthyear+deathyear);

System.out.println(patient+" AveC14 "+aveC14+", DeltaC14 "+(aveC14-TurnoverModelApp.getinitialbergmannpatientC14level(patient)));

return aveC14-TurnoverModelApp.getinitialbergmannpatientC14level(patient);

//return (aveC14-GetC14atyear((double)(birthyear)));

}

public void VaryBirthCycleDuration() throws IOException{

double holdbirthcycle = TurnoverModelApp.cyclelengthhours;

//Datafile = A=Age, B=CM(CycLengthFactor=1/2), B=CM(CycLengthFactor=1), C=CM(CycLengthFactor=3/2)

double[][] data = new double[TurnoverModelApp.Agerepetitions][4];

for (int p=1;p<=3;p++){

TurnoverModelApp.cyclelengthhours=holdbirthcycle*(0.5*p);

HierarchicalModel10g("Male",false,true,false,1,1,1);

for (int i=1;i<TurnoverModelApp.Agerepetitions;i++){

data[i][p]=TurnoverModelApp.holddata[i][7];

if(p==1){data[i][0]=TurnoverModelApp.holddata[i][0];}

}

}

TurnoverModelApp.fileWriter(data, TurnoverModelApp.Agerepetitions, 4, "VaryBirthRate-M");

TurnoverModelApp.cyclelengthhours=holdbirthcycle;

data = new double[TurnoverModelApp.Agerepetitions][4];

for (int p=1;p<=3;p++){

TurnoverModelApp.cyclelengthhours=holdbirthcycle*(0.5*p);

HierarchicalModel10g("Female",false,true,false,1,1,1);

for (int i=1;i<TurnoverModelApp.Agerepetitions;i++){

data[i][p]=TurnoverModelApp.holddata[i][7];

if(p==1){data[i][0]=TurnoverModelApp.holddata[i][0];}

}

}

TurnoverModelApp.fileWriter(data, TurnoverModelApp.Agerepetitions, 4, "VaryBirthRate-F");

TurnoverModelApp.cyclelengthhours=holdbirthcycle;

}

public void ProduceAnnTurnoverFig6c() throws IOException{

//Comparing Kajstura Fig 6

boolean allowapop=false;

TurnoverModelApp.curmethod="AllowNumCellstoFluctuate";allowapop=true; //Comment out if testing Kajstura turnover as done in paper (turnover=new cells/constant old cells), allow if testing turnover = new cells/fluctuating old cells

//Hierchical Model Params: String gender,boolean includeapop,boolean includebirth, boolean includeCMsen,double gtmult, double apopfractmult,double apopdurationmult

//Datafile = A=Age, B=M-KajsturaAnnCMTurnover%,C=M-MyModelAnnCMTurnover%,D=F-KajsturaAnnCMTurnover%,E=F-MyModelAnnCMTurnover%

int rep=TurnoverModelApp.Agerepetitions;double[][] data = new double[rep][5];

//Test My Model

HierarchicalModel10g("Male",allowapop,true,false,.8,1,1); //GT, Apop Fract, ApopDur

data[0][0]=TurnoverModelApp.holddata[0][0];data[0][2]=TurnoverModelApp.holddata[0][6];

for (int i=1;i<rep;i++){data[i][0]=TurnoverModelApp.holddata[i][0];data[i][2]=TurnoverModelApp.holddata[i][6];}

HierarchicalModel10g("Female",allowapop,true,false,.8,1,1);

data[0][4]=TurnoverModelApp.holddata[0][6];

for (int i=1;i<rep;i++){data[i][4]=TurnoverModelApp.holddata[i][6];}

//Test Kajstura Model

for (int i=0;i<rep;i++){

data[i][1]=AnnTurnoverCombinedModel("Male",data[i][0]);

data[i][3]=AnnTurnoverCombinedModel("Female",data[i][0]);

}

TurnoverModelApp.fileWriter(data, rep, 5, "Figure6C");

}

public void ProduceKajsturaModelCMoverTime() throws IOException{

//Compare Effect of Apoptosis, Senescence, and Birth

//Datafile = A=Age, B=CM(OnlyApop), C=CM(Apop,Birth), D=CM(Apop,Birth,Senescence)

int rep=TurnoverModelApp.Agerepetitions;double[][] data = new double[rep][5];

HierarchicalModel10g("Male",true,false,false,1,1,1);

data[0][0]=TurnoverModelApp.holddata[0][0];data[0][1]=TurnoverModelApp.num20yroldMaleCM10g;

for (int i=1;i<rep;i++){data[i][0]=TurnoverModelApp.holddata[i][0]+1;data[i][1]=TurnoverModelApp.holddata[i][5];}

HierarchicalModel10g("Male",true,true,false,1,1,1);

data[0][0]=TurnoverModelApp.holddata[0][0];data[0][2]=TurnoverModelApp.num20yroldMaleCM10g;

for (int i=1;i<rep;i++){data[i][2]=TurnoverModelApp.holddata[i][5];}

HierarchicalModel10g("Male",true,true,true,1,1,1);

data[0][0]=TurnoverModelApp.holddata[0][0];data[0][3]=TurnoverModelApp.num20yroldMaleCM10g;

for (int i=1;i<rep;i++){data[i][3]=TurnoverModelApp.holddata[i][5];}

TurnoverModelApp.fileWriter(data, rep, 4, "KajsturaModel-M");

HierarchicalModel10g("Female",true,false,false,1,1,1);

data[0][0]=TurnoverModelApp.holddata[0][0];data[0][1]=TurnoverModelApp.num20yroldFemaleCM10g;

for (int i=1;i<rep;i++){data[i][0]=TurnoverModelApp.holddata[i][0];data[i][1]=TurnoverModelApp.holddata[i][5];}

HierarchicalModel10g("Female",true,true,false,1,1,1);

data[0][0]=TurnoverModelApp.holddata[0][0];data[0][2]=TurnoverModelApp.num20yroldFemaleCM10g;

for (int i=1;i<rep;i++){data[i][2]=TurnoverModelApp.holddata[i][5];}

HierarchicalModel10g("Female",true,true,true,1,1,1);

data[0][0]=TurnoverModelApp.holddata[0][0];data[0][3]=TurnoverModelApp.num20yroldFemaleCM10g;

for (int i=1;i<rep;i++){data[i][3]=TurnoverModelApp.holddata[i][5];}

TurnoverModelApp.fileWriter(data, rep, 4, "KajsturaModel-F");

}

public void HierarchModel10gCompareOnlyApop() throws IOException{

//Comparing Kajstura Fig 1

//Datafile = A=Age, B=M-KajsturaApopScaled10g,C=M-MyModelApop10g,D=F-KajsturaApopScaled10g,E=F-MyModelApop10g

int rep=TurnoverModelApp.Agerepetitions;double[][] data = new double[rep][5];

double holdnum20yearoldMaleCM=TurnoverModelApp.num20yroldMaleCM;

double holdnum20yearoldFemaleCM=TurnoverModelApp.num20yroldFemaleCM;

//Test Kajstura Models Scaled to 10g

TurnoverModelApp.num20yroldMaleCM=TurnoverModelApp.num20yroldMaleCM10g;

TurnoverModelApp.num20yroldFemaleCM=TurnoverModelApp.num20yroldFemaleCM10g;

ProduceFig1Ecmdepletion();//Holdata COL0=age,COL1=maleWholeHeart,COL2=femaleWholeHeart

boolean malefound=false; boolean femalefound=false;double malestartCM=TurnoverModelApp.holddata[0][1];double femalestartCM=TurnoverModelApp.holddata[0][2];

for (int i=0;i<rep;i++){

data[i][0]=TurnoverModelApp.holddata[i][0];data[i][1]=TurnoverModelApp.holddata[i][1];data[i][3]=TurnoverModelApp.holddata[i][2];

if (TurnoverModelApp.holddata[i][1]<0.05*malestartCM&&!malefound){malefound=true;System.out.println("Kajstura Model Male 5% Year = "+data[i][0]);}

if (TurnoverModelApp.holddata[i][2]<0.05*femalestartCM&&!femalefound){femalefound=true;System.out.println("Kajstura Model Female 5% Year = "+data[i][0]);}

}

HierarchicalModel10g("Male",true,false,false,1,1,1);

boolean found = false;double startCM = TurnoverModelApp.holddata[0][4];

for (int i=0;i<rep;i++){data[i][2]=TurnoverModelApp.holddata[i][4];

if (TurnoverModelApp.holddata[i][4]<0.05*startCM&&!found){found=true;System.out.println("My Model Male 5% Year = "+data[i][0]);}

}

HierarchicalModel10g("Female",true,false,false,1,1,1);

found = false; startCM = TurnoverModelApp.holddata[0][4];

for (int i=0;i<rep;i++){data[i][4]=TurnoverModelApp.holddata[i][4];

if (TurnoverModelApp.holddata[i][4]<0.05*startCM&&!found){found=true;System.out.println("My Model Female 5% Year = "+data[i][0]);}

}

TurnoverModelApp.fileWriter(data, rep, 5, "CompareModelsofApopAlone");

TurnoverModelApp.num20yroldMaleCM=holdnum20yearoldMaleCM;

TurnoverModelApp.num20yroldFemaleCM=holdnum20yearoldFemaleCM;

}

public void HierarchModel10gCompareOnlyBirthRateFig6b() throws IOException{

//Comparing Kajstura Fig 6

//Datafile = A=Age, B=M-KajsturaAnnCMform10g/Million,C=M-MyModelAnnCMform10g/Million,D=F-KajsturaAnnCMform10g/Million,E=F-MyModelAnnCMform10g/Million

int rep=TurnoverModelApp.Agerepetitions;double[][] data = new double[rep][5];

//Test My Model

HierarchicalModel10g("Male",false,true,false,1,1,1);

double startCM = TurnoverModelApp.holddata[0][4];

data[0][0]=TurnoverModelApp.holddata[0][0];data[0][2]=TurnoverModelApp.holddata[0][4]-startCM;

for (int i=1;i<rep;i++){data[i][0]=TurnoverModelApp.holddata[i][0];data[i][2]=(TurnoverModelApp.holddata[i][3]-TurnoverModelApp.holddata[i-1][3])/Math.pow(10,6);}

HierarchicalModel10g("Female",false,true,false,1,1,1);

startCM = TurnoverModelApp.holddata[0][4];

data[0][4]=TurnoverModelApp.holddata[0][4]-startCM;

for (int i=1;i<rep;i++){data[i][4]=(TurnoverModelApp.holddata[i][3]-TurnoverModelApp.holddata[i-1][3])/Math.pow(10,6);}

//Test Kajstura Model

for (int i=0;i<rep;i++){

data[i][1]=MillionsMyocyteFormationCombinedModelPer10gPerYear("Male",data[i][0]);

data[i][3]=MillionsMyocyteFormationCombinedModelPer10gPerYear("Female",data[i][0]);

}

TurnoverModelApp.fileWriter(data, rep, 5, "CompareModelsofBirthAlone");

}

public void KajsturaModelTurnover() throws IOException{

int rep=TurnoverModelApp.Agerepetitions;double[][] data = new double[rep][7];

//Datefile = A=age,B=PublishedTurnover,C=BirthRate/MyModelCellsinHeart

String gender = "Male";

HierarchicalModel10g(gender,true,true,true,1,1,1);

for (int i=0;i<rep;i++){

data[i][0]=TurnoverModelApp.holddata[i][0];

data[i][1]=AnnTurnoverCombinedModel(gender,data[i][0]);

data[i][2]=100*TurnoverModelApp.holddata[i][7]/TurnoverModelApp.holddata[i][2];

}

gender = "Female";

HierarchicalModel10g(gender,true,true,true,1,1,1);

for (int i=0;i<rep;i++){

data[i][4]=TurnoverModelApp.holddata[i][0];

data[i][5]=AnnTurnoverCombinedModel(gender,data[i][0]);

data[i][6]=100*TurnoverModelApp.holddata[i][7]/TurnoverModelApp.holddata[i][2];

}

TurnoverModelApp.fileWriter(data,rep,7,"TurnoverComparison");

}

public void VaryApopRateforHistograms() throws IOException{

double gtmult=1;

int numtoshow = 25;double[][] data = new double[numtoshow][10]; //0=Age(25->1),1=Y-80%,2=Y-100%,3=Y-120%,4=M-80%,5=M-100%,6=M-120%,7=O-80%,8=O-100%,9=O-120%

double holdapop = TurnoverModelApp.apopdurationhours;

for (int i=0;i<numtoshow;i++){data[i][0]=numtoshow-i;}

String gender = "Male"; int[] ages = new int[3]; ages[0]=49;ages[1]=69;ages[2]=74;

TurnoverModelApp.apopdurationhours=holdapop*0.8;

CompareHistogramsforVariousPatientAges(gender,ages,1,gtmult);

for (int i=0;i<numtoshow;i++){

data[i][1]= TurnoverModelApp.holddata2[i+TurnoverModelApp.Agerepetitions+TurnoverModelApp.startage-numtoshow][1];

data[i][4]= TurnoverModelApp.holddata2[i+TurnoverModelApp.Agerepetitions+TurnoverModelApp.startage-numtoshow][2];

data[i][7]= TurnoverModelApp.holddata2[i+TurnoverModelApp.Agerepetitions+TurnoverModelApp.startage-numtoshow][3];

}

TurnoverModelApp.apopdurationhours=holdapop*1.0;

CompareHistogramsforVariousPatientAges(gender,ages,1,gtmult);

for (int i=0;i<numtoshow;i++){

data[i][2]= TurnoverModelApp.holddata2[i+TurnoverModelApp.Agerepetitions+TurnoverModelApp.startage-numtoshow][1];

data[i][5]= TurnoverModelApp.holddata2[i+TurnoverModelApp.Agerepetitions+TurnoverModelApp.startage-numtoshow][2];

data[i][8]= TurnoverModelApp.holddata2[i+TurnoverModelApp.Agerepetitions+TurnoverModelApp.startage-numtoshow][3];

}

TurnoverModelApp.apopdurationhours=holdapop*1.2;

CompareHistogramsforVariousPatientAges(gender,ages,1,gtmult);

for (int i=0;i<numtoshow;i++){

data[i][3]= TurnoverModelApp.holddata2[i+TurnoverModelApp.Agerepetitions+TurnoverModelApp.startage-numtoshow][1];

data[i][6]= TurnoverModelApp.holddata2[i+TurnoverModelApp.Agerepetitions+TurnoverModelApp.startage-numtoshow][2];

data[i][9]= TurnoverModelApp.holddata2[i+TurnoverModelApp.Agerepetitions+TurnoverModelApp.startage-numtoshow][3];

}

TurnoverModelApp.fileWriter(data,numtoshow,10,"VaryApopHistogram-M");

gender = "Female"; ages = new int[3]; ages[0]=49;ages[1]=69;ages[2]=80;

TurnoverModelApp.apopdurationhours=holdapop*0.8;

CompareHistogramsforVariousPatientAges(gender,ages,1,gtmult);

for (int i=0;i<numtoshow;i++){

data[i][1]= TurnoverModelApp.holddata2[i+TurnoverModelApp.Agerepetitions+TurnoverModelApp.startage-numtoshow][1];

data[i][4]= TurnoverModelApp.holddata2[i+TurnoverModelApp.Agerepetitions+TurnoverModelApp.startage-numtoshow][2];

data[i][7]= TurnoverModelApp.holddata2[i+TurnoverModelApp.Agerepetitions+TurnoverModelApp.startage-numtoshow][3];

}

TurnoverModelApp.apopdurationhours=holdapop*1.0;

CompareHistogramsforVariousPatientAges(gender,ages,1,gtmult);

for (int i=0;i<numtoshow;i++){

data[i][2]= TurnoverModelApp.holddata2[i+TurnoverModelApp.Agerepetitions+TurnoverModelApp.startage-numtoshow][1];

data[i][5]= TurnoverModelApp.holddata2[i+TurnoverModelApp.Agerepetitions+TurnoverModelApp.startage-numtoshow][2];

data[i][8]= TurnoverModelApp.holddata2[i+TurnoverModelApp.Agerepetitions+TurnoverModelApp.startage-numtoshow][3];

}

TurnoverModelApp.apopdurationhours=holdapop*1.2;

CompareHistogramsforVariousPatientAges(gender,ages,1,gtmult);

for (int i=0;i<numtoshow;i++){

data[i][3]= TurnoverModelApp.holddata2[i+TurnoverModelApp.Agerepetitions+TurnoverModelApp.startage-numtoshow][1];

data[i][6]= TurnoverModelApp.holddata2[i+TurnoverModelApp.Agerepetitions+TurnoverModelApp.startage-numtoshow][2];

data[i][9]= TurnoverModelApp.holddata2[i+TurnoverModelApp.Agerepetitions+TurnoverModelApp.startage-numtoshow][3];

}

TurnoverModelApp.fileWriter(data,numtoshow,10,"VaryApopHistogram-F");

TurnoverModelApp.apopdurationhours=holdapop*1.0;

}

public void VaryHalfLifeforHistograms() throws IOException{

double gtmult=1;

int numtoshow = 25;double[][] data = new double[numtoshow][10]; //0=Age(25->1),1=Y-80%,2=Y-100%,3=Y-120%,4=M-80%,5=M-100%,6=M-120%,7=O-80%,8=O-100%,9=O-120%

for (int i=0;i<numtoshow;i++){data[i][0]=numtoshow-i;}

String gender = "Male"; int[] ages = new int[3]; ages[0]=49;ages[1]=69;ages[2]=74;

CompareHistogramsforVariousPatientAges(gender,ages,0.8,gtmult);

for (int i=0;i<numtoshow;i++){

data[i][1]= TurnoverModelApp.holddata2[i+TurnoverModelApp.Agerepetitions+TurnoverModelApp.startage-numtoshow][1];

data[i][4]= TurnoverModelApp.holddata2[i+TurnoverModelApp.Agerepetitions+TurnoverModelApp.startage-numtoshow][2];

data[i][7]= TurnoverModelApp.holddata2[i+TurnoverModelApp.Agerepetitions+TurnoverModelApp.startage-numtoshow][3];

}

CompareHistogramsforVariousPatientAges(gender,ages,1.0,gtmult);

for (int i=0;i<numtoshow;i++){

data[i][2]= TurnoverModelApp.holddata2[i+TurnoverModelApp.Agerepetitions+TurnoverModelApp.startage-numtoshow][1];

data[i][5]= TurnoverModelApp.holddata2[i+TurnoverModelApp.Agerepetitions+TurnoverModelApp.startage-numtoshow][2];

data[i][8]= TurnoverModelApp.holddata2[i+TurnoverModelApp.Agerepetitions+TurnoverModelApp.startage-numtoshow][3];

}

CompareHistogramsforVariousPatientAges(gender,ages,1.2,gtmult);

for (int i=0;i<numtoshow;i++){

data[i][3]= TurnoverModelApp.holddata2[i+TurnoverModelApp.Agerepetitions+TurnoverModelApp.startage-numtoshow][1];

data[i][6]= TurnoverModelApp.holddata2[i+TurnoverModelApp.Agerepetitions+TurnoverModelApp.startage-numtoshow][2];

data[i][9]= TurnoverModelApp.holddata2[i+TurnoverModelApp.Agerepetitions+TurnoverModelApp.startage-numtoshow][3];

}

TurnoverModelApp.fileWriter(data,numtoshow,10,"VaryHLhistogram-M");

gender = "Female"; ages = new int[3]; ages[0]=49;ages[1]=69;ages[2]=80;

CompareHistogramsforVariousPatientAges(gender,ages,0.8,gtmult);

for (int i=0;i<numtoshow;i++){

data[i][1]= TurnoverModelApp.holddata2[i+TurnoverModelApp.Agerepetitions+TurnoverModelApp.startage-numtoshow][1];

data[i][4]= TurnoverModelApp.holddata2[i+TurnoverModelApp.Agerepetitions+TurnoverModelApp.startage-numtoshow][2];

data[i][7]= TurnoverModelApp.holddata2[i+TurnoverModelApp.Agerepetitions+TurnoverModelApp.startage-numtoshow][3];

}

CompareHistogramsforVariousPatientAges(gender,ages,1.0,gtmult);

for (int i=0;i<numtoshow;i++){

data[i][2]= TurnoverModelApp.holddata2[i+TurnoverModelApp.Agerepetitions+TurnoverModelApp.startage-numtoshow][1];

data[i][5]= TurnoverModelApp.holddata2[i+TurnoverModelApp.Agerepetitions+TurnoverModelApp.startage-numtoshow][2];

data[i][8]= TurnoverModelApp.holddata2[i+TurnoverModelApp.Agerepetitions+TurnoverModelApp.startage-numtoshow][3];

}

CompareHistogramsforVariousPatientAges(gender,ages,1.2,gtmult);

for (int i=0;i<numtoshow;i++){

data[i][3]= TurnoverModelApp.holddata2[i+TurnoverModelApp.Agerepetitions+TurnoverModelApp.startage-numtoshow][1];

data[i][6]= TurnoverModelApp.holddata2[i+TurnoverModelApp.Agerepetitions+TurnoverModelApp.startage-numtoshow][2];

data[i][9]= TurnoverModelApp.holddata2[i+TurnoverModelApp.Agerepetitions+TurnoverModelApp.startage-numtoshow][3];

}

TurnoverModelApp.fileWriter(data,numtoshow,10,"VaryHLhistogram-F");

}

public void VaryHalfLifeforC14andPerc() throws IOException{

double hmult = 1.0;//halflife,

double gtmult=1.0;//expansion exponent

CompareAversaBergmannPercent(hmult,gtmult);//hmult, gtmult

}

public void CompareHistogramsforVariousPatientAges(String gender,int[] ages,double halflifemultiplier,double gtmult) throws IOException{

double[][] data = new double[TurnoverModelApp.Agerepetitions+TurnoverModelApp.startage+50][4];

for (int i=0;i<TurnoverModelApp.Agerepetitions+TurnoverModelApp.startage;i++){data[i][0]=TurnoverModelApp.Agerepetitions+TurnoverModelApp.startage-i;}

//Age Selection is Very Critical to Fit with Anversa Figure 7

int patientage=ages[0];

ComputeAgeHistograms(gender,patientage,halflifemultiplier,gtmult,1,true);

int start=TurnoverModelApp.Agerepetitions+TurnoverModelApp.startage-patientage;

for (int i=start;i<start+patientage;i++){data[i][1]=TurnoverModelApp.holddata[i-start][3];}

patientage=ages[1];

ComputeAgeHistograms(gender,patientage,halflifemultiplier,gtmult,1,true);

start=TurnoverModelApp.Agerepetitions+TurnoverModelApp.startage-patientage;

for (int i=start;i<start+patientage;i++){data[i][2]=TurnoverModelApp.holddata[i-start][3];}

patientage=ages[2];

ComputeAgeHistograms(gender,patientage,halflifemultiplier,gtmult,1,true);

start=TurnoverModelApp.Agerepetitions+TurnoverModelApp.startage-patientage;

for (int i=start;i<start+patientage;i++){data[i][3]=TurnoverModelApp.holddata[i-start][3];}

//Normalize to %

double c1 = 0; double c2=0; double c3=0;

for (int i=0;i<TurnoverModelApp.Agerepetitions+TurnoverModelApp.startage+50;i++){c1=c1+data[i][1];c2=c2+data[i][2];c3=c3+data[i][3];}

for (int i=0;i<TurnoverModelApp.Agerepetitions+TurnoverModelApp.startage+50;i++){data[i][1]=100*data[i][1]/c1;data[i][2]=100*data[i][2]/c2;data[i][3]=100*data[i][3]/c3;}

TurnoverModelApp.holddata2=data;TurnoverModelApp.holdnumrows2=TurnoverModelApp.Agerepetitions+TurnoverModelApp.startage;

TurnoverModelApp.fileWriter(data,TurnoverModelApp.Agerepetitions+TurnoverModelApp.startage,4,"HistogrambyPatientAge");

}

public double ComputeAveC14atDeath(int patient, String gender, int patientbirthyear,int patientdeathyear,double hmult,double gtmult, double apopmultfract,boolean recompute) throws IOException{

//Turn on for Anversa Histograms and Off for Bergmann

if (recompute){

ComputeAgeHistograms(gender,patientdeathyear-patientbirthyear,hmult,gtmult,apopmultfract,true);

}

double[][] riemanndata = TurnoverModelApp.convertHistotoRiemannSum(TurnoverModelApp.holddata, patientdeathyear-patientbirthyear);

double[][] data = new double[patientdeathyear-patientbirthyear][3]; //0=AgeofCM, 1=#CMProducedofAge, 2=C14atDateofProduction

for (int i=0;i<patientdeathyear-patientbirthyear;i++){

data[i][0]=riemanndata[i][0];

data[i][1]=riemanndata[i][1];

data[i][2]=GetC14atyear(patientbirthyear+i);

}

data[0][2]=TurnoverModelApp.getinitialbergmannpatientC14level(patient);

//TurnoverModelApp.fileWriter(data,patientdeathyear-patientbirthyear,3,"C14forPatient");

double CMcount=0; double C14count=0;

for (int i=0;i<patientdeathyear-patientbirthyear;i++){

CMcount=CMcount+data[i][1];C14count=C14count+data[i][1]*data[i][2];

}

double aveC14 = C14count/CMcount;

System.out.println(aveC14);

//System.out.println("Delta C14: "+(aveC14-GetC14atyear((double)(patientbirthyear))));

//Normalize for PrintOut

double c1 = 0;double[][] normdata = data;

for (int i=0;i<patientdeathyear-patientbirthyear;i++){c1=c1+normdata[i][1];}

for (int i=0;i<patientdeathyear-patientbirthyear;i++){normdata[i][1]=100*normdata[i][1]/c1;}

TurnoverModelApp.fileWriter(normdata,patientdeathyear-patientbirthyear,3,"/IndividC14/C14"+patientbirthyear+patientdeathyear);

return aveC14;

}

public void CompareAnversaBergmannC14(double hmult, double gtmult) throws IOException{

//double hmult=1.0;double gtmult=1.0;

double[][] patients = new double[12][3]; //Patient,Bergmann Raw Delta C14,AnversaPredictedDeltaC14

String gender="";

for (int i=0;i<12;i++){

patients[i][0]=TurnoverModelApp.Bergmann[i][0];

patients[i][1]=TurnoverModelApp.Bergmann[i][7];

if (TurnoverModelApp.Bergmann[i][1]==1){gender="Male";}else if (TurnoverModelApp.Bergmann[i][1]==0){gender="Female";}else{System.out.println("Gender Error"+TurnoverModelApp.Bergmann[i][2]);}

patients[i][2]=ComputeAveC14atDeath(i,gender,(int)TurnoverModelApp.Bergmann[i][2],(int)(TurnoverModelApp.Bergmann[i][2]+TurnoverModelApp.Bergmann[i][3]),hmult,gtmult,1,true);

patients[i][2]=patients[i][2]-TurnoverModelApp.getinitialbergmannpatientC14level(i);

// patients[i][2]=patients[i][2]-GetC14atyear(TurnoverModelApp.Bergmann[i][2]);

}

TurnoverModelApp.fileWriter(patients,12,3,"BergmannAnversaC14");

TurnoverModelApp.holddata=patients;TurnoverModelApp.holdnumrows=12;

}

public void CompareAversaBergmannPercent(double hmult, double gtmult) throws IOException{

CompareAnversaBergmannC14(hmult, gtmult);

double[][] data = new double[12][3]; //patient, Bergmann Reported %, Anversa-Produced Bergmann

double C14multiplier=1.0;

for (int i=0;i<12;i++){

data[i][0]=TurnoverModelApp.Bergmann[i][0];

data[i][1]=TurnoverModelApp.Bergmann[i][6];

data[i][2]=100*TurnDeltaC14intoScenarioATurnover(i,(int)TurnoverModelApp.Bergmann[i][2],(int)TurnoverModelApp.Bergmann[i][3],C14multiplier*TurnoverModelApp.holddata[i][2],TurnoverModelApp.initguess,true,1.0,0);

}

System.out.println("Finished");

TurnoverModelApp.fileWriter(data,12,3,"BergmannAnversaPerc");

}

public void ComputeAgeHistograms(String gender, int patientage,double halflifemultiplier,double gtmult,double apopfractmult, boolean addploidy) throws IOException{

double BergmannTurnover=0.01;double cellsinheart=500000000;

boolean senescencebool = false; //normally true, try false for trajectories

double[][] CMbyAge = new double[patientage][4];//0=AgeofCM, 1=Number of Age Births,2=N(a) No Apop Funct, 3=N(a)

//false turns off apoptosis so it can be handled with random kill applied to previous cycles below

HierarchicalModel10g(gender,false,true,senescencebool,gtmult,1,1);

for (int i=0;i<patientage;i++){CMbyAge[i][0]=patientage-i;}

///for (int i=0;i<patientage;i++){CMbyAge[i][1]=TurnoverModelApp.holddata[i][7];} //For Kajstura

//Make constant to match Bergmann (or age declining)

for (int i=0;i<patientage;i++){CMbyAge[i][1]=BergmannTurnover*cellsinheart;} //New CM in Year

for (int i=0;i<patientage;i++){

double n = 500*Math.pow(10,6);

//n = TurnoverModelApp.holddata[i][2]; //Total Myocyte Count Allowed to Vary with Age, Comment Out to Fix CM count at 500 Million

double a=CMbyAge[i][0]; //a = age of CM at TIME OF DEATH

double h;

h = TimetoSenescence(gender,i)/2; //Variable is required for older patients to have young CM. Reverse if h is constant

h = h*halflifemultiplier; //usually 1.0

CMbyAge[i][2]=n*Math.exp(-1*((Math.pow(a,2))/(2*Math.pow(h,2))));CMbyAge[i][3]=CMbyAge[i][2];

for (int j=0;j<=i;j++){

double deathcyclesperyear = 365*24/TurnoverModelApp.apopdurationhours;

for (int k=0;k<=deathcyclesperyear;k++){

CMbyAge[i][3] = CMbyAge[i][3]-(CMbyAge[j][2])*apopfractmult*ApopCMperMillionCM(gender,i)/Math.pow(10,6); //using i instead of j means i takes all death

}

}

if (CMbyAge[i][3]<0){CMbyAge[i][3]=0;}

}//patientage for loop

if (addploidy){ //Worst Case Scenario For Concordance - Adds All Ploidy to End

double TetraPerc = AnversaPloidyLinear4n(gender,patientage);double OctaPerc=AnversaPloidyLinear8n(gender,patientage);

CMbyAge[patientage-1][3]=CMbyAge[patientage-1][3]+TetraPerc*TurnoverModelApp.holddata[patientage-1][2]+2*OctaPerc*TurnoverModelApp.holddata[patientage-1][2];

}

TurnoverModelApp.holddata=CMbyAge;TurnoverModelApp.holdnumrows=patientage;

TurnoverModelApp.fileWriter(CMbyAge,patientage,4,"AgeHistogram");

}

public void HierarchicalModel10g(String gender,boolean includeapop,boolean includebirth, boolean includeCMsen,double gtmult, double apopfractmult,double apopdurationmult) throws IOException{

// System.out.println("GTmult"+gtmult);

int Agerepetitions = TurnoverModelApp.Agerepetitions;

int startage = TurnoverModelApp.startage;

double[][] CellTypesByAge = new double[Agerepetitions][8]; //0=Age,1=FunctCompCSCs,2=StartingCM,3=CMafterBirths,4=CMafterapop,5=CMafterCMSenescence(afterApopCM),6=PercTurnOver,7=NewCMinYear

double cyclemult = 1;

CellTypesByAge[0][0]=startage;

CellTypesByAge[0][1]=CSCper10gram(gender,startage);

if (gender.equals("Male")){CellTypesByAge[0][2] = TurnoverModelApp.num20yroldMaleCM10g;}else{CellTypesByAge[0][2] = TurnoverModelApp.num20yroldFemaleCM10g;}

if (gender.equals("Male")){CellTypesByAge[0][3] = TurnoverModelApp.num20yroldMaleCM10g;}else{CellTypesByAge[0][3] = TurnoverModelApp.num20yroldFemaleCM10g;}

if (gender.equals("Male")){CellTypesByAge[0][4] = TurnoverModelApp.num20yroldMaleCM10g;}else{CellTypesByAge[0][4] = TurnoverModelApp.num20yroldFemaleCM10g;}

if (gender.equals("Male")){CellTypesByAge[0][5] = TurnoverModelApp.num20yroldMaleCM10g;}else{CellTypesByAge[0][5] = TurnoverModelApp.num20yroldFemaleCM10g;}

startage++;

double CMbirthsPerCycle=0; double FunctCSCs=0;int deathcyclesperbirthcycle = (int)Math.rint((cyclemult*TurnoverModelApp.cyclelengthhours)/(apopdurationmult*TurnoverModelApp.apopdurationhours));

int cyclesperyear=(int) Math.rint(365*24/(cyclemult*TurnoverModelApp.cyclelengthhours));

for (int i=1;i<Agerepetitions;i++){

CellTypesByAge[i][7]=0; //NewCMinYear

CellTypesByAge[i][0]=startage;

//Starting CM = Last End (After Birth and Apop Removal but BEFORE Senescence BC Senescence is Measured Percent Found So Not Iterative)

CellTypesByAge[i][2]=CellTypesByAge[i-1][4];

//Prior to Birth/Death, Initialize to Start

CellTypesByAge[i][3]=CellTypesByAge[i][2];

CellTypesByAge[i][4]=CellTypesByAge[i][3]; //Initialize Prior to Apop

CellTypesByAge[i][5]=CellTypesByAge[i][4]*(1-PercP16ink4aPositiveCM(gender,startage)/100);;

for (int b=0;b<cyclesperyear;b++){ //Assumes CMbirthsPerCycle is constant throughout year

FunctCSCs=FunctCompCSCsper10g(gender,startage);

double LCCs = Math.pow(2,0)*FunctCSCs; //Right Now, No Expansion for LCC. All in Gt

double PercCycling = (CSCper10gram(gender,startage)*PercOfCSCcycling(gender,startage)/(FunctCompCSCsper10g(gender,startage)))/100;

//Don't divide by cycle length since per cycle

double gt = GTbyAge(gender,startage)*gtmult;

CMbirthsPerCycle =PercCycling*LCCs*Math.pow(2,gt);

//System.out.println(gt+" "+CMbirthsPerCycle);

//CMafterBirths

if (includebirth==true){

CellTypesByAge[i][3]=CellTypesByAge[i][3]+CMbirthsPerCycle;

CellTypesByAge[i][4]=CellTypesByAge[i][4]+CMbirthsPerCycle;

CellTypesByAge[i][5]=CellTypesByAge[i][5]+CMbirthsPerCycle;

CellTypesByAge[i][7]=CellTypesByAge[i][7]+CMbirthsPerCycle;//NewCMinYear Explicitly

}

//Subtract Apoptosis (Assumes apop cycle < prolif cycle)

//Assumes only CM apoptose

//Assumes Birth Occurs Before Apoptosis Starts & Newly Born CM Can't be Killed Right Away

if (includeapop==true){

for (int p=0;p<deathcyclesperbirthcycle;p++){

CellTypesByAge[i][4]=CellTypesByAge[i][4]-(apopfractmult*ApopCMperMillionCM(gender,startage)*(CellTypesByAge[i][4]/Math.pow(10,6)));

}

}

//Scale for Senescence of MYOCYTES after Birth and Apoptosis

if (includeCMsen==true){

CellTypesByAge[i][5]=CellTypesByAge[i][4]*(1-PercP16ink4aPositiveCM(gender,startage)/100);

}

}

//Compute Turnover

double CMper10g=0;

//If Assumption of Constant Denominator (500 Million CM per 10g)

if (gender.equals("Male")){CMper10g=TurnoverModelApp.num20yroldMaleCM10g;}else{CMper10g=TurnoverModelApp.num20yroldFemaleCM10g;}

if (TurnoverModelApp.curmethod.equals("AllowNumCellstoFluctuate")){

CMper10g =CellTypesByAge[i][2];System.out.println(i+" "+CellTypesByAge[i][7]+" "+CellTypesByAge[i][2]+" "+Math.round(100*CellTypesByAge[i][7]/CellTypesByAge[i][2]));

}

CellTypesByAge[i][6]=100*CellTypesByAge[i][7]/CMper10g;

startage++;

}

TurnoverModelApp.holddata=CellTypesByAge;TurnoverModelApp.holdnumrows=Agerepetitions;TurnoverModelApp.holdnumcol=8;

}

//Compiled Data Extraction

public double PercP16ink4aPositiveCM(String gender,double ageyears){

//From Kajstura Fig 1. Intercepts Estimated

double perc=0;

if (gender.equals("Female")){perc = 0.68*ageyears+0;}

else if (gender.equals("Male")){perc = 0.89*ageyears+5;}

return perc;

}

public double ApopCMperMillionCM(String gender,double ageyears){

//From Kajstura Fig 1. Intercepts Estimated

double apop=0;

if (gender.equals("Female")){apop = 1.23*ageyears-18;}

else if (gender.equals("Male")){apop = 1.31*ageyears+7;}

return apop;

//From Mallet

/* double apop=0;

if (gender.equals("Female")){apop = 42;}

else if (gender.equals("Male")){apop = 133;}

return apop;*/

}

public double CSCper10gram(String gender,double ageyears){

//From Kajstura Fig 2. Intercepts Estimated

double CSCs=0;

if (gender.equals("Female")){CSCs = 2.344*ageyears - 26;}//CSCs = 2.344*ageyears - 37;

else if (gender.equals("Male")){CSCs = 1.44*ageyears - 10;}

CSCs = CSCs*1000;

return CSCs;

}

public double PercOfCSCcycling(String gender,double ageyears){

//From Kajstura Fig 2. Intercepts Estimated

double perc=0;

if (gender.equals("Female")){perc = 0.0514*ageyears + 4.5;}//0.0514*ageyears + 4.1;

else if (gender.equals("Male")){perc = 0.0193*ageyears+5;}

return perc;

}

public double PercOfCSCsenescent(String gender,double ageyears){

//From Kajstura Fig 3. Intercepts Estimated

double perc=0;

if (gender.equals("Female")){perc = 0.59*ageyears - 3.6;}

else if (gender.equals("Male")){perc = 0.73*ageyears + 3.6;}

return perc;

}

public double FunctCompCSCsper10g(String gender,double ageyears){

//From Kajstura Fig 3. Intercepts Estimated

double CSCs=0;

if (gender.equals("Female")){CSCs = 918*ageyears + 0;}

else if (gender.equals("Male")){CSCs = 295*ageyears + 20000;}

return CSCs;

}

public double GTbyAge(String gender,double ageyears){

//From Kajstura Fig 3. Intercepts Estimated

double GT=0;

if (gender.equals("Female")){GT = -0.0091*ageyears + 5.8;}//-0.0086*ageyears + 6;

else if (gender.equals("Male")){GT = -0.0036*ageyears + 5.4;}

return GT;

}

public void GetKajsturaTurnoverDenominatorinHeart(){

double ave = 0; double numpoints = 0; double thisyear=0;

for (int i=20;i<100;i++){

thisyear=1000000*MillionsMyocyteFormationCombinedModelPer10gPerYear("Female",i)/AnnTurnoverCombinedModel("Female",i);

System.out.println(thisyear);

ave=ave+thisyear;numpoints++;

}

ave=ave/numpoints;

System.out.println("Ave: "+ave);

}

public double MillionsMyocyteFormationCombinedModelPer10gPerYear(String gender,double ageyears){

//From Kajstura Fig 6. Intercepts Estimated

double MillionsNewCMPerYear=0;

if (gender.equals("Female")){MillionsNewCMPerYear = 2.5*ageyears - 34;}

else if (gender.equals("Male")){MillionsNewCMPerYear = 1.0*ageyears + 0;}

return MillionsNewCMPerYear;

}

public double AnnTurnoverCombinedModel(String gender,double ageyears){

//From Kajstura Fig 6. Intercepts Estimated

double AnnPerc=0;

if (gender.equals("Female")){AnnPerc = 0.434*ageyears - 4;}

if(AnnPerc<0){AnnPerc=0;}

else if (gender.equals("Male")){AnnPerc = 0.2*ageyears + 0;}

return AnnPerc;

}

public double AnversaPloidyLinear4n(String gender, double ageyears){

//From Kajstura Fig 5. Points Estimated

double perc=0;

if (gender.equals("Female")){perc = 0.000259*ageyears + 0.001457;}

else if (gender.equals("Male")){perc = 0.000176*ageyears + 0.003647;}

return perc;

}

public double AnversaPloidyLinear8n(String gender, double ageyears){

//From Kajstura Fig 5. Points Estimated

double perc=0;

if (gender.equals("Female")){perc = 0.000063*ageyears + 0.002799;}

else if (gender.equals("Male")){perc = 0.000057*ageyears + 0.002910;}

return perc;

}

public double TimetoSenescence(String gender, double ageyears){

//From Kajstura Supp. Fig XIV. Intercepts Estimated

double tts=0;

if (gender.equals("Female")){tts = -0.16*ageyears + 17.4;}

else if (gender.equals("Male")){tts = -0.19*ageyears + 17.4;}

if (tts<0){tts=0;}

return tts;

}

public double PercPostMitNonSenCM(String gender, double ageyears){

//From Kajstura Fig 4. Intercepts Estimated

double tts=0;

if (gender.equals("Female")){tts = -0.69*ageyears + 100;}

else if (gender.equals("Male")){tts = -0.89*ageyears + 94;}

if (tts<0){tts=0;}

return tts;

}

public static double GetC14atyear(double year){

//Returns first datapoint of that exceeds year (Consider for Rounding)

double C14=-100; boolean found=false;

for (int i=0;i<TurnoverModelApp.numC14points;i++){

if (!found&&TurnoverModelApp.C14points[i][0]>=year){

//Average Over Year

int thisyear = (int)TurnoverModelApp.C14points[i][0];

int count=0;double c14=0;

for (int j=0;j<TurnoverModelApp.numC14points;j++){

if ((int)TurnoverModelApp.C14points[j][0]==thisyear){

c14=c14+TurnoverModelApp.C14points[j][1];count++;

}

}

C14=c14/count;

//Does Not Average Over Year

C14=TurnoverModelApp.C14points[i][1];

found=true;

return C14;

}

}

if (C14==-100){C14=TurnoverModelApp.C14points[TurnoverModelApp.numC14points-1][1];}

return C14;

}

public static void TestPloidy() throws IOException{

int numtotest=20;

double[][] data = new double[TurnoverModelApp.numBergmann][numtotest+1]; //each row is a patient, each column is i/10% of Bergmann ploidy applied

double perc=0;

for (int j=0;j<=numtotest;j++){

for (int i=0;i<TurnoverModelApp.numBergmann;i++){

data[i][j]=100*TurnDeltaC14intoScenarioATurnover(i,(int)TurnoverModelApp.Bergmann[i][2], (int)TurnoverModelApp.Bergmann[i][3],TurnoverModelApp.Bergmann[i][7],TurnoverModelApp.initguess,true,.1*j,0);

}

}

TurnoverModelApp.fileWriter(data, TurnoverModelApp.numBergmann, numtotest+1, "TestPloidy");

}

public static void TestScenarioA() throws IOException{

double[][] data = new double[TurnoverModelApp.numBergmann][3];//Patient,BergmannReported,BergmannScenA

double scale=1;double delay=TurnoverModelApp.delay;//Scale controls polyploidy magnitude, delay controls delay from C14 atmosphere to body

for (int i=0;i<TurnoverModelApp.numBergmann;i++){

double initguess=TurnoverModelApp.initguess;

//Use scale*TurnoverModelApp.Bergmann[i][7] for no ploidy correction, [i][4] for correction

data[i][2]=100*TurnDeltaC14intoScenarioATurnover(i,(int)TurnoverModelApp.Bergmann[i][2], (int)TurnoverModelApp.Bergmann[i][3],TurnoverModelApp.Bergmann[i][7],initguess,true,scale,0);

data[i][0]=i;data[i][1]=TurnoverModelApp.Bergmann[i][6];

}

TurnoverModelApp.holddata3=data;TurnoverModelApp.holdnumrows3=TurnoverModelApp.numBergmann; TurnoverModelApp.holdnumcol3=3;

TurnoverModelApp.fileWriter(data, TurnoverModelApp.numBergmann, 3, "ScenarioA");

}

public static void GlobalFit() throws IOException{

String scenario = "TVB-TVDR";

//global mode

GlobalFitRange(scenario,0,TurnoverModelApp.numBergmann);

//individual patient list mode

//for (int i=0;i<TurnoverModelApp.numBergmann;i++){GlobalFitRange(scenario,i,i+1); }

//single patient model

//GlobalFitRange(scenario,6,7);

//GetCMAgeHistogramwithGlobal();

}

public static void GlobalFitRange(String scenario,int spatient, int endpatient) throws IOException{

double lowesterr=999999999; double holdbesterr=0;

if (scenario.equals("A")){

double Aparam = 0.0; TurnoverModelApp.modelparams = new double[1];

for (int k=0;k<10000;k++){

TurnoverModelApp.modelerror=0;

for (int i=spatient;i<endpatient;i++){

TurnDeltaC14intoScenarioTurnover(i,(int)TurnoverModelApp.Bergmann[i][2],(int)TurnoverModelApp.Bergmann[i][3],Aparam,Aparam);

}

//System.out.println("B: "+Math.round(10000*Aparam)*0.01+"%, D: "+Math.round(10000*Aparam)*0.01+"%, E: "+TurnoverModelApp.modelerror);

if (TurnoverModelApp.modelerror<lowesterr){lowesterr=TurnoverModelApp.modelerror;TurnoverModelApp.modelparams[0]=Aparam;}

Aparam=Aparam+0.0001;

}

System.out.println("BF Turnover: "+100*TurnoverModelApp.modelparams[0]+"%, SSE="+lowesterr);

}

if (scenario.equals("B")){

double Bbirth = 0; double Bdeath=0; TurnoverModelApp.modelparams = new double[2];

for (int k=0;k<100;k++){

Bdeath=0;

for (int d=0;d<10;d++){

TurnoverModelApp.modelerror=0;

for (int i=spatient;i<endpatient;i++){

TurnDeltaC14intoScenarioTurnover(i,(int)TurnoverModelApp.Bergmann[i][2],(int)TurnoverModelApp.Bergmann[i][3],Bdeath,Bbirth);

}

if (TurnoverModelApp.modelerror<lowesterr){lowesterr=TurnoverModelApp.modelerror;TurnoverModelApp.modelparams[0]=Bbirth;TurnoverModelApp.modelparams[1]=Bdeath; }

//System.out.println("B: "+Math.round(10000*Bbirth)*0.01+"%, D: "+Math.round(10000*Bdeath)*0.01+"%, E: "+TurnoverModelApp.modelerror);

Bdeath=Bdeath+0.001;

}

Bbirth=Bbirth+0.001;

}

System.out.println("BF Turnover: B="+100*TurnoverModelApp.modelparams[0]+"%, D="+100*TurnoverModelApp.modelparams[1]+"%, SSE="+lowesterr);

}

if(scenario.equals("E2")){

TurnoverModelApp.modelparams = new double[2];

double gamma0low = 0; double gamma0high = .5;double gamma0increment = 0.01;

double gamma1low = 0.0; double gamma1high = 2;double gamma1increment = 0.05;

int gamma0reps = (int) Math.rint((gamma0high - gamma0low)/gamma0increment);

int gamma1reps = (int) Math.rint((gamma1high - gamma1low)/gamma1increment);

if (gamma0reps==0){gamma0reps=1;}if (gamma1reps==0){gamma1reps=1;}

double gamma0=gamma0low;double gamma1=gamma1low;

for (int k=0;k<gamma0reps;k++){ //controls gamma0

gamma1=gamma1low;

for (int d=0;d<gamma1reps;d++){//controls gamma1

TurnoverModelApp.modelerror=0;

for (int i=spatient;i<endpatient;i++){

TurnDeltaC14intoScenarioE2Turnover(i,(int)TurnoverModelApp.Bergmann[i][2],(int)TurnoverModelApp.Bergmann[i][3],gamma0,gamma1);

}

if (TurnoverModelApp.modelerror<lowesterr){lowesterr=TurnoverModelApp.modelerror;TurnoverModelApp.modelparams[0]=gamma0;TurnoverModelApp.modelparams[1]=gamma1; holdbesterr= TurnoverModelApp.modelerror; }

//System.out.println("G0: "+gamma0+", G1: "+gamma1+", E: "+TurnoverModelApp.modelerror);

gamma1=gamma1+gamma1increment;

}

gamma0=gamma0+gamma0increment;

}

System.out.println("BEST: Gamma0: "+TurnoverModelApp.modelparams[0]+", Gamma1: "+TurnoverModelApp.modelparams[1]+", E: "+holdbesterr);

}

if (scenario.equals("TVB-TVDR")){ //Birth increases with patient age, Annual Death rate of CM increases with patient age

double desiredDINTrange = 0.01; //0.05 = 0->5%

int numrepsDINT = 10;

double desiredBINTrange=0.1; //0.05 = 0->10%

int numrepsBINT = 10;

double desiredDSLOPErange=0.02; //0.005 = -0.005 -> 0.005, in per year

int numrepsDSLOPE = 10;

double desiredBSLOPErnage=0.02;//0.005 = -0.005 -> 0.005, in per year

int numrepsBSLOPE = 10;

double bslope = -1*desiredBSLOPErnage; double bint=0; double dslope=-1*desiredDSLOPErange; double dint=0.0; TurnoverModelApp.modelparams = new double[4];

numrepsBSLOPE=1;numrepsBINT=1;numrepsDSLOPE=1;numrepsDINT=1;

bslope = 0.001;

for (int b0=0;b0<numrepsBSLOPE;b0++){ //controls bslope

System.out.println("Progress:"+ (int)(100*b0/numrepsBSLOPE)+"%");

bint =0;

bint=0.025;

for (int b1=0;b1<numrepsBINT;b1++){ //controls bint

dslope=-1*desiredDSLOPErange;

dslope = 0.0046;

for(int d0=0;d0<numrepsDSLOPE;d0++){ //controls slope

dint=0;

dint = 0.0089;

for (int d1=0;d1<numrepsDINT;d1++){ //controls dint

TurnoverModelApp.modelerror=0; boolean throwout = false;

for (int i=spatient;i<endpatient;i++){

GfitCustomScenVaryDeath(i,(int)TurnoverModelApp.Bergmann[i][2],(int)TurnoverModelApp.Bergmann[i][3],bslope,bint,dslope,dint);

throwout=CheckfinalCMcountfract(i, bslope, bint, dslope, dint);

//System.out.println(TurnoverModelApp.modelerror);

}

if (TurnoverModelApp.modelerror<lowesterr&&throwout==false){

lowesterr=TurnoverModelApp.modelerror;TurnoverModelApp.modelparams[0]=bslope;TurnoverModelApp.modelparams[1]=bint;TurnoverModelApp.modelparams[2]=dslope;TurnoverModelApp.modelparams[3]=dint;

TurnoverModelApp.holddouble2=lowesterr;TurnoverModelApp.holddouble3=TurnoverModelApp.holddouble;//Test Scenario A

}

dint=dint+desiredDINTrange/numrepsDINT; TurnoverModelApp.holddouble=TurnoverModelApp.holddouble+0.0001;//Test Scenario A

}

dslope=dslope+2*desiredDSLOPErange/numrepsDSLOPE;

}

bint=bint+desiredBINTrange/numrepsBINT;

}

bslope=bslope+2*desiredBSLOPErnage/numrepsBSLOPE;

}

System.out.println("BF Turnover: Bs="+TurnoverModelApp.modelparams[0]+", Bi= "+TurnoverModelApp.modelparams[1]+", Ds: "+TurnoverModelApp.modelparams[2]+", Di: "+TurnoverModelApp.modelparams[3]+", SSE= "+lowesterr);

}

// System.out.println("FINISH A "+TurnoverModelApp.holddouble2+" "+TurnoverModelApp.holddouble3); //Test Scenario A

}//end global method

public static boolean CheckfinalCMcountfract(int patient, double bslope, double bint, double dslope, double dint){

int lifespan = (int)TurnoverModelApp.Bergmann[patient][3];

boolean throwout = false;

//compute CM at age 18

int stopage = 20;

double addedthisyear=0;double totalfractstopage=0;

for (int i=0;i<=stopage;i++){

if(i==0){addedthisyear=Math.pow((1-dint),stopage);}

if(i>0){ double form = bslope*i+bint; if(i==0){form=1;}double death = dslope*i+dint;addedthisyear = form*Math.pow((1-death),(stopage-i));}

if(addedthisyear>=0){totalfractstopage=totalfractstopage+addedthisyear;}//else{System.out.println("Model Caused Negative Annual Cell Change "+bslope+" "+bint+" "+dslope+" "+dint);}

}

//Compute CM at end of life

double totalfract = 0; addedthisyear=0;

for (int i=0;i<=lifespan;i++){

if(i==0){addedthisyear=Math.pow((1-dint),lifespan);}

if(i>0){ double form = bslope*i+bint; if(i==0){form=1;}double death = dslope*i+dint;addedthisyear = form*Math.pow((1-death),(lifespan-i));}

if(addedthisyear>=0){totalfract=totalfract+addedthisyear;}//else{System.out.println("Model Caused Negative Annual Cell Change "+bslope+" "+bint+" "+dslope+" "+dint);}

}

//Decide on suitability

// System.out.println(patient+" "+totalfractstopage+" "+totalfract);

//System.out.println(totalfract/totalfractstopage);

if(totalfract>totalfractstopage){throwout=true;}

if(stopage>lifespan){throwout=false;}//meaningless comparison otherwise

return throwout;

}

public static void GetC14forScenarioE2() throws IOException{

double gamma0=0.123;double gamma1=1.42;

for (int i=0;i<TurnoverModelApp.numBergmann;i++){

TurnDeltaC14intoScenarioE2Turnover(i,(int)TurnoverModelApp.Bergmann[i][2],(int)TurnoverModelApp.Bergmann[i][3],gamma0,gamma1);

}

}

public static void GetCMAgeHistogramwithGlobal() throws IOException{

double bslope=0.001;double bint=0.025;double dslope = 0.005; double dint=0.01; //newbestfit

//double bslope=0.001;double bint=0.03;double dslope = 0.004; double dint=0.02; //bestfit

//double bslope=0.0;double bint=0.03;double dslope = 0.004; double dint=0.025; //secondbestfit

//double bslope=-0.0004;double bint=0.03;double dslope = 0.002; double dint=0.035; //thirdbestfit

//double bslope=0;double bint=0.01;double dslope = 0; double dint=.01; //constant

double[] endC14= new double[TurnoverModelApp.numBergmann];

for(int i=0;i<TurnoverModelApp.numBergmann;i++){

endC14[i]=GfitCustomScenVaryDeath(i,(int)TurnoverModelApp.Bergmann[i][2],(int)TurnoverModelApp.Bergmann[i][3],bslope,bint,dslope,dint);

//System.out.println(endC14[i]);

}

}

public static double GfitCustomScenVaryDeath(int patient,int birthyear, int lifespan,double birthslope,double birthintercept,double deathslope,double deathintercept) throws IOException{

double[][] agedist = new double[lifespan+1][3]; //age, %CMatAge, C14atyear

//assume totally random turnover but variable death rate

agedist = new double[lifespan+1][3];

for (int j=0;j<=lifespan;j++){

agedist[j][0]=lifespan-j; //CM Age

//Allow Constant Birth and Constant Death Rate

double deathrate = deathintercept+deathslope*j;

//deathrate = TurnoverModelApp.holddouble; //Test Scenario A

if (j==0){agedist[j][1]=Math.pow((1-deathrate),lifespan);}

double birthrate = birthintercept+birthslope*j;

//birthrate = deathrate;//Test Scenario A

if (j>0){agedist[j][1]=birthrate*Math.pow((1-deathrate),lifespan-j);}

if(agedist[j][1]<0){agedist[j][1]=0;}

agedist[j][2]=GetC14atyear(birthyear+j);

}

//agedist[0][2]=TurnoverModelApp.getinitialbergmannpatientC14level(patient);

//Normalize to cell count

double tot=0;

for (int j=0;j<=lifespan;j++){tot=tot+agedist[j][1];}

for (int j=0;j<=lifespan;j++){agedist[j][1]=agedist[j][1]/tot;}

double[] aveC14postploidy=new double[lifespan+1];for (int w=0;w<lifespan+1;w++){aveC14postploidy[w]=agedist[w][2];}

for (int p=0;p<lifespan;p++){ //each cell-age cohort contributes a certain amount of C14. p = year of pt life

double aveC14foragegroup = agedist[p][2];

for (int q=p;q<lifespan;q++){ //q = years after cell born in patient life p

double percadded = computepersonalizedpolyploidyc14rate(patient, q, 1);

aveC14foragegroup = (aveC14foragegroup + percadded*agedist[q][2])/(1+percadded);

}

aveC14postploidy[p]=aveC14foragegroup;

}

double newaveC14=0;for (int k=0;k<=lifespan;k++){newaveC14 = newaveC14+agedist[k][1]*aveC14postploidy[k];}

double modeldC14 = newaveC14-GetC14atyear(birthyear);

//System.out.println(modeldC14+TurnoverModelApp.getinitialbergmannpatientC14level(patient));

// System.out.println(Math.pow(modeldC14-TurnoverModelApp.Bergmann[patient][7],2));

TurnoverModelApp.modelerror=TurnoverModelApp.modelerror+Math.pow(modeldC14-TurnoverModelApp.Bergmann[patient][7],2); //SSE

System.out.println(tot);

//TurnoverModelApp.fileWriter(agedist,lifespan+1,3,"/Individual/"+patient);

//System.out.println(newaveC14);

return newaveC14;

}

public static void TurnDeltaC14intoScenarioTurnover(int patient,int birthyear, int lifespan,double deathrate,double birthrate) throws IOException{

double[][] agedist = new double[lifespan+1][3]; //age, %CMatAge, C14atyear

//assume totally random turnover but variable death rate

agedist = new double[lifespan+1][3];

for (int j=0;j<=lifespan;j++){

agedist[j][0]=lifespan-j; //CM Age

//Allow Constant Birth and Constant Death Rate

if (j==0){agedist[j][1]=Math.pow((1-deathrate),lifespan);}

if (j>0){agedist[j][1]=birthrate*Math.pow((1-deathrate),lifespan-j);}

agedist[j][2]=GetC14atyear(birthyear+j);

}

//Normalize to cell count

double tot=0;

for (int j=0;j<=lifespan;j++){tot=tot+agedist[j][1];}

for (int j=0;j<=lifespan;j++){agedist[j][1]=agedist[j][1]/tot;}

double[] aveC14postploidy=new double[lifespan+1];for (int w=0;w<lifespan+1;w++){aveC14postploidy[w]=agedist[w][2];}

for (int p=0;p<lifespan;p++){ //each cell-age cohort contributes a certain amount of C14. p = year of pt life

double aveC14foragegroup = agedist[p][2];

for (int q=p;q<lifespan;q++){ //q = years after cell born in patient life p

double percadded = computepersonalizedpolyploidyc14rate(patient, q, 1);

aveC14foragegroup = (aveC14foragegroup + percadded*agedist[q][2])/(1+percadded);

}

aveC14postploidy[p]=aveC14foragegroup;

}

double newaveC14=0;for (int k=0;k<=lifespan;k++){newaveC14 = newaveC14+agedist[k][1]*aveC14postploidy[k];}

double modeldC14 = newaveC14-GetC14atyear(birthyear);

//double modeldC14 = newaveC14-TurnoverModelApp.getinitialbergmannpatientC14level(patient);

//System.out.println(Math.pow(modeldC14-TurnoverModelApp.Bergmann[patient][7],2));

TurnoverModelApp.modelerror=TurnoverModelApp.modelerror+Math.pow(modeldC14-TurnoverModelApp.Bergmann[patient][7],2); //SSE

}

public static void TurnDeltaC14intoScenarioE2Turnover(int patient,int birthyear, int lifespan,double gamma0,double gamma1) throws IOException{

double[][] celldist = new double[lifespan+1][3]; //0=CM age at death, 1=%survive till death, 2=%formation

//compute death

for(int a=0;a<=lifespan;a++){

celldist[a][0]=a;

double deathrate = gamma0*gamma1/(gamma1+a);

double liverate = 1-deathrate;

for (int j=0;j<a;j++){

liverate=liverate*(1-gamma0*gamma1/(gamma1+j));

}

celldist[a][1]=liverate;

//System.out.println(liverate);

}

//compute formation

double[] holdformed = new double[lifespan];

for(int k=0;k<lifespan;k++){

double formed=0;

if(k>0){

for (int j=0;j<k;j++){

int a = j+k;

double newdeath = gamma0*gamma1/(gamma1+a);

formed = formed+holdformed[j]*newdeath;

}

}

else{formed=1;}

holdformed[k]=formed;

//System.out.println(k+" "+holdformed[k]);

celldist[lifespan-k-1][2]=formed;

}

//System.out.println(" A Live to End Formed%");

for(int i=0;i<lifespan-1;i++){

//System.out.println(celldist[i][0]+" "+celldist[i][1]+" "+celldist[i][2]);

}

double[][] agedist = new double[lifespan+1][3];

for (int j=0;j<=lifespan;j++){

agedist[j][0]=lifespan-j; //CM Age

agedist[j][1]=celldist[lifespan-j][1]*celldist[lifespan-j][2];

agedist[j][2]=GetC14atyear(birthyear+j);

if(j==1){agedist[j][2]=TurnoverModelApp.getinitialbergmannpatientC14level(patient);}

}

for(int i=0;i<=lifespan;i++){

//System.out.println(agedist[i][0]+" "+agedist[i][1]+" "+agedist[i][2]);

}

//Normalize to cell count

double tot=0;

for (int j=0;j<=lifespan;j++){tot=tot+agedist[j][1];}

//System.out.println("TOT: "+tot);

for (int j=0;j<=lifespan;j++){agedist[j][1]=agedist[j][1]/tot;}

double[] aveC14postploidy=new double[lifespan+1];for (int w=0;w<lifespan+1;w++){aveC14postploidy[w]=agedist[w][2];}

for (int p=0;p<lifespan;p++){ //each cell-age cohort contributes a certain amount of C14. p = year of pt life

double aveC14foragegroup = agedist[p][2];

for (int q=p;q<lifespan;q++){ //q = years after cell born in patient life p

double percadded = computepersonalizedpolyploidyc14rate(patient, q, 1);

aveC14foragegroup = (aveC14foragegroup + percadded*agedist[q][2])/(1+percadded);

}

aveC14postploidy[p]=aveC14foragegroup;

}

double newaveC14=0;for (int k=0;k<=lifespan;k++){newaveC14 = newaveC14+agedist[k][1]*aveC14postploidy[k];}

double modeldC14 = newaveC14-GetC14atyear(birthyear);

System.out.println(Math.pow(modeldC14-TurnoverModelApp.Bergmann[patient][7],2));

//double modeldC14 = newaveC14-TurnoverModelApp.getinitialbergmannpatientC14level(patient);

TurnoverModelApp.modelerror=TurnoverModelApp.modelerror+Math.pow(modeldC14-TurnoverModelApp.Bergmann[patient][7],2); //SSE

}

public void TestHybridModelforBergmann() throws IOException{

double[][] data = new double[TurnoverModelApp.numBergmann][3];

//int i=4;

for (int i=0;i<TurnoverModelApp.numBergmann;i++){

data[i][0]=i;

data[i][1]=TurnoverModelApp.Bergmann[i][4]-TurnoverModelApp.Bergmann[i][9]; //How much Bergmann Corrected for Ploidy

data[i][2]=ComputeDeltaC14forConstantTurnover(i,TurnoverModelApp.Bergmann[i][6]/100, true)-ComputeDeltaC14forConstantTurnover(i, TurnoverModelApp.Bergmann[i][6]/100, false);

//System.out.println("A "+i+" "+ data[i][1]+" "+data[i][2]);

TurnoverModelApp.holddata3=data;TurnoverModelApp.holdnumrows3=TurnoverModelApp.numBergmann; TurnoverModelApp.holdnumcol3=3;

TurnoverModelApp.fileWriter(data, TurnoverModelApp.numBergmann, 3, "HybridModelPloidyCompProof");

}

}

public static double computepersonalizedpolyploidyc14rate(int patientID, int a, double ploidyscale){

//smooth - break into 10 parts and compute a CAGR

double d0 = 110.5; double theta = 7; double n=5.4;

double k=76;k=TurnoverModelApp.Bergmann[patientID][8];

double c14percrate=0;

//compute f(a)

double FofA=0;

if (a==0){FofA=d0;}

if (a>0){FofA=d0+k/(1+Math.pow((a/theta),-1*n));}

//compute f(a-1)

double FofAminus1=0;

if (a>1){FofAminus1=d0+k/(1+Math.pow(((a-1)/theta),-1*n));}

if(a==1){FofAminus1=d0;}

if(a==0){FofAminus1=d0;}

c14percrate = ((FofA-FofAminus1)/(FofAminus1));

c14percrate = c14percrate*ploidyscale;

//System.out.println(patientID+" "+a+" "+c14percrate);

return c14percrate;

}

public static double TurnDeltaC14intoScenarioATurnover(int patientid, int birthyear, int lifespan, double BergmanndeltaC14,double initguess, boolean subtract,double ploidyscale,double delay) throws IOException{

double guessturn = initguess; int numtries = 400;

double[][] agedist = new double[lifespan+1][3]; //age, %CMatAge, C14atyear

//assume totally random turnover

double error=0; double lastC14=0;

for (int i=0;i<numtries;i++){

//System.out.println("Guess "+guessturn);

agedist = new double[lifespan+1][3];

for (int j=0;j<=lifespan;j++){

agedist[j][0]=lifespan-j; //CM Age

if (j>0){agedist[j][1]=guessturn*Math.pow((1-guessturn),lifespan-j);}

else if (j==0){agedist[j][1]=Math.pow((1-guessturn),lifespan);}

agedist[j][2]=GetC14atyear(birthyear+j);

//System.out.println("R " + agedist[j][0]+" "+100*agedist[j][1]+" "+agedist[j][2]);

}

double[] aveC14postploidy=new double[lifespan+1];for (int w=0;w<lifespan+1;w++){aveC14postploidy[w]=agedist[w][2];}

if (subtract==true){ //remove ploidy

for (int p=0;p<lifespan;p++){ //each cell-age cohort contributes a certain amount of C14. p = year of pt life

double aveC14foragegroup = agedist[p][2];

for (int q=p;q<lifespan;q++){ //q = years after cell born in patient life p

double percadded = computepersonalizedpolyploidyc14rate(patientid, q, ploidyscale);

aveC14foragegroup = (aveC14foragegroup + percadded*agedist[q][2])/(1+percadded);

}

aveC14postploidy[p]=aveC14foragegroup;

}

}

double newaveC14=0;for (int k=0;k<=lifespan;k++){newaveC14 = newaveC14+agedist[k][1]*aveC14postploidy[k];}

double modeldeltaC14 = newaveC14-GetC14atyear(birthyear);double desireddeltaC14=TurnoverModelApp.Bergmann[patientid][7];

lastC14=newaveC14;

//System.out.println(patientid +", % "+guessturn+", New C14 "+ newaveC14+", ModelDc14 "+modeldeltaC14+ ", DesiredDC14 "+desireddeltaC14);

error = modeldeltaC14-BergmanndeltaC14;

TurnoverModelApp.modelerror=TurnoverModelApp.modelerror+Math.pow(error,2); //SSE

//System.out.println(guessturn+" "+error+" "+BergmanndeltaC14+ " "+modeldeltaC14);

double trylower = CheckDeltaC14intoScenarioATurnover(patientid,birthyear,lifespan,BergmanndeltaC14,guessturn*.98,subtract,ploidyscale,delay); //returns new modeldeltaC14

double tryhigher = CheckDeltaC14intoScenarioATurnover(patientid,birthyear,lifespan,BergmanndeltaC14,guessturn*1.02,subtract,ploidyscale,delay);

if (Math.abs(BergmanndeltaC14-trylower)<Math.abs(BergmanndeltaC14-tryhigher)){guessturn=guessturn*0.98;}

else if(Math.abs(BergmanndeltaC14 - trylower)>Math.abs(BergmanndeltaC14 - tryhigher)){guessturn = guessturn * 1.02;}

}

//System.out.println(lastC14);

return guessturn;

}

public static double CheckDeltaC14intoScenarioATurnover(int patientid,int birthyear, int lifespan, double BergmanndeltaC14,double initguess,boolean subtract,double ploidyscale,double delay) throws IOException{

double guessturn = initguess;

double[][] agedist = new double[lifespan+1][3]; //age, %CMatAge, C14atyear

agedist = new double[lifespan+1][3];

for (int j=0;j<=lifespan;j++){

agedist[j][0]=lifespan-j; //CM Age

//For Scenario A

if (j>0){agedist[j][1]=guessturn*Math.pow((1-guessturn),lifespan-j);}

else if (j==0){agedist[j][1]=Math.pow((1-guessturn),lifespan);}

//For Scenario B

// if (j>0){agedist[j][1]=guessturn*Math.pow((1-TurnoverModelApp.deathrate),lifespan-j);}

// else if (j==0){agedist[j][1]=Math.pow((1-TurnoverModelApp.deathrate),lifespan);}

agedist[j][2]=GetC14atyear(birthyear+j-delay);

}

double[] aveC14postploidy=new double[lifespan+1];for (int w=0;w<lifespan+1;w++){aveC14postploidy[w]=agedist[w][2];}

if (subtract==true){ //remove ploidy

for (int p=0;p<lifespan;p++){ //each cell-age cohort contributes a certain amount of C14. p = year of pt life

double aveC14foragegroup = agedist[p][2];double massadded=1;

for (int q=p;q<lifespan;q++){ //q = years after cell born in patient life p

double percadded = computepersonalizedpolyploidyc14rate(patientid, q,ploidyscale);

aveC14foragegroup = (aveC14foragegroup + percadded*agedist[q][2])/(1+percadded);

}

aveC14postploidy[p]=aveC14foragegroup;

}

}

double newaveC14=0;for (int k=0;k<=lifespan;k++){newaveC14 = newaveC14+agedist[k][1]*aveC14postploidy[k];}

double modeldeltaC14 = newaveC14-GetC14atyear(birthyear);

return modeldeltaC14;

}

public static void PrintBombFunction(boolean smoothed) throws IOException{

if (smoothed==false){

TurnoverModelApp.fileWriter(TurnoverModelApp.C14points,TurnoverModelApp.numC14points,2,"/Bomb");

}

else if(smoothed == true)

{

int range = 2006-1930;

double[][] bomb = new double[range][2];

for (int i=0;i<range;i++){

bomb[i][0]=1930+i;

bomb[i][1]=GetC14atyear(1930+i);

}

TurnoverModelApp.fileWriter(bomb,range,2,"/Bomb");

}

}

public static void VarydC14forBergmannTurnoverAnalysis() throws IOException{

//data is column for each (0.8, 1.0, 1.2); row for each patient

double[][] data = new double[TurnoverModelApp.numBergmann][3];

double initguess=TurnoverModelApp.initguess;

for (int i=0;i<TurnoverModelApp.numBergmann;i++){

data[i][0]=100*TurnDeltaC14intoScenarioATurnover(i,(int)TurnoverModelApp.Bergmann[i][2], (int)TurnoverModelApp.Bergmann[i][3],0.8*TurnoverModelApp.Bergmann[i][4],initguess,true,1,0);

data[i][1]=100*TurnDeltaC14intoScenarioATurnover(i,(int)TurnoverModelApp.Bergmann[i][2], (int)TurnoverModelApp.Bergmann[i][3],1.0*TurnoverModelApp.Bergmann[i][4],initguess,true,1,0);

data[i][2]=100*TurnDeltaC14intoScenarioATurnover(i,(int)TurnoverModelApp.Bergmann[i][2], (int)TurnoverModelApp.Bergmann[i][3],1.2*TurnoverModelApp.Bergmann[i][4],initguess,true,1,0);

}

TurnoverModelApp.fileWriter(data,TurnoverModelApp.numBergmann,3,"VaryBergmanndC14");

}

public void VaryExpansionExponentforTurnover() throws IOException{

double[][] data = new double[TurnoverModelApp.Agerepetitions-1][7]; //Patient Age, M-Exponent = 80%, F-Exponent = 100%, F-Exponent = 120%, F-Exponent = 80%, F-Exponent = 100%, F-Exponent = 120%

HierarchicalModel10g("Male",false,true,false,0.8,1,1);

for (int i=0;i<TurnoverModelApp.Agerepetitions-1;i++){data[i][0]=TurnoverModelApp.holddata[i+1][0];data[i][1]=TurnoverModelApp.holddata[i+1][6];}

HierarchicalModel10g("Male",false,true,false,1.0,1,1);

for (int i=0;i<TurnoverModelApp.Agerepetitions-1;i++){data[i][2]=TurnoverModelApp.holddata[i+1][6];}

HierarchicalModel10g("Male",false,true,false,1.2,1,1);

for (int i=0;i<TurnoverModelApp.Agerepetitions-1;i++){data[i][3]=TurnoverModelApp.holddata[i+1][6];}

HierarchicalModel10g("Female",false,true,false,0.8,1,1);

for (int i=0;i<TurnoverModelApp.Agerepetitions-1;i++){data[i][4]=TurnoverModelApp.holddata[i+1][6];}

HierarchicalModel10g("Female",false,true,false,1.0,1,1);

for (int i=0;i<TurnoverModelApp.Agerepetitions-1;i++){data[i][5]=TurnoverModelApp.holddata[i+1][6];}

HierarchicalModel10g("Female",false,true,false,1.2,1,1);

for (int i=0;i<TurnoverModelApp.Agerepetitions-1;i++){data[i][6]=TurnoverModelApp.holddata[i+1][6];}

TurnoverModelApp.fileWriter(data,TurnoverModelApp.Agerepetitions-1,7,"VaryExponentforTurnover");

}

public void BergmannSensitivityToTrueTurnover() throws IOException{

//patients: 0=patient, 1=BergOutputifTrueTurn=0.1%,2=BergOutputifTrueTurn=1.0%,3=BergOutputifTrueTurn=10%

//True Turn = Constant Through Life but Includes Cell Inter-Life CM Death

double[] truepercs = {0.1,0.2,0.4,0.75,1.0,1.5,1.75,2.0,2.5,3.0,4.0,5.0,6.0,7.0,8.0,9.0,10,12.5,15,17.5,20,25,30,35,40};

//double[] truepercs = {0.1,0.2,0.75,1.0,2.0,4.0,7.0,10,15,20,30,40};

//double[] truepercs = {22};

double[][] patients = new double[truepercs.length][TurnoverModelApp.numBergmann+2];

for (int k=0;k<truepercs.length;k++){patients[k][0]=truepercs[k];patients[k][1]=truepercs[k];}

// int i=0;

for (int i=0;i<TurnoverModelApp.numBergmann;i++){

System.out.println("1 "+i);

for (int k=0;k<truepercs.length;k++){

TurnoverModelApp.counter=i;

patients[k][i+2]=100*TurnDeltaC14intoScenarioATurnover(i,(int)TurnoverModelApp.Bergmann[i][2],(int)TurnoverModelApp.Bergmann[i][3], ComputeDeltaC14forConstantTurnover(i,truepercs[k]/100,true),TurnoverModelApp.initguess,true,1,0);

}

}

TurnoverModelApp.fileWriter(patients,truepercs.length,TurnoverModelApp.numBergmann+2,"BergSensitivity");

}

public void IdentifyBifurcation() throws IOException{

for (int i=0;i<TurnoverModelApp.numBergmann;i++){

double C14 = ComputeDeltaC14forConstantTurnover(i,0.2,true); //must send a DECIMAL (sending 10/100 for 10% will not work!)

System.out.println(C14);

}

}

public static void PrintC14forYearRange(int yearstart,int yearend){

// for (int i=0;i<yearend-yearstart;i++){ System.out.println(GetC14atyear(yearstart+i));}

for (int i=0;i<TurnoverModelApp.numBergmann;i++){System.out.println(GetC14atyear(TurnoverModelApp.Bergmann[i][2]));}

}

@SuppressWarnings("unchecked")

// <editor-fold defaultstate="collapsed" desc="Generated Code">

private void initComponents() {

mainPanel = new javax.swing.JPanel();

menuBar = new javax.swing.JMenuBar();

javax.swing.JMenu fileMenu = new javax.swing.JMenu();

javax.swing.JMenuItem exitMenuItem = new javax.swing.JMenuItem();

javax.swing.JMenu helpMenu = new javax.swing.JMenu();

javax.swing.JMenuItem aboutMenuItem = new javax.swing.JMenuItem();

statusPanel = new javax.swing.JPanel();

javax.swing.JSeparator statusPanelSeparator = new javax.swing.JSeparator();

statusMessageLabel = new javax.swing.JLabel();

statusAnimationLabel = new javax.swing.JLabel();

progressBar = new javax.swing.JProgressBar();

mainPanel.setName("mainPanel"); // NOI18N

javax.swing.GroupLayout mainPanelLayout = new javax.swing.GroupLayout(mainPanel);

mainPanel.setLayout(mainPanelLayout);

mainPanelLayout.setHorizontalGroup(

mainPanelLayout.createParallelGroup(javax.swing.GroupLayout.Alignment.LEADING)

.addGap(0, 400, Short.MAX_VALUE)

);

mainPanelLayout.setVerticalGroup(

mainPanelLayout.createParallelGroup(javax.swing.GroupLayout.Alignment.LEADING)

.addGap(0, 252, Short.MAX_VALUE)

);

menuBar.setName("menuBar"); // NOI18N

org.jdesktop.application.ResourceMap resourceMap = org.jdesktop.application.Application.getInstance(turnovermodel.TurnoverModelApp.class).getContext().getResourceMap(TurnoverModelView.class);

fileMenu.setText(resourceMap.getString("fileMenu.text")); // NOI18N

fileMenu.setName("fileMenu"); // NOI18N

javax.swing.ActionMap actionMap = org.jdesktop.application.Application.getInstance(turnovermodel.TurnoverModelApp.class).getContext().getActionMap(TurnoverModelView.class, this);

exitMenuItem.setAction(actionMap.get("quit")); // NOI18N

exitMenuItem.setName("exitMenuItem"); // NOI18N

fileMenu.add(exitMenuItem);

menuBar.add(fileMenu);

helpMenu.setText(resourceMap.getString("helpMenu.text")); // NOI18N

helpMenu.setName("helpMenu"); // NOI18N

aboutMenuItem.setAction(actionMap.get("showAboutBox")); // NOI18N

aboutMenuItem.setName("aboutMenuItem"); // NOI18N

helpMenu.add(aboutMenuItem);

menuBar.add(helpMenu);

statusPanel.setName("statusPanel"); // NOI18N

statusPanelSeparator.setName("statusPanelSeparator"); // NOI18N

statusMessageLabel.setName("statusMessageLabel"); // NOI18N

statusAnimationLabel.setHorizontalAlignment(javax.swing.SwingConstants.LEFT);

statusAnimationLabel.setName("statusAnimationLabel"); // NOI18N

progressBar.setName("progressBar"); // NOI18N

javax.swing.GroupLayout statusPanelLayout = new javax.swing.GroupLayout(statusPanel);

statusPanel.setLayout(statusPanelLayout);

statusPanelLayout.setHorizontalGroup(

statusPanelLayout.createParallelGroup(javax.swing.GroupLayout.Alignment.LEADING)

.addComponent(statusPanelSeparator, javax.swing.GroupLayout.DEFAULT_SIZE, 400, Short.MAX_VALUE)

.addGroup(statusPanelLayout.createSequentialGroup()

.addContainerGap()

.addComponent(statusMessageLabel)

.addPreferredGap(javax.swing.LayoutStyle.ComponentPlacement.RELATED, 226, Short.MAX_VALUE)

.addComponent(progressBar, javax.swing.GroupLayout.PREFERRED_SIZE, javax.swing.GroupLayout.DEFAULT_SIZE, javax.swing.GroupLayout.PREFERRED_SIZE)

.addPreferredGap(javax.swing.LayoutStyle.ComponentPlacement.RELATED)

.addComponent(statusAnimationLabel)

.addContainerGap())

);

statusPanelLayout.setVerticalGroup(

statusPanelLayout.createParallelGroup(javax.swing.GroupLayout.Alignment.LEADING)

.addGroup(statusPanelLayout.createSequentialGroup()

.addComponent(statusPanelSeparator, javax.swing.GroupLayout.PREFERRED_SIZE, 2, javax.swing.GroupLayout.PREFERRED_SIZE)

.addPreferredGap(javax.swing.LayoutStyle.ComponentPlacement.RELATED, javax.swing.GroupLayout.DEFAULT_SIZE, Short.MAX_VALUE)

.addGroup(statusPanelLayout.createParallelGroup(javax.swing.GroupLayout.Alignment.BASELINE)

.addComponent(statusMessageLabel)

.addComponent(statusAnimationLabel)

.addComponent(progressBar, javax.swing.GroupLayout.PREFERRED_SIZE, javax.swing.GroupLayout.DEFAULT_SIZE, javax.swing.GroupLayout.PREFERRED_SIZE))

.addGap(3, 3, 3))

);

setComponent(mainPanel);

setMenuBar(menuBar);

setStatusBar(statusPanel);

}// </editor-fold>

// Variables declaration - do not modify

private javax.swing.JPanel mainPanel;

private javax.swing.JMenuBar menuBar;

private javax.swing.JProgressBar progressBar;

private javax.swing.JLabel statusAnimationLabel;

private javax.swing.JLabel statusMessageLabel;

private javax.swing.JPanel statusPanel;

// End of variables declaration

private final Timer messageTimer;

private final Timer busyIconTimer;

private final Icon idleIcon;

private final Icon[] busyIcons = new Icon[15];

private int busyIconIndex = 0;

private JDialog aboutBox;

}
