# Supplementary material for: Hybrid Mathematical Model of Cardiomyocyte Turnover in the Adult Human Heart
Source: PLoS One. 2012 Dec 19;7(12):e51683. doi: 10.1371/journal.pone.0051683 (PMC3526650; doi:10.1371/journal.pone.0051683)
Supplement: Methods S1 — (DOCX) [file pone.0051683.s019.docx]

**Expanded Methods:**

Modeling Approach

A JAVA-based mathematical model heart was developed in which a modeled subject’s end-of-life CM age distributions, defined by the modeled subject’s lifespan and CM turnover dynamics could be computed. These age distributions were produced by initializing the model with a starting number of CMs and then iteratively (with one iteration per year in the subject lifespan) forming new CM and destroying existing CM in accordance with formation/destruction parameters either specified with direct numeric rates or computed from component parameters such as those enumerated in Figure 1. Under some model simulations, apoptotic elimination was formulated to target CMs selectively based on the CM age (iteration of CM formation in within the progression of iterations) whereas other model regimes assumed random elimination of CMs.

When a timeline of atmospheric C14 was included as a reference input and the model heart was assigned a finite birth (start) year on that timeline, each CM formed in the simulation was assigned a DNA C14 value roughly equal to the appropriate atmospheric C14 level at the time of CM formation but adjusted for polyploidization. Polyploidization adjustment was computed by, for each CM age group in the simulation, beginning with the atmospheric C14 level at time of CM age group formation and iteratively (for each year between from formation until subject death) replacing that C14 number with a weighted average of the number (weighted by 100%) and the atmospheric C14 level contemporary to the next iteration’s polyploidization event (weighted by the percentage of CM undergoing polyploidization at that stage in the subject’s life, as specified by the Adler/Bergmann sigmoidal polyploidization curve).

Under these conditions, each CM surviving to the end of the simulation contributed DNA C14 to an average CM DNA C14 value for the modeled subject. Subtracting the initial atmospheric C14 level (at the start of simulation) from this final C14 weighted average yielded a ΛC14 value associated with the modeled subject.

In some analyses, the inverse operation was performed—converting an experimentally measured ΛC14 value (or a simulated ΛC14 value) attributed to a subject, into a best fit turnover solution—the turnover dynamics that best produce the observed or target ΛC14 value. For this task, a numerical solver evaluated a wide range of turnover solutions (constant or time-varying depending on specified restrictions) by generating a time-of-death CM age distribution resulting from each potential turnover solution in the testable range. Then, for each CM age group in each hypothetical distribution, the C14 contributed by a CM in the age group was computed (with polyploidization correction as aforementioned. Multiplying the fractional representation of each CM age group by the polyploidization-adjusted C14 contribution of that age group yields a net ΛC14 value for the potential turnover solution. The turnover dynamics yielding the closest fit to the measured or target ΛC14 is selected as the best fit solution.

Sources of Input Parameters

Age-dependent, gender-dependent parameter values for the number of CSCs, fraction of such CSCs cycling at any given moment, number of CSC divisions prior to senescence, and fraction of CM undergoing apoptosis at any given moment were extracted from the Kajstura manuscript. Thus for any modeled subject of any age or gender, a single value for each of these parameters (derived from regression models of 74 subjects computed in the Kajstura document) could be referenced and incorporated into a dynamic model heart. The formula for single-iteration CM count change (Figure 1) was also obtained from the Kajstura manuscript.

Similarly, raw data regarding subject-specific year of birth, year of death, and CM DNA C14 levels for each of 12 subjects studied in the Bergmann manuscript were extracted. These CM DNA C14 levels were reported by Bergmann as differential C14 measurements (measured C14 relative to 1955, prior to the rise in atmospheric C14. These differential measurements were converted to ΛC14 values by subtraction from the initial C14 levels (also differentials from 1955 values) for use in the hybrid model. The polyploidization age progression formula was also obtained from the Bergmann manuscript.

Examination of Kajstura Manuscript in Isolation Using Hybrid Model

The Kajstura manuscript proposes two complementary modeling methods—a hierarchically-structured cell kinetics model (controlled by CSC cycling rates and CM apoptosis rates) and an age-structured cell kinetics model controlled by CM half-life and apoptosis rates). In the hiarchically-structured model, an initial cell population of 500 Million CM/10 g tissue was used to start the simulation. Then, for each year in the simulation, CM were iteratively created or destroyed based on the assumed modeling parameters (enumerated in Figure 1). For the age-structured model, CM age distributions at time of subject death were produced for simulated subjects (defined by age at time of death and gender) according to the formula utilized by Kajstura et al, a modified version of the McKendrick von Foerster population dynamics formula:

where n(a) is the number of CM of a given age present at time of autopsy (for 10 g tissue), N is the initial number of CM present in 10 g tissue, a is the age of the CM, h is the halflife of a CM, and µ(a,t) is a mortality function dependent on a and the subject age t. In the hybrid model, µ(a,t) is realized by iteratively computing for all t (0 to lifespan) the number of CM destroyed (given as “per million” values by Kajstura measurements) and deducting the destroyed CM from the then-current total.

The ability of the hybrid model to faithfully represent the Kajstura model was first assessed by inputting primary cell dynamic parameters (from the Kajstura document) into the hybrid model and observing concordance of the hybrid model turnover output with the turnover results published in the Kajstura manuscript (routines:“ProduceFig1Ecmdepletion(),” “HierarchModel10gCompareOnlyApop(),” “HierarchModel10gCompareOnlyBirthRateFig6b(),” “ProduceAnnTurnoverFig6c()”, “VaryApopRateforHistograms()”, “VaryHalfLifeforHistograms()”) for Figures S2 and S3 (where “VaryApopRateforHistograms()” and “VaryHalfLifeforHistograms()” reveal validation when apoptosis and halflife parameters are set to unity). Parameters were then individually varied to assess the impact of each parameter on resulting conclusions of turnover (routines: “VaryBirthCycleDuration(),” “KajsturaModelTurnover()”, “VaryApopRateforHistograms()”, “VaryHalfLifeforHistograms()”, “VaryExpansionExponentforTurnover()”) for Figures 2, S2-S8, S14, and S15.

Determining variable parameter sensitivity was conducted by running routine “ProduceAnnTurnoverFig6c()” under various parameter conditions (with the line regarding turnover definition, adjusting versus constant, either enabled or disabled. Evaluation of the Mallet data is performed by running the routine “ProduceAnnTurnoverFig6c()” while the routine “ApopPerMillionCM()” is commented to enable reference to the Mallet data rather than to the Kajstura data.

Examination of the Bergmann Manuscript in Isolation Using Hybrid Model

The ability of the hybrid model to faithfully emulate the Bergmann methodology for computation of turnover levels from ΛC14 data was measured by substituting the hybrid model inputs with the single turnover value concluded by Bergmann in the Bergmann document for each of the 12 Bergmann subjects (modeled hearts with birth date and lifespan equivalent to that of a Bergmann subject). After integrating atmospheric C14, polyploidization data, and turnover derivation methodology from the Bergmann document, closeness of hybrid model output turnover to input turnover was observed, allowing quantification of distortion caused by the hybrid modeling procedure (routines and strategy schematically enumerated in Figure S9).

To determine inherent sensitivity of the Bergmann algorithm, a wide range of input turnovers was supplied to the hybrid model (as substitution for CM formation/destruction parameters) and output turnover for each input was reported. (routine: “BergmannSensitivityToTrueTurnover()”).

The impact of polyploidization correction on turnover estimations for the 12 Bergmann subjects was performed by conducting simulations with scaled fractions of subject-specific polyploidization correction factors measured in the Bergmann document and reporting the outputted turnover estimation. (routine: “TestPloidy()” with TurnoverModelApp.getTakamatsuPloidy() enabled).

The impact of a delay in incorporation of atmospheric C14 into human tissue was performed by running the hybrid model with the bomb curve moved forward in time by the proposed delay (setting the TurnoverModelApp class global variable “delay” equal to the desired delay (in years) and adjusting the “TurnoverModelApp.getinitialC14()” method to appropriately delayed levels.

Effect of initial C14 values on the hybrid model was performed by running the hybrid model with initial C14 levels (generally obtained from 1-year smoothing of atmospheric C14 readings obtained by Levin^6,7^) with the minimal or maximal readings obtained during that 1-year period (by specifying values in the “TurnoverModelApp.getinitialC14()” method.

Finding Turnover Models Simultaneously Compatible with Bergmann and Kajstura Datasets

A global fit solver was produced to identify parameter combinations most consistent with given ΛC14 result for a given modeled subject heart. To test the global fit solver’s equivalence to the Bergmann global fit solver, model regimes utilized by Bergmann were subjected to the hybrid model global fit solver and optimum parameter selection and mean squared error were compared to those reported by Bergmann. For example, in the constant turnover scenario (Scenario A, Bergmann Supplement), where Myocyte Formation Rate(%) = Annual Death Rate(%) by assumption, the solver computed a best-fit annual turnover rate of 0.99% (B_s_ = D_s_ = 0, B_i_ = D_i_ = 0.99%) with a sum of squares error (SSE) of 45,168—highly comparable to the Bergmann solver’s result of 1.0% with an SSE of 27,770. As another example, the solver also identified a best-fit CM formation rate of 1.3% with best-fit destruction rate of 0.0%, SSE = 43,376, for Bergmann’s Scenario B—the regime where CM birth and destruction rates are constant but not necessarily equal, in which Bergmann concluded identical rates of 1.3% and 0.0% with SSE = 27000). (routine: “GlobalFit()” where the TVB-TVDR model can be tested by un-commenting all lines ending with “Test Scenario A” comments. Parameter ranges and incremenations can be adjusted manually. The parameter solutions display as System.out.println and must be run within an IDE or command prompt. Alternative solutions can be obtained by altering other various methods/variables in the TurnoverModelApp class such as TurnoverModelApp.delay (for applying a delay between atmospheric C14 and incorporation into human tissue), TurnoverModelApp.getinitialC14(), TurnoverModelApp.initguess (which controls whether the high or low solution is reported in instances where both exist), and polyploidization schedule function (controlled by: Computepersonalizedpolyploidyc14rate() routine).

Alternative model schema, inspired by the Kajstura conclusion of subject age (time)-increasing turnover with subject age (time)-increasing vulnerability of newly formed CMs to apoptosis, were evaluated for quality of fit with the Bergmann ΛC14 measurements in the 12 subject dataset from the Bergmann document, although the parameter sweep included negative, constant, and positive temporal dependence. Turnover levels for each age are then computed by dividing the expression B_i_+B_s_*(Subject Age) by the fraction of CM present at that age (which can be displayed by enabling the System.out.println() line containing the precursor text: “Tot:”, for each age to be plotted.

Hybrid Model Integration of Kajstura and Bergmann Models

The 12 Bergmann subjects were modeled (i.e. models were created using birth date and lifespans equivalent to the Bergmann subject set) with input turnover dynamics as prescribed by Kajstura or with turnover dynamics with best global fits to the Bergmann data (as identified by the global solver) to produce CM age histograms at time of modeled subject death. Expected ΛC14 levels corresponding to each of these CM age histograms were then produced for comparison to the ΛC14 observations made by Bergmann for these subjects (routines: GetCMAgeHistogramwithGlobal() for Best Fit and Bergmann models, and ComputeAveC14atDeath() for Kajstura model with “recomputed” set true and histograms printed to “IndividC14” folder).
